# Design Strategies and Roles of Hydrogels for Sustainable Energy Conversion and Harvesting from Natural and Biological Environments

**DOI:** 10.1002/adma.202510270

**Published:** 2025-09-29

**Authors:** Wanheng Lu, Wei Li Ong, Xinglong Pan, Zhiwei Li, Guo Tian, Ghim Wei Ho

**Affiliations:** ^1^ Department of Electrical and Computer Engineering National University of Singapore 4 Engineering Drive 3 Singapore 117583 Singapore; ^2^ Department of Materials Science and Engineering National University of Singapore 9 Engineering Drive 1 Singapore 117575 Singapore; ^3^ Institute of Materials Research and Engineering A*STAR (Agency for Science, Technology and Research) 3 Research Link Singapore 117602 Singapore

**Keywords:** moisture electricity generation, photocatalytic, photothermal, piezoionic, thermodiffusion, thermoelectric, thermogalvanic, triboelectric

## Abstract

The growing demand for sustainable energy has spurred interest in harvesting ambient sources, such as solar radiation, mechanical vibrations, water flow, and temperature gradients, and biological activities, such as motion and respiration. In this context, hydrogels have emerged as promising materials bridging natural and physiological energy environments. Known for their polymer networks and biocompatibility, hydrogels are widely used across bioengineering, biomedicine, and agriculture. Beyond these applications, hydrogels are also gaining attention in environmental and energy‐related technologies, including solar‐driven desalination, catalysis, and energy generation and storage. Their appeal lies in unique physicochemical properties, stimuli‐responsiveness, tunable interfacial chemistry, environmental benignity, and efficient mass and heat transfer while maintaining mechanical compatibility with hybrid or soft–hard systems. Despite these promising attributes, few reviews focus on the role of hydrogels in energy harvesting. This review addresses that gap by examining hydrogel‐based technologies driven by environmental stimuli and emphasizing their unique contributions to energy conversion. It offers insights into design strategies and recent advancements in functional hydrogels, highlighting opportunities and challenges in this field. As hydrogel‐based energy harvesting evolves, innovative design, deeper mechanistic understanding, and interdisciplinary integration are needed to unlock its potential.

## Graphical abstract

This review examines hydrogel‐based technologies driven by environmental stimuli and emphasizes their unique contributions to energy conversion. It provides insights into design strategies and recent advancements in functional hydrogels, highlighting opportunities and challenges in this field. As hydrogel‐based energy harvesting evolves, innovative design, deeper mechanistic understanding, and interdisciplinary integration are needed to unlock its potential.

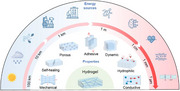

## 1 Introduction

As the global demand for sustainable and decentralized energy sources continues to grow, the pursuit of innovative materials and technologies for environmental energy harvesting has gained significant traction. This field presents a promising frontier, offering vast potential to utilize the abundant and diverse renewable resources available in our surroundings, such as solar radiation, mechanical vibrations, wind, water flow, and temperature gradients.^[^ ^1^, ^2^, ^3^, ^4^, ^5^, ^6^, ^7^, ^8^ ^]^ In parallel, the biological world offers additional, often overlooked, energy sources. Historical examples—such as electric discharge in eels,^[^ ^9^ ^]^ piezoelectric effects in bones,^[^ ^10^ ^]^ and triboelectric charges generated through friction^[^ ^11^ ^]^—demonstrate the potential of physiological systems for energy generation. With rapid progress in nanomaterials, microelectronics, bioelectronics,^[^ ^12^, ^13^, ^14^, ^15^, ^16^ ^]^ and human‐machine interface technologies,^[^ ^17^, ^18^, ^19^ ^]^ it has become increasingly feasible to harness energy from biological activities such as biochemicals,^[^ ^15^, ^16^ ^]^ motion,^[^ ^20^, ^21^ ^]^ perspiration,^[^ ^22^, ^23^ ^]^ respiration,^[^ ^24^ ^]^ and thermal emission.^[^ ^25^, ^26^, ^27^ ^]^ These bio‐derived energy sources complement conventional environmental inputs, opening new pathways for sustainable energy solutions in both natural and human‐centric settings.

Against this backdrop, hydrogels have emerged as a unique class of materials capable of bridging natural and physiological energy environments. Defined by their three‐dimensional polymer networks, high water content, tunable physicochemical properties, and inherent biocompatibility,^[^ ^28^ ^]^ hydrogels have traditionally found widespread use in bioengineering,^[^ ^29^, ^30^ ^]^ biomedicine,^[^ ^31^ ^]^ agriculture,^[^ ^32^, ^33^, ^34^ ^]^ and the food and cosmetic industries.^[^ ^34^, ^35^ ^]^ Recent innovations in hydrogel design have yielded materials that are strong, stretchable, resilient, self‐healing, conductive, semi‐conductive, and responsive to external stimuli.^[^ ^36^, ^37^, ^38^, ^39^, ^40^, ^41^ ^]^ These advances have dramatically expanded their application to fields such as human‐machine interfaces,^[^ ^17^, ^18^, ^36^, ^42^ ^]^ soft robotics,^[^ ^17^, ^43^, ^44^ ^]^ sensors, actuators,^[^ ^44^, ^45^ ^]^ coatings,^[^ ^46^ ^]^ and flexible electronics.^[^ ^27^, ^47^ ^]^ Beyond these applications, hydrogels are gaining attention for their roles in environmental and energy‐related technologies, including water treatment,^[^ ^48^ ^]^ solar‐driven desalination,^[^ ^49^ ^]^ atmospheric water harvesting,^[^ ^50^, ^51^ ^]^ catalysis,^[^ ^52^, ^53^ ^]^ cooling,^[^ ^54^ ^]^ smart windows,^[^ ^55^ ^]^ and, critically, energy generation and storage.^[^ ^56^, ^57^, ^58^ ^]^ Their compatibility with abundant, biodegradable, and biocompatible raw materials, along with scalable fabrication techniques like spinning and printing,^[^ ^59^ ^]^ enables green, economical, and sustainable hydrogel manufacturing. Collectively, these attributes position hydrogels as highly versatile, effective, and promising materials at the intersection of energy, environment, and biology.

Despite the growing interest in hydrogel materials, few review articles have comprehensively addressed their roles in environmental energy harvesting. Existing reviews often focus on hydrogel synthesis, modification, or general applications,^[^ ^57^, ^58^ ^]^ without a comprehensive or in‐depth discussion of energy conversion mechanisms. Other reviews primarily catalog hydrogel applications in energy generation and storage,^[^ ^60^, ^61^ ^]^ but do not highlight the unique roles that hydrogels play in diverse environmental energy harvesting technologies and their distinctive advantages in these processes. There are some reviews that mentioned several energy harvesting technologies,^[^ ^62^, ^63^ ^]^ but they fall short of discussing about cross‐energy‐modality integrated systems.

**Figure 1:**
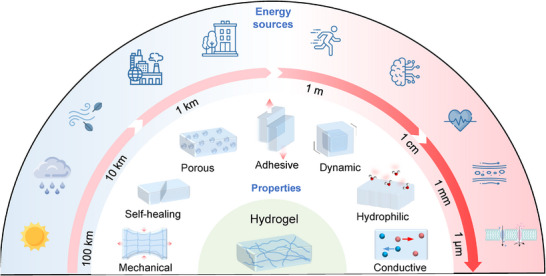
Overview of hydrogels for sustainable energy conversion and harvesting from natural and biological environments.

This review aims to fill that gap by providing a thorough overview of hydrogel‐based energy harvesting technologies. In this article, we start with a brief introduction to the synthesis and physicochemical properties of hydrogels (Section 2). Then we categorize and discuss these technologies according to the type of environmental stimulus—mechanical, thermal, chemical, and solar—and examine the underlying physical mechanisms, materials design strategies, and performance metrics (Section 3). Particular emphasis is placed on how hydrogels contribute to and enhance energy conversion processes, often enabling unique functionalities rarely achievable with conventional materials. To ground this review in practical experience, we also present recent advancements in the design and development of functional hydrogels and hydrogel‐based energy harvesting systems (Section 4). Drawing from years of research on nanomaterials for sustainable energy and fuel generation, we offer insights into both the opportunities and challenges facing this emerging field (Section 5). As hydrogel‐based energy harvesting is still in its formative stages, particularly in its integration with complex physiological systems, our discussion highlights the need for innovative design strategies and deeper mechanistic understanding to fully unlock its potential (**Figure** **1**).

## 2 Synthetic Routes and Physicochemical Properties of Hydrogels

This section provides an overview of hydrogel synthesis methods and their key physicochemical properties. Subsequently, the design principles underpinning their application in various energy generation technologies are systematically summarized.

### 2.1 Synthetic Routes of Hydrogels

Hydrogels are broadly classified into natural or synthetic hydrogels.^[^ ^64^ ^]^ Natural hydrogels are made of naturally available materials such as cellulose, chitosan, and alginate,^[^ ^65^, ^66^, ^67^ ^]^ while synthetic hydrogels are made from synthetic materials such as polyvinyl alcohol (PVA), poly (N‐isopropylacrylamide) (PNIPAM), and polyacrylamide (PAAm).^[^ ^68^, ^69^, ^70^ ^]^ Hydrogels can be formed by either physical or chemical cross‐linking of polymer chains. Physical cross‐linking tends to be weaker, and it can be achieved via hydrogen bonds, hydrophobic interactions, chain entanglement, crystallization, and protein interaction.^[^ ^71^ ^]^ On the other hand, chemically cross‐linked hydrogels are usually mechanically stronger due to covalent cross‐linking and involve chemical cross‐linking agents such as aldehydes or carbodiimides, free radical polymerization, and enzymes.^[^ ^72^ ^]^

The synthesis process of a hydrogel typically begins with the dissolution of a polymer in a solvent, followed by the addition of a catalyst or cross‐linker to initiate the cross‐linking and gelation process triggered by favorable conditions or stimuli such as temperature, pH, light, or heat.^[^ ^73^ ^]^ For example, gelatin hydrogels are formed when the aqueous gelatin solution is cooled below 35 °C as the Van der Waals forces between the collagen fibers increase in strength and overcome the thermal molecular vibrations. Hydrogels are usually cast in a mold of the desired size and shape, or cut into specific dimensions post‐synthesis, depending on the application. Alternatively, fibrous hydrogels can be fabricated via electrospinning and wet spinning^[^ ^74^, ^75^ ^]^ into a form factor that can be woven into a wearable textile to harness body motion.^[^ ^76^ ^]^ With the advent of advanced manufacturing in recent years, 3D printing and Direct Ink Writing (DIW) techniques^[^ ^77^ ^]^ have also emerged, enabling the customization of hydrogels with intricate designs and functions. All these fabrication methods can be easily scaled up for large‐scale continuous manufacturing production. However, to bring hydrogels to fruition as a real product, besides scalability, the fabrication process also has to be cost‐effective and sustainable. With the use of cheap raw materials and energy‐efficient synthesis methods, the manufacturing process can be cost‐effective. By opting for water‐based or solvent‐free polymerization methods and using biodegradable and non‐toxic chemicals and polymers, the manufacturing process can also be more sustainable.

In general, each synthesis and polymerization method has its unique advantages and disadvantages, and should be selected based on the application conditions and needs. A good understanding of the principles underlying hydrogel cross‐linking and networking will enable hydrogels with the desired properties to be synthesized.

### 2.2 Physicochemical Properties of Hydrogels

Hydrogels can be characterized by their mechanical properties, porosity, surface area, conductivity, hydrophilicity, crystallinity, adhesiveness, biocompatibility, self‐healing, water retention ability, and many others. In the utilization of hydrogels for harvesting environmental energy, such as mechanical vibrations, thermal/light energy, and chemical energy, it is important to optimize the design of the structure and tailor the properties towards achieving maximum response and performance. For example, the cross‐linking duration can be adjusted to control the stiffness of the hydrogel. Besides that, the inclusion of additives can also tune properties or introduce stimuli‐responsiveness. MXene sheets^[^ ^78^ ^]^ and ionic liquid^[^ ^79^ ^]^ are often added into the polymer matrix to enhance electrical conductivity, while polyaniline can be added as a photothermal material to promote light‐to‐heat conversion in the hydrogel.^[^ ^80^ ^]^ In this section, a brief guide is provided for the design of hydrogel properties for the various energy harvesting applications (**Table** **1**).

**Table 1:** Summary of hydrogel properties and design approaches for various energy harvesting applications.

Property	Energy harvesting application	Design approach
Mechanical	Mechanical vibrations Thermal energy Chemical potential Light energy	Cross‐link density Cross‐link type Molecular weight Network topology
Self‐healing	Mechanical vibrations	Dynamic covalent bonds Reversible physical bonds
Adhesion	Mechanical vibrations Thermal energy	Dipole‐dipole interaction Electrostatic interaction Mussel‐inspired polydopamine functionalized catechol groups
Biocompatibility	Mechanical vibrations Thermal energy	Cross‐link density Cross‐link type Molecular weight Natural polymers
Hydrophilic	Chemical potential Light energy	Hydrophilic groups Hygroscopic materials
Porosity	Chemical potential Light energy	Salt templating Porogen Foaming Freeze‐drying Emulsion templating Cross‐link density
Electrical and ionic conductivity	Thermal energy Chemical potential	Conductive fillers Ionic liquid
Stimuli‐responsive	Thermal energy Light energy	Thermo‐responsive polymer Photothermal material Photocatalyst additive

#### 2.2.1 Mechanical Properties

When hydrogels are used as a piezoionic or triboelectric generator, they will be subjected to physical deformation during the process of electricity generation.^[^ ^81^ ^]^ Hence, these hydrogels need to possess excellent mechanical strength, flexibility, elasticity, and stretchability so that they can endure repeated deformation cycles and recover to their original state without damage or mechanical fatigue.^[^ ^38^ ^]^ The mechanical properties of hydrogels can be affected by many factors, including polymer concentration, chemical structure and molecular weight of the polymer, network topology, and the nature and density of the cross‐linking.^[^ ^82^ ^]^

Polymers with higher molecular weight produce stronger hydrogels due to the increased interactions and entanglement between the polymer chains,^[^ ^83^ ^]^ while low molecular weight polymers result in gels that are less stiff. Similarly, a lower polymer concentration will also make the gel softer and less rigid.^[^ ^84^ ^]^ When the cross‐linking density is increased by incorporating a larger quantity of cross‐linking agents, it will also lead to higher mechanical strength of hydrogels,^[^ ^85^ ^]^ while lower cross‐linking density increases the mobility of the polymer chains, resulting in a softer, more flexible, and stretchable network that deforms more easily.^[^ ^86^ ^]^ Physical cross‐linking based on non‐covalent bonding between polymer chains usually leads to a more dynamic and less rigid network with higher stretchability, while chemical cross‐links made up of covalent bonds between polymer chains are typically stronger than physical cross‐links. Hence, the mechanical properties of natural polymers like alginate or chitosan can be enhanced by crosslinking or blending with synthetic polymers.^[^ ^87^ ^]^ Some strategies to achieve strong and tough hydrogels include fiber/fabric reinforced hydrogels,^[^ ^88^ ^]^ double network (DN) hydrogels,^[^ ^89^ ^]^ supramolecular‐interaction‐based hydrogels,^[^ ^90^ ^]^ hydrogels with well‐aligned microstructures,^[^ ^91^ ^]^ and design of topological structures to distribute stress in single‐network hydrogels such as slide‐ring gels.^[^ ^92^ ^]^

#### 2.2.2 Self‐Healing

In some cases, it may be necessary for the hydrogel to possess self‐healing properties to ensure the continuous functioning of the energy harvester. The self‐healing process can be described as the restoration of internal molecular bonds after they are broken, resulting in the recovery of the mechanical and rheological properties of the self‐repaired hydrogels.^[^ ^93^, ^94^ ^]^ These bonds are usually strong and reversible, and include physical cross‐links such as hydrogen bonding,^[^ ^95^, ^96^ ^]^ hydrophobic interactions,^[^ ^97^, ^98^ ^]^ metal coordination,^[^ ^99^ ^]^ host‐guest interactions,^[^ ^100^ ^]^ electrostatic interactions,^[^ ^101^ ^]^ and reversible chemical cross‐links like Schiff base bonds,^[^ ^102^ ^]^ hydrazone bonds,^[^ ^103^ ^]^ Diels‐Alder cycloaddition reactions,^[^ ^104^ ^]^ borate‐ester bonds,^[^ ^105^ ^]^ and disulfide bonds.^[^ ^106^ ^]^ A combination of physical and chemical cross‐links for self‐healing, such as hydrogen bonding with borate ester bonds^[^ ^107^ ^]^ or host–guest interactions with hydrazine bonds,^[^ ^108^ ^]^ is also often utilized. Depending on the type of bonds, external stimuli such as light,^[^ ^109^ ^]^ temperature,^[^ ^110^ ^]^ pH,^[^ ^111^ ^]^ magnetism,^[^ ^112^ ^]^ or salt^[^ ^113^ ^]^ might be required to drive the self‐healing process.

#### 2.2.3 Adhesion

When energy harvesters are designed to harness human motion, strong and conformal adhesion of the hydrogel to human skin is necessary to prevent delamination during human movement. Instead of using conventional adhesives, self‐adhesive hydrogels are preferred because they allow robust and seamless tissue‐device adhesion, providing comfort, reliability, conformal contact with skin, and reduced interface resistance with stable signal generation.^[^ ^114^ ^]^ Self‐adhesive hydrogels are typically based on mussel‐inspired polydopamine functionalized catechol groups to form covalent or noncovalent bonds with various surfaces.^[^ ^115^, ^116^ ^]^ Furthermore, polyelectrolyte hydrogels also exhibit self‐adhesion due to the dipole–dipole interactions and electrostatic interactions between the positive and negative charges of polyelectrolytes and the surface polar groups of other materials.^[^ ^117^, ^118^, ^119^ ^]^ These adhesive bonds possess advantages such as strong adhesion for long‐term repeated usage, and also retention of adhesion even in wet conditions, such as the presence of body fluids.

#### 2.2.4 Biocompatibility

Energy harvesters that are attached to the human body or skin to harness motion, heat, or even moisture from the breath need to be biocompatible to achieve optimal interfacing and to avoid adverse biological interactions and outcomes. In other words, the mechanical characteristics should match those of human skin, and the chemical properties should be bio‐friendly. Hydrogels are highly suitable because their mechanical and chemical properties closely resemble those of biological tissues, and can be tuned with great versatility and flexibility. The desired mechanical properties of hydrogels can be achieved by modifying factors such as polymer concentration, chemical structure, and the nature and density of the cross‐linking, depending on the requirements of the application. In terms of chemical compatibility, natural polymers such as cellulose, chitosan, alginate, gelatin, collagen, and hyaluronic acid that are non‐toxic have to be used.^[^ ^120^ ^]^ Some synthetic polymers, such as poly(vinyl alcohol) (PVA), poly(lactic acid) (PLA), and polyethylene glycol (PEG), can also be used.^[^ ^121^ ^]^ Another strategy is to combine synthetic hydrogels with natural polymers to create hybrid hydrogels with enhanced biocompatibility.^[^ ^122^ ^]^ Most importantly, toxic chemicals must be avoided in the preparation of the hydrogel, and the additives incorporated into the hydrogel have to be biocompatible.

#### 2.2.5 Hydrophilicity

Hydrogels are ideal for energy generation from moisture due to their hydrophilic nature, excellent moisture absorption ability, and ionic conductivity. Polymers such as polyvinyl alcohol (PVA),^[^ ^123^ ^]^ polyacrylamide (PAAm),^[^ ^124^ ^]^ alginate, chitosan and sulfated cellulose nanofibers (SCNF) with hydrophilic polymer chains are usually used because the hydroxyl (─OH), amino (─NH_2_), carboxyl (─COOH), and sulfonic acid (─SO_3_H) functional groups enable excellent capture of water molecules from moisture, enhancing the energy generation performance.^[^ ^125^ ^]^Additionally, hygroscopic materials such as solid desiccants and liquid sorbents can be incorporated into hydrogels using techniques such as immersion drying,^[^ ^126^ ^]^ ion exchange, impregnation network,^[^ ^127^ ^]^ in situ copolymerization,^[^ ^128^ ^]^ and solvent displacement,^[^ ^129^ ^]^ to further enhance the moisture absorption ability. Solid desiccants such as deliquescent salts (e.g., lithium chloride and calcium chloride)^[^ ^130^ ^]^ and metal–organic frameworks (MOFs) possess open binding sites to bond with water molecules.^[^ ^131^ ^]^ Liquid sorbents (e.g., concentration salt solutions, ionic liquids, and organic alcohols)^[^ ^132^, ^133^, ^134^ ^]^ exhibit hygroscopic properties due to their lower equilibrium water vapor pressure compared to the ambient.

#### 2.2.6 Porosity

The porosity of the hydrogel can affect the performance of the energy harvesters. Larger pore sizes usually lead to higher moisture absorption rates and greater power output in moisture‐electric generators,^[^ ^135^ ^]^ while aligned pores are preferred over random pore structure in solar steam generation.^[^ ^136^ ^]^ There are several ways to tune the porosity of hydrogels, and it can be optimised for different energy harvester applications. One way is by controlling the cross‐linking density, where a higher cross‐linking density leads to a denser, less porous structure, while a lower cross‐linking density results in more pores.^[^ ^137^ ^]^ Another approach is to use sacrificial templates such as salt crystals or porogens to create pores in the hydrogel. Salt crystals such as sodium chloride (NaCl) can be incorporated into the hydrogel and then dissolved to leave behind pores.^[^ ^138^ ^]^ Gas bubbles can be introduced into the hydrogel mixture through gas forming to form pores during the gelation process.^[^ ^139^ ^]^ There are also physical methods, such as freeze‐drying, where the hydrogel is frozen and the ice is then removed through sublimation, leaving behind pores.^[^ ^140^ ^]^ This freeze‐drying technique is frequently used to create oriented pores in hydrogels through directional freezing of the water in the hydrogel. Emulsion templating is another physical method used to create hydrogels with specific porous structures. In this method, two immiscible liquids are mixed together to create an emulsion template, where one phase is dispersed in the other, the continuous phase is then polymerized to form the solid matrix, and the dispersed phase is removed, leaving behind a porous structure with interconnected pores.^[^ ^141^ ^]^ Osmotic dehydration is another process in which the hydrogel is exposed to a hypertonic solution which will extract water from the hydrogel and create pores in a reproducible manner.^[^ ^142^ ^]^

#### 2.2.7 Electrical and Ionic Conductivity

Hydrogels are naturally poor conductors of electricity due to the insulating nature of commonly used hydrophilic polymer chains. However, conductive additives or electrolytes can be added to enhance the conductivity. Besides metal nanoparticles such as gold or silver,^[^ ^143^ ^]^ conductive fillers such as carbon nanotubes, graphene, and MXene^[^ ^144^ ^]^ can also be incorporated into the hydrogel matrix to create a continuous network of electronic conduction throughout the hydrogel framework.^[^ ^145^ ^]^ Conductive polymers such as polyaniline (PANI), polypyrrole (PPy), and poly(3,4‐ethylenedioxythiophene):poly(styrene sulfonate) (PEDOT:PSS)^[^ ^146^ ^]^ are also frequently used to increase the conductivity of the hydrogel. They provide an electrically conductive path through the delocalized π‐electrons of their conjugated systems, and also increase the ionic conductivity of the aqueous phase by contributing ions.^[^ ^147^ ^]^ In applications where ionic hydrogels are required, conductive polymers (polyelectrolyte) such as polyacrylamides (PAAm), polyphosphates, polyacrylic acid (PAA),^[^ ^148^ ^]^ and modified natural polymers can be used in the hydrogel. Polyelectrolytes contain positively or negatively charged groups that can dissociate in water to form charged polyions, and exhibit properties of both polymers and electrolytes. Besides using polyelectrolytes, another approach is to infuse neutral polymeric networks with ions such as sodium chloride (NaCl) solution.^[^ ^56^ ^]^

#### 2.2.8 Stimuli‐Responsivity

Hydrogels can be designed to respond to various stimuli by integrating active materials or by selecting suitable polymers. In order to harvest thermal energy, hydrogels can be synthesized from thermo‐responsive polymers such as poly(N‐isopropylacrylamide) (PNIPAM), which exhibits reversible phase change in response to temperature changes. When PNIPAM is heated above its lower critical solution temperature (LCST ≈32 °C), it undergoes a hydrophilic to hydrophobic phase change accompanied by a coil‐to‐globule transition.^[^ ^149^, ^150^, ^151^ ^]^ This behavior can be harnessed for thermally induced water expulsion^[^ ^51^ ^]^ or mechanical actuation.^[^ ^77^ ^]^ Hydrogels can also be incorporated with photothermal materials that respond to light stimuli and convert it into heat. There is a wide variety of photothermal materials such as metallic nanostructures (Au, Ag, and Cu),^[^ ^152^, ^153^ ^]^ carbonaceous materials (graphene, carbon nanotubes (CNTs), and carbon black),^[^ ^154^, ^155^, ^156^, ^157^ ^]^ and organic polymers with conjugated structures such as polypyrrole (PPy) and polydopamine (PDA).^[^ ^158^ ^]^ Photocatalytic materials such as TiO_2_ can be added into the hydrogel to generate a response to light irradiation,^[^ ^159^ ^]^ serving as a versatile platform for the generation of clean fuels such as hydrogen gas.

## 3 Hydrogels for Environmental Energy Harvesting

**Figure 2:**
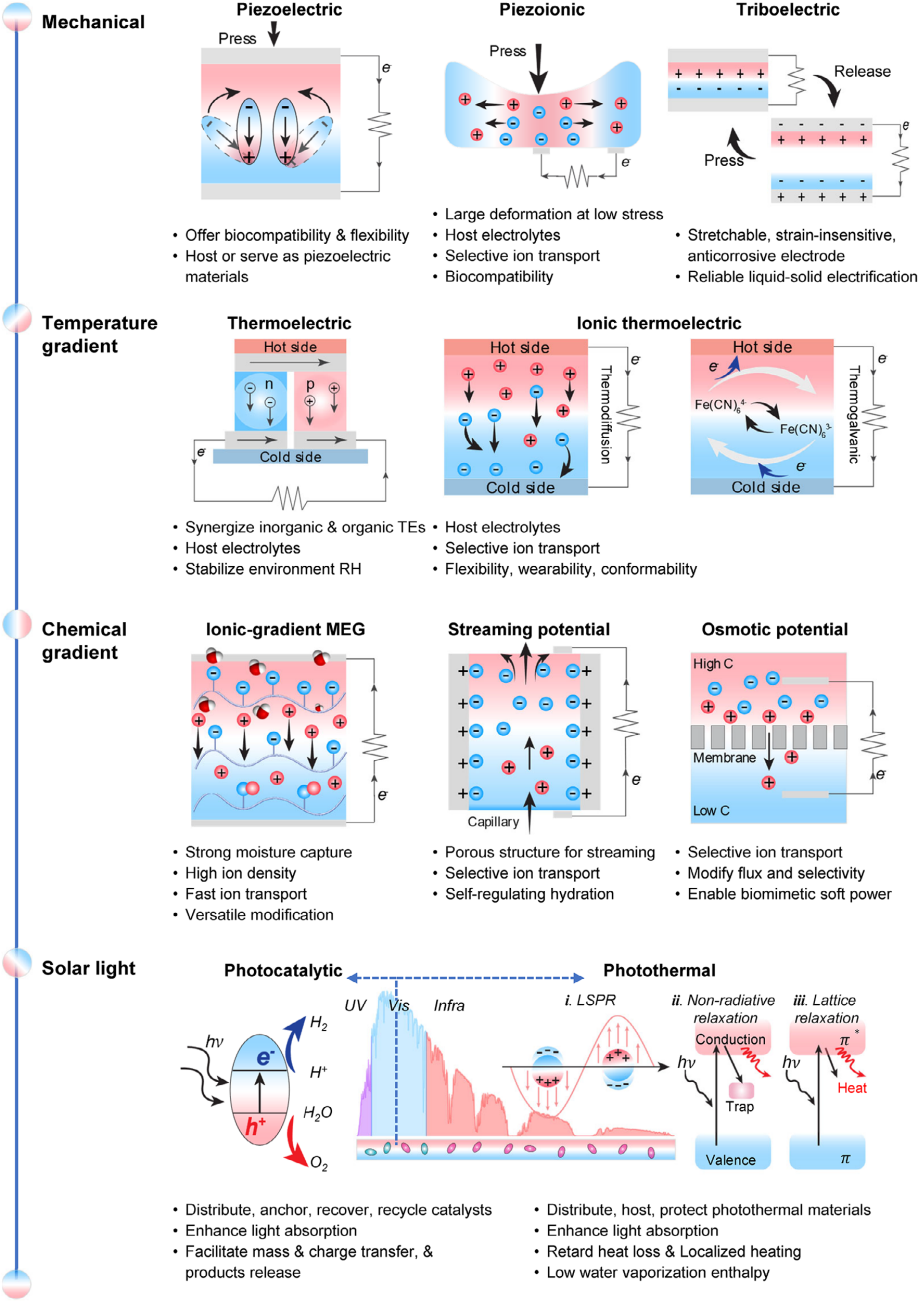
Roles of hydrogel in various energy harvesting technologies.

The unique properties of hydrogels, such as mechanical flexibility, water affinity, and physiological biocompatibility, make them ideal for energy harvesting, particularly in water‐rich environments or applications, like wearable, implantable electronics or soft robotics.^[^ ^56^, ^57^, ^160^, ^161^, ^162^, ^163^, ^164^, ^165^, ^166^ ^]^ This section reviews the diverse roles of hydrogels in converting environmental energy into electricity and fuel, focusing on environmental energy sources such as mechanical vibrations, temperature gradients, chemical potentials (moisture, hydrovoltaic, salinity), and solar light. It highlights the distinct advantages that hydrogels offer in these energy harvesting technologies (**Figure** **2**).

### 3.1 Conversion of Mechanical Vibrations

Mechanical vibrations, generated by natural and human‐induced activities like wave shocks, machine oscillations, human movements, and pulse beats, represent a ubiquitous and abundant energy source. Two of the most common technologies for harvesting this energy are piezoelectrics and triboelectrics.^[^ ^4^, ^8^, ^167^, ^168^, ^169^ ^]^ Due to their unique properties, including biocompatibility, flexibility, high water/solvent content, mixed electronic‐ionic conductivity, quasi‐solid structure, and extensive modification potential, hydrogels have been explored as components or in combination with piezoelectric and triboelectric materials for converting mechanical vibrations into usable electricity. In certain instances, they have even facilitated the derivation of novel technology such as piezoionics, enabling the harvesting of previously untapped energy sources like low‐frequency vibrations.^[^ ^161^, ^162^ ^]^ A summary of relevant works is presented in **Table** **2** at the end of this sub‐section.

**Table 2:** Summary of hydrogels for natural and biological environmental energy harvesting—Mechanical vibration.

Mechanisms	Key roles played by hydrogels	Hydrogels' properties required	Typical Hydrogels: Synthesis and Performance	Applications	Refs.
**Piezoelectric**	Soft matrix for piezoelectric materials Also serve as piezoelectric materials	Biocompatible, flexible, mechanical strength Piezoelectric	ZnO‐silk fibroin hydrogel, enzyme‐catalyzed gelation Compression strength: 1.82 MPa Energy output: 80 mV, 32 nA at 0.1 MPa stress	Self‐powered sensor Bone healing regeneration	[178]
			PVDF/PEDOTS/Chitosan, one‐pot thermoforming and solution exchange Elongation up to 293% Energy output: 100 mV	Sensors for human activity	[181]
**Piezoionic**	Host electrolyte Large deformation at low stress Ionic selectivity Biocompatibility	High hydration,“solid liquid” Soft, low Young's modulus Controllable porous structure chemical properties Biocompatible	Polyacylamide (PAAm) hydrogels loaded with NaCl solution		[56]
			Polyacrylic acid (PAA) hydrogel Energy output: 6 mV, 2 µA under a force of 5N	Sensors	[148]
**Triboelectric**	Stretchable, strain‐insensitive, anticorrosive Reliable liquid‐solid electrification	Flexible, stretchable, ionic conductive “solid liquid”	Polyacrylamide (PAAm) hydrogel loaded with LiCl solution Stretchability: 1160% Energy output: 145 V, 1.5 µA, 35 mW m^−2^ under 100 kPa & 1.5 Hz	TENG electrodes	[197]
			Polyacrylamide (PAAm) hydrogel with β‐cyclodextrin molecules Anticorrosive, stable under acidic, alkaline, and high salt‐concentration conditions Energy output: 95 V, 38 nC, 10 µA, 63.5 mW m^−2^ at 2 Hz	TENG electrodes	[200]
			Polyacrylamide‐sodium alginate (PAAm‐alginate) hydrogel Anticorrosive, stable under acidic, alkaline, and high salt‐concentration conditions Energy output: 70 V, 23.4 nC, 0.46 µA, 135 mW m^−2^ under 100 kPa &1.5 Hz	TENG electrodes	[196]
			Polyvinyl alcohol (PVA) hydrogel with Ni fabric Biodegradable PVA hydrogel provides flexibility, recyclablility, and sustainability Energy output: 200 V, 0.25 µC, 0.46 µA, 2 mW	TENG electrodes	[160]
			MXene/PVA hydrogel stretchability: 1800%, self‐healing Energy output: 230 V, 38 nC, 0.27 µA, 0.33 mW m^−2^	TENG electrodes	[78]
			Polyacrylamide (PAAm), mold casting Enable broad working bandwidth of 0–80 Hz by a solid/solid‐liquid contact Energy output: 0.62 V & 1 µW (7 gels at 60 Hz), 30 nC & 0.7 µA (6 gels at 10 Hz)	TENG active materials	[198]
			Polyacrylamide (PAAm) with pyramid arrays, mold casting Energy output: 6.5 V, 3 nC, 115 nA, 1 mW m^−2^	TENG active materials	[207]
			Poly (methyl acrylate) (PMA) / P(NAGA‐co‐AAm) hydrogel, dry‐to‐wet spinning Tensible strength: 2.27 MPa, stretchability: 900% Energy output: 36 V, 10 nC, 0.7 mA, 88 mW m^−2^	TENG active materials – textile	[76]

#### 3.1.1 Piezoelectric Energy Harvesting

The piezoelectric effect refers to the generation of electric charge in response to applied mechanical stress or strain, and is widely utilized for harvesting mechanical vibrations from the environment.^[^ ^168^, ^170^ ^]^ This effect arises in materials with non‐centrosymmetric molecular or crystal structures, where mechanical deformation leads to dipole reorientation and charge displacement, resulting in electrical output. Traditional piezoelectric materials for mechanical energy harvesting are generally divided into inorganic ceramics, such as lead zirconate titanate (PZT), barium titanate (BaTiO_3_), and zinc oxide (ZnO), and synthetic polymers like poly‐vinylidene fluoride (PVDF). Their excellent mechanoelectrical conversion capabilities have been widely demonstrated in harvesting energy from both natural sources (e.g., wind, waves, vibrations, and sound) and biological activities (e.g., organisms’ movements, respiration, and cardiac activity).^[^ ^169^, ^171^, ^172^, ^173^ ^]^ However, their inherent brittleness and rigidity, potential toxicity, poor biocompatibility, and inadequate responses have significantly hindered their practical applications, especially in wearable, implantable bioelectronics, where flexibility and biocompatibility are essential.

To address these limitations, researchers have turned to developing piezoelectric hydrogels, which integrate the piezoelectric effect with the softness, flexibility, and biocompatibility of hydrogels. In other words, piezoelectric hydrogels are flexible, biocompatible piezoelectric materials, and at the same time, they are self‐sustaining hydrogels capable of converting environmental vibrations into electricity. Their development not only enhances mechanical and physiological compatibility with biological tissues (e.g., skin, bone, and muscle) but also offers the potential to eliminate reliance on external power sources such as batteries, positioning piezoelectric hydrogels as promising candidates for applications in bioelectronics, tissue engineering, and wound healing.^[^ ^174^, ^175^, ^176^ ^]^

As discussed in Section 2, hydrogels are typically synthesized by physically or chemically crosslinking one or more monomers into a 3D network. The fabrication of piezoelectric hydrogels involves a similar crosslinking process. Depending on the role of the hydrogel matrix, piezoelectric hydrogels can be synthesized via two main approaches. In the first approach, the hydrogel itself possesses inherent piezoelectric properties, functioning both as the piezoelectric material and the polymeric matrix. In the second approach, external piezoelectric materials are incorporated into the hydrogel network, with the hydrogel primarily serving as a flexible, biocompatible host matrix.

Materials used to fabricate inherently piezoelectric hydrogels include natural polymers, such as gelatin, collagen, chitosan, proteins like silk fibroin, and polysaccharides such as cellulose and alginate. These biomaterials contribute to the excellent biocompatibility of the resulting piezoelectric hydrogels. However, their intrinsic piezoelectric coefficients are relatively low (typically ranging from 0.1 to 20 pC N^−1^) when compared to traditional piezoelectric materials such as BTO (60–190 pC N^−1^) and PVDF (20–30 pC N^−1^),^[^ ^177^ ^]^ which limits their effectiveness for high‐performance energy harvesting. Consequently, in most practical applications, these biomaterial‐based hydrogels are often reinforced with high‐performance piezoelectric fillers to enhance their output. This strategy aligns with the second synthetic approach, in which piezoelectric materials are incorporated into the hydrogel network.

**Figure 3:**
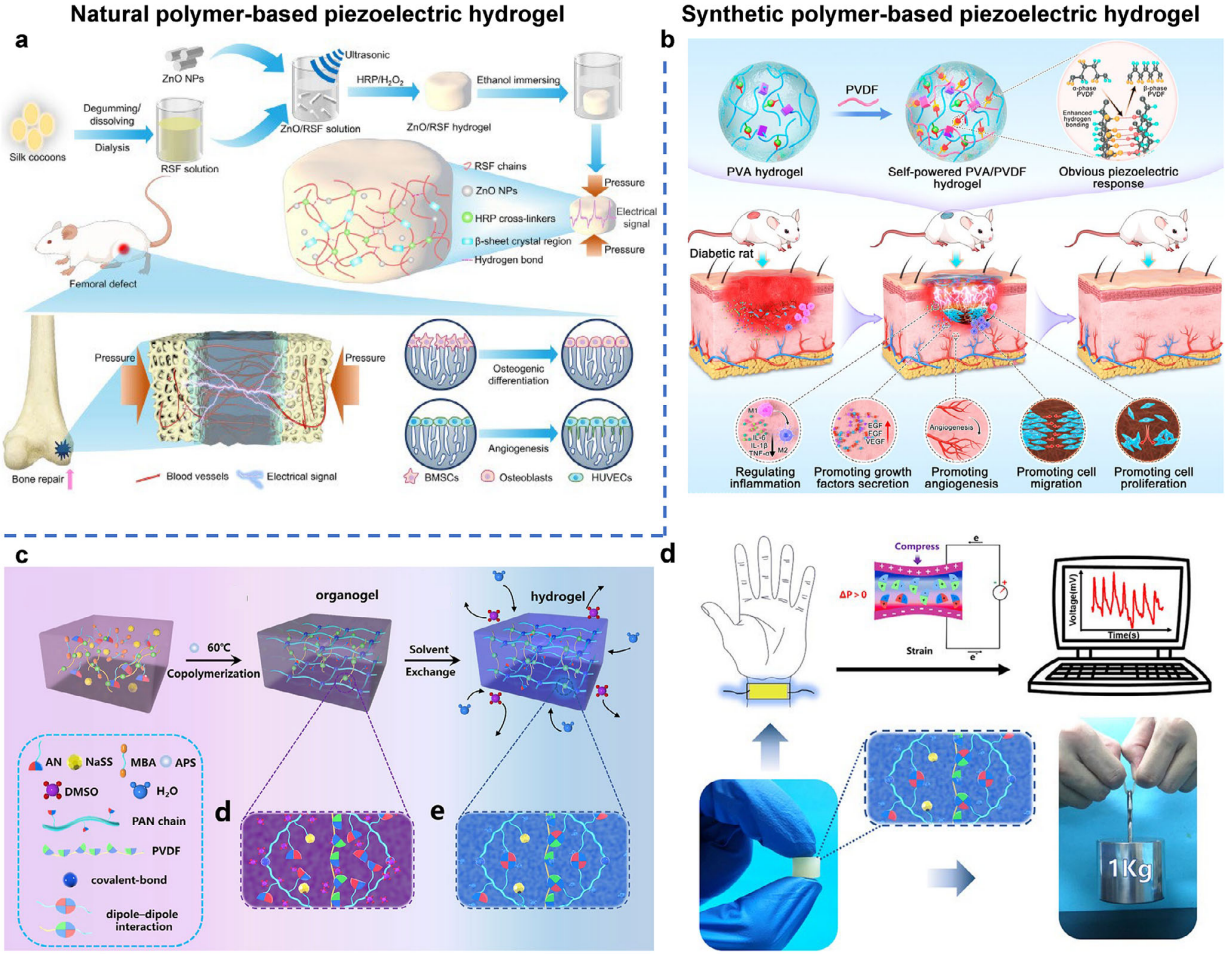
a) Schematic representation of ZnO‐RSF piezoelectric hydrogel bone regeneration. Reproduced with permission.^[^ ^178^ ^]^ Copyright 2025, Elsevier. b) Schematic diagram of the formation mechanism of a self‐powered piezoelectric PVA/PVDF composite hydrogel and its wound healing promotion mechanism in diabetic rats. Reproduced with permission.^[^ ^184^ ^]^ Copyright 2022, American Chemical Society. c) Formation of the PAN‐PVDF hydrogels. d) PAN‐PVDF hydrogel used as a sensor and demonstration of high mechanical strength. Reproduced with permission.^[^ ^185^ ^]^ Copyright 2019, American Chemical Society.

An example of such a biomaterial‐based, piezoelectric material‐filled piezoelectric hydrogel is shown in **Figure** **3**a. In this system, biocompatible zinc oxide (ZnO) nanoparticles are introduced into a solution of regenerated silk fibroin (RSF), and subsequently crosslinked through an enzyme‐initiated process to form a ZnO‐RSF piezoelectric hydrogel. The addition of ZnO significantly enhances the piezoelectric output: under a compressive stress of 0.1 MPa, the output voltage and current increased from 28 mV and 3 nA (in the ZnO‐free RSF hydrogel) to 83 mV and 45 nA with the inclusion of 0.6wt% ZnO. This improved piezoelectric performance enables the ZnO‐RSF hydrogel to function as a self‐powered sensor capable of detecting external mechanical stimuli such as arm bending and human walking. Furthermore, due to its excellent biocompatibility, the ZnO‐RSF hydrogel can be implanted into bone defect sites. When subjected to mechanical pressure, the hydrogel generates electric signals that promote the osteogenic differentiation of bone marrow mesenchymal stem cells (BMSCs) and enhance the vasculogenic ability of human umbilical vein endothelial cells (HUVECs), thereby facilitating bone tissue regeneration.^[^ ^178^ ^]^ Other examples include composite hydrogels made from BTO‐silk fibroin,^[^ ^179^ ^]^ BTO‐gelatin methacryloyl,^[^ ^180^ ^]^ PVDF‐TrFE‐chitosan,^[^ ^181^ ^]^ FeWO_4_‐chitosan,^[^ ^182^ ^]^ and PVDF‐cellulose.^[^ ^183^ ^]^

In addition to natural biomaterials, synthetic polymers, both piezoelectric and non‐piezoelectric, can also be used to fabricate piezoelectric hydrogels. For instance, a self‐powered piezoelectric hydrogel has been developed using piezoelectric PVDF and non‐piezoelectric poly(vinyl alcohol) (PVA).^[^ ^184^ ^]^ Through a multi‐step preparation process involving freezing/thawing, solvent replacement, annealing, and swelling, strong hydrogen bonding forms between PVA and PVDF. This interaction promotes the transformation of PVDF from its non‐polar α‐phase to the electroactive β‐phase, while also inducing a highly cross‐linked crystalline network. As a result, the hydrogel exhibits a high piezoelectric coefficient (8.4 pC N^−1^ at PVA/PVDF ratio of 7:3), and increased electric output (around 8.34 V and 261 nA). Furthermore, the composite hydrogel retains the hydrophilicity of PVA, allowing rapid absorption of wound exudate and maintaining a moist wound environment. This moisture retention, combined with the hydrogel's ability to generate localized electrical stimulation, regulates the proliferation, migration, and secretion of fibroblasts and vascular endothelial cells, thereby promoting wound healing (Figure 3b). Polyacrylonitrile (PAN) hydrogels exhibit notable toughness and piezoelectricity, primarily due to the interactions of cyano (CN) groups. When PVDF is incorporated into a PAN hydrogel, the highly polar CN groups interact with PVDF dipoles, significantly enhancing the formation of high electroactive β‐phase, from 0 to 91.3%. Consequently, the composite hydrogel achieves a high piezoelectric coefficient (30 pC N^−1^) and delivers an output of 30 mV and 2.8 µA.^[^ ^185^ ^]^ In addition to its piezoelectric performance, the PAN/PVDF hydrogel demonstrates a skin‐like Young's modulus (0.63‐1.80 MPa), stretchability (90‐175%), and high toughness (1.23 MJ m^−2^). These combined mechanical and electrical properties make it well‐suited for use in self‐powered pressure sensors capable of detecting physiological signals such as gesture, pulse, and words (Figure 3c,d). Other PAN‐based composite piezoelectric hydrogels have been reported as wound dressing for prophylaxis and early‐treatment of pressure injuries/pressure ulcers,^[^ ^186^ ^]^ or as flexible strain sensors to monitor a variety of body signals.^[^ ^117^ ^]^

#### 3.1.2 Piezoionic Energy Harvesting

It is well known that ions, instead of electrons in traditional electronics, dominate in many energy‐generation processes, such as in living matter and human‐machine interfaces.^[^ ^36^, ^187^ ^]^ Hydrogels are composed of a network of hydrophilic polymer chains immersed in water or other solvents, which confers hydrogels with high ionic conductivity. Moreover, hydrogels are often flexible, soft, and biocompatible, allowing them to generate a substantial deformation in response to even some minute physiological motions in a biological system. Consequently, using hydrogels to harvest mechanical energy has led to the emergence of a new technique in this field: Piezoionics. Piezoionics represents a novel approach that capitalizes on the synergistic interplay between mechanical strain and ionic movement within specially designed materials, particularly hydrogels.

**Figure 4:**
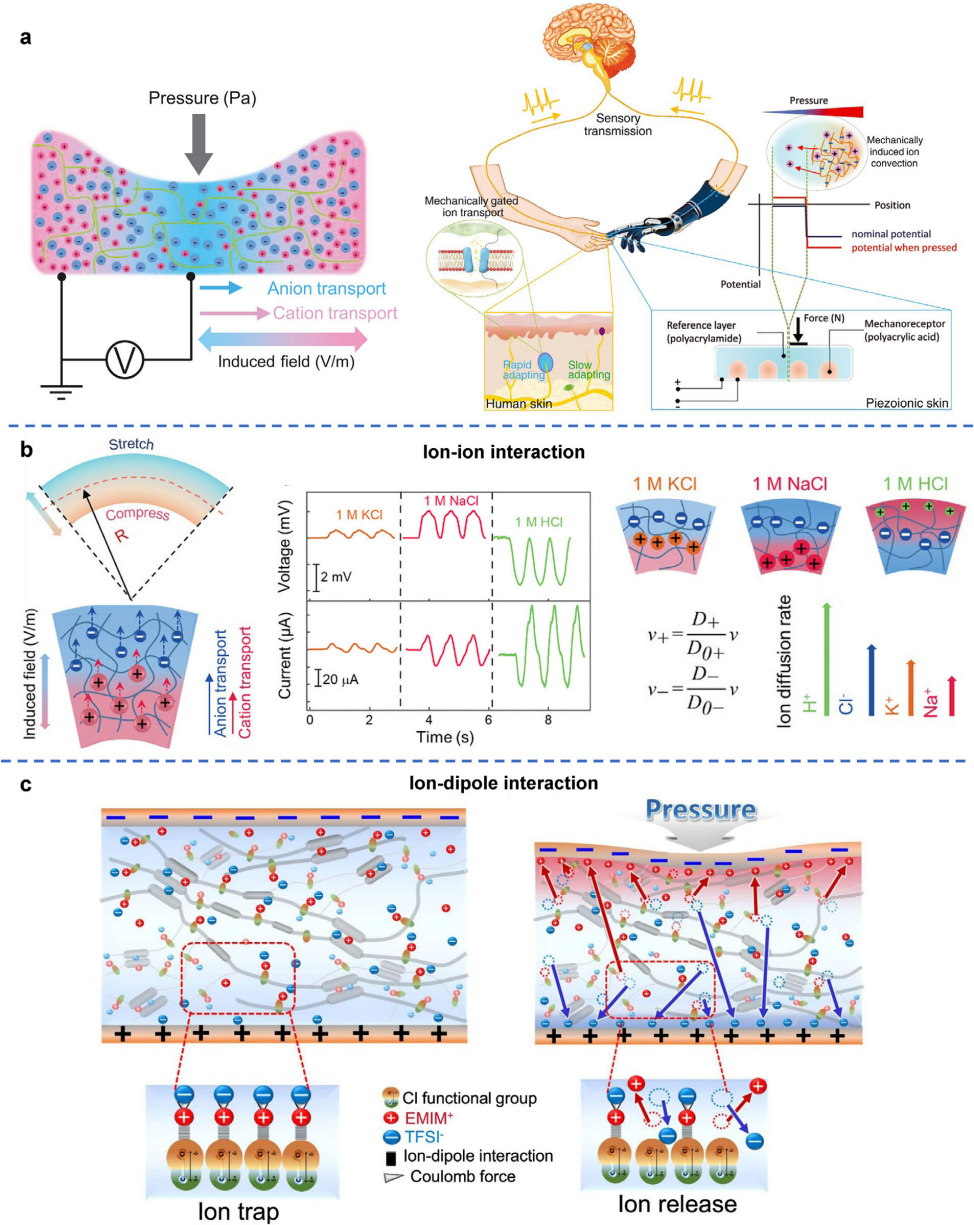
a) Schematic of a polymer gel under pressure, exhibiting differential ionic displacement and field, generating a charge imbalance and electric field. Schematic representation comparing biological sensory transduction and piezoionics. Reproduced with permission.^[^ ^56^ ^]^ Copyright 2020, AAAS. b) Voltage generation due to ionic transport in a bent hydrogel. The electrical output depends on the type of electrolyte used in swelling, and the difference in diffusion rates between the cations and anions. Reproduced with permission.^[^ ^191^ ^]^ Copyright 2024, Wiley‐VCH. c) Molecular design and working principle of the trapping and release of ions in response to mechanical stimuli due to ion‐dipole interactions. Reproduced under the terms of the CC‐BY Creative Commons Attribution 4.0 International license (https://creativecommons.org/licenses/by/4.0).^[^ ^192^ ^]^ Copyright 2022, The Authors, published by Springer Nature.

The piezoionic effect refers to the mechanical‐to‐electricity conversion process mediated by mechanical stress/strain‐induced separation of anions and cations within materials.^[^ ^56^, ^188^, ^189^ ^]^ As illustrated in **Figure** **4**a, when a hydrogel is subjected to external force or pressure, it undergoes deformation that creates a pressure gradient between the compressed and uncompressed area, driving the migration of ions toward the undeformed region. If the mobilities of cations and anions differ, this ion movement results in a transient charge separation, generating an electric current in the external circuit. This piezoionic phenomenon serves as a biomimetic analogue artificial analogy to mechanoreceptors in human skin, where mechanically gated ion transport underpins tactile sensory function.^[^ ^56^, ^190^ ^]^ In this case, ion transport plays a crucial role in energy harvesting, with enhanced ion conductivity and a greater mobility difference between positive and negative ions being key factors in boosting power generation.^[^ ^56^ ^]^ Tuning this mobility difference between cations and anions is an effective approach to control both the amplitude and polarity of the generated power. As demonstrated in Figure 4b, voltage arises from ionic transport within a bent hydrogel. The electrical outputs vary depending on the type of electrolyte used to swell the hydrogels. Specifically, the output amplitude is determined by the difference in diffusion rates between the cations and anions – the greater the mobility difference, the higher the voltage output. Furthermore, when the cation mobility surpasses that of the anions, the polarity of the electrical output switches from negative to positive.^[^ ^191^ ^]^ In addition to ion‐ion interactions, ion‐dipole interactions can also be employed to regulate the trapping and release of ions in response to mechanical stimuli. A representative example is a chlorine‐functionalized polyurethane (PU) matrix incorporating the ionic liquid (IL) (1‐ethyl‐3‐methylimidazolium bis(trifluoromethylsulfonyl)imide ([EMIM]+[TFSI]–)). In this system, ion‐dipole interactions form between the Cl groups in the PU matrix and the ion pairs of the IL. When mechanical pressure is applied, these ion‐dipole interactions are disrupted, leading to ion release. Upon release of the pressure, these interactions re‐form, resulting in the re‐trapping of ions (Figure 4c). This reversible mechanism offers a promising approach for mechanically responsive ion regulation in soft energy harvesting and sensing devices.^[^ ^192^ ^]^

Hydrogels employed for piezoionics can be either neutral polymeric networks infused with water or solvents containing ions, or polyelectrolytes with large amounts of fixed ions and mobile counterions. Force‐induced power generation has been observed in uncharged polyacrylamide (PAAm) hydrogels loaded with sodium chloride (NaCl) solution,^[^ ^56^ ^]^ as well as in polyelectrolyte polyacrylic acid (PAA) hydrogels.^[^ ^148^ ^]^ The power generation in the PAA hydrogel is driven by the difference in mobility between fixed acrylic acid groups and mobile protons. While in the case of NaCl‐embeded PAAm hydrogel, electricity production may arise from the polymeric matrix's differential hindrance on Na^+^ and Cl^−^ ions, leading to faster diffusion of Cl^−^ compared to Na^+^. As research in piezoionics continues to advance, promising avenues emerge for the development of innovative technologies using the mechanical energy present in our surroundings.

The fundamental difference between piezoelectric hydrogels and piezoionic hydrogels lies in their energy conversion mechanism. Piezoelectric hydrogels are based on dipole polarization, where the dipoles align under the applied force to generate charges. On the contrary, piezoionic hydrogels are based on ion migration, and the application of a mechanical force causes the ions to migrate and redistribute, generating power output. As a result of the difference in the energy conversion mechanism, piezoelectric hydrogels have a faster response time (milliseconds), while piezoionic hydrogels exhibit a slower response (milliseconds to seconds) due to the ion diffusion timescale. In terms of power output, piezoelectric hydrogels can produce tens to hundreds mV of voltage with a power density of µW cm^−1^ to mW cm^−1^, while piezoionic hydrogels generate lower voltages in the range of mV with a power density of nW cm^−1^ to µW cm^−1^. Based on the differences discussed above, piezoelectric hydrogels are more suitable for real‐time sensors and vibration energy harvesters, while piezoionic hydrogels are ideal where biocompatibility and ionic signaling are needed.

Another difference is that piezoionic hydrogels inherently offer ionic conductivity and mechanical softness, making them highly suitable for bio‐wearable or implantable devices. In comparison, piezoelectric fillers (especially inorganic ones) tend to be rigid and brittle, hence making piezoelectric hydrogels incompatible for applications that require soft and flexible energy harvesters. Moreover, as the hydrogels themselves are typically non‐piezoelectric, they need to undergo polarization, which involves complex processes such as electrical poling or mechanical stretching. However, solutions have already been developed to overcome such limitations, opening up great opportunities for piezoelectric hydrogels in bio‐wearable and implantable devices. For example, to resolve the issue of rigidity and brittleness, piezoelectric fillers that are nano‐sized can be used for integration with hydrogels, hence achieving soft and stretchable piezoelectric hydrogels. Another example is the potential of piezoelectric hydrogels for non‐contact stimulation, which is required in certain applications, such as the ultrasound‐activated piezoelectric hydrogels with antibacterial activity for wound healing.^[^ ^182^ ^]^

#### 3.1.3 Triboelectric Energy Harvesting

Triboelectric nanogenerators (TENGs), based on the conjunction of triboelectrification and electrostatic induction, have proven their high efficiency in converting ambient mechanical energy into electricity.^[^ ^11^, ^193^, ^194^ ^]^ Through materials engineering, especially with the introduction of hydrogels into their fabrication, TENGs can be designed to be flexible, stretchable, elastic, degradable, and biocompatible. A growing number of studies have highlighted the role of hydrogels as compliant components that enhance device flexibility and durability, thereby advancing the application of hydrogel‐based TENGs in soft electronics.^[^ ^78^, ^160^, ^195^, ^196^, ^197^, ^198^ ^]^ In these studies, hydrogels have primarily served two roles: as flexible electrodes and as active triboelectric materials.

**Figure 5:**
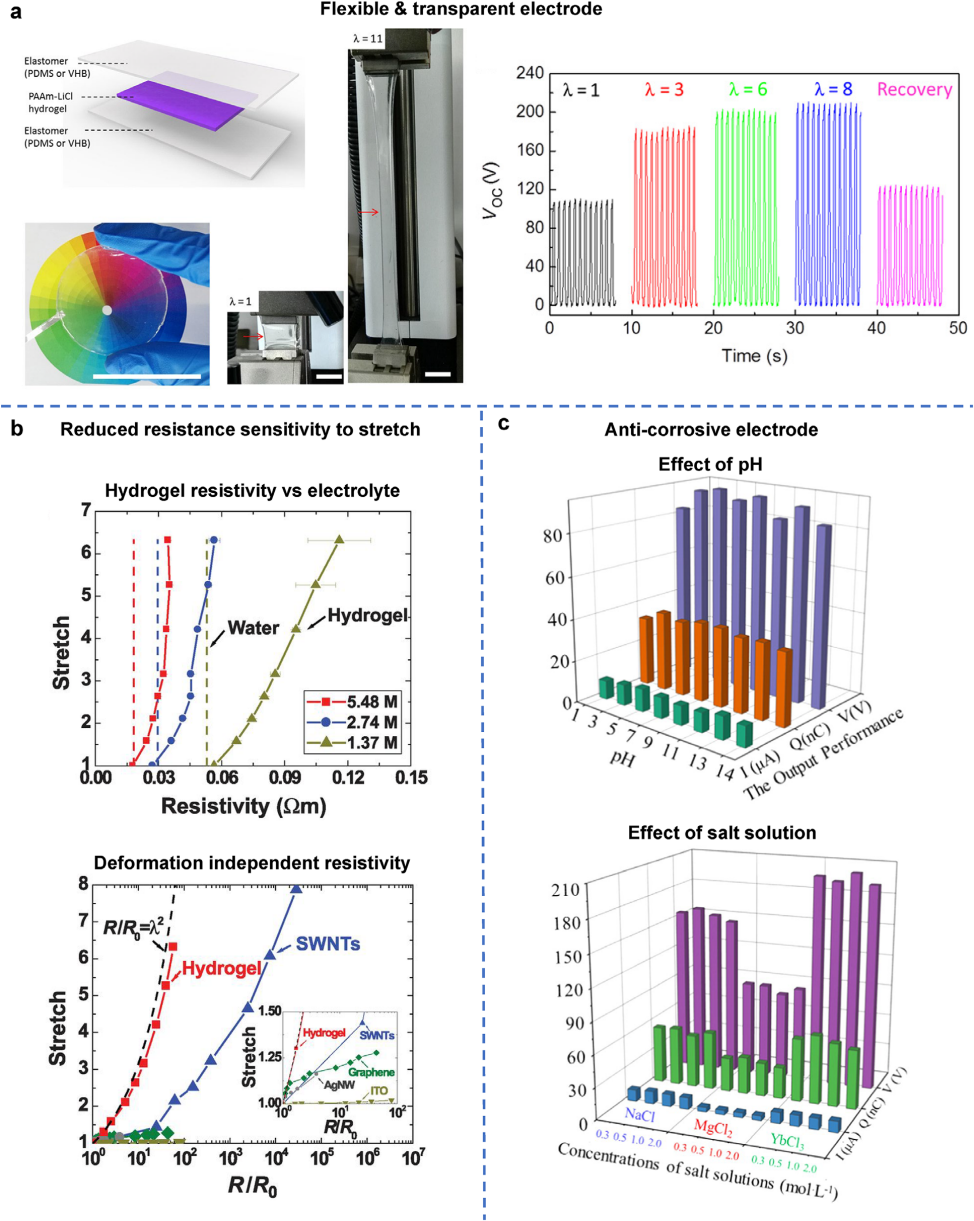
a) Scheme of the transparent TENG with sandwich structure. TENG (indicated by arrows) at initial state (stretch λ = 1) and stretched state (λ = 11 or strain ɛ = 1000%). Reproduced under the terms of the CC‐BY Creative Commons Attribution 4.0 International license (https://creativecommons.org/licenses/by/4.0).^[^ ^197^ ^]^ Copyright 2017, The Authors, published by AAAS. b) Electrical resistivities of hydrogels with various NaCl concentrations as a function of stretch (top), and normalized resistance plotted against stretch (bottom). Reproduced with permission.^[^ ^199^ ^]^ Copyright 2013, AAAS. c) Output performance of Cyc‐hydrogel as the electrode pretreated by aqueous solutions with different pH, and also pretreated by three kinds of inorganic salt solutions with different concentrations. Reproduced with permission.^[^ ^200^ ^]^ Copyright 2021, Elsevier.

Employing hydrogels as electrodes imparts unique properties to TENGs, including super‐stretchability, flexibility, structural robustness, strain‐resilient conductivity, environmental sustainability, and biocompatibility, among others. These attributes are difficult to achieve with conventional electrode materials, such as metal plates, or polymer composites infused with CNTs or Ag nanowires, posing challenges to developing a truly flexible power source. In 2017, Wang's group reported a TENG utilizing an ionic hydrogel film as the electrode, which was transparent and capable of stretching up to 1160%.^[^ ^197^ ^]^ Furthermore, as the TENG was stretched from its initial state to strains of 300% and 800%, the output voltage increased from 110 V to 180 V and 210 V, respectively, due to the enlarged contact area for electrification at flexibility‐enabled high strains (**Figure** **5**a).

Beyond enhancing TENG flexibility, hydrogel electrodes also reduce the resistance sensitivity to stretching deformation. Traditional flexible electrodes, such as CNT‐ or Ag nanowire‐percolated polymer electrodes, exhibit a significant increase in resistance under strain due to the elongation of the electron transfer path or the loss of percolation at large deformations. This sharp resistance increase degrades TENG performance. In contrast, hydrogels demonstrate lower resistance sensitivity to stretching, particularly when modified as ionic conductors, for example, through salt solution infusion. Suo's group observed that the resistivity of un‐stretched hydrogels depends primarily on the salt concentration within the polymeric network (Figure 5b, top). As the hydrogel stretches, its resistivity increases in a deformation‐independent manner, following a square law relationship that is significantly slower than that of CNTs, Ag nanowires, or graphene. Consequently, the increase in hydrogel resistivity was found to be orders of magnitude lower than that of electronic conductors under strain (Figure 5b, bottom).^[^ ^199^ ^]^ Using hydrogels as the TENG electrodes can effectively mitigate resistance increases and associated performance degradation.^[^ ^197^ ^]^

Moreover, hydrogels have emerged as a promising alternative to traditional electrodes, which often degrade in harsh environments, particularly when exposed to biological fluids. Through a self‐polymerization reaction, an anticorrosive, chemically robust, and durable hydrogel incorporating β‐cyclodextrin (β‐CD) molecules, termed Cyc‐hydrogel, has been developed. As an electrode material for TENGs, this Cyc‐hydrogel ensures not only high TENG performance, but also stable power output even under extreme conditions, including highly acidic, alkaline, or saline conditions (Figure 5c).^[^ ^200^ ^]^

Beyond their role as flexible electrodes, hydrogels have also attracted interest as active triboelectric materials, expanding their functional scope in TENG devices. It has been demonstrated that electricity can be generated by mechanically modulating electrical double layers (EDLs) at the interface between a liquid bridge and two conductive or dielectric plates. When subjected to mechanical vibrations, the EDL areas and capacitance at these interfaces undergo periodic variations, resulting in the output of alternative current in the external circuit.^[^ ^201^, ^202^ ^]^ Wang's group later described the formation of EDLs as a two‐step process: first contact electrification (or electron transfer) at the liquid‐solid interface, followed by electrostatic counterion adsorption.^[^ ^203^ ^]^ Despite extensive studies on triboelectricity at liquid–solid interfaces,^[^ ^204^, ^205^, ^206^ ^]^ practical applications of the liquid‐based TENGs for power generation face inherent challenges, including liquid leakage, evaporation, and narrow working bandwidth due to water bridge deformation under high‐frequency vibration.

**Figure 6:**
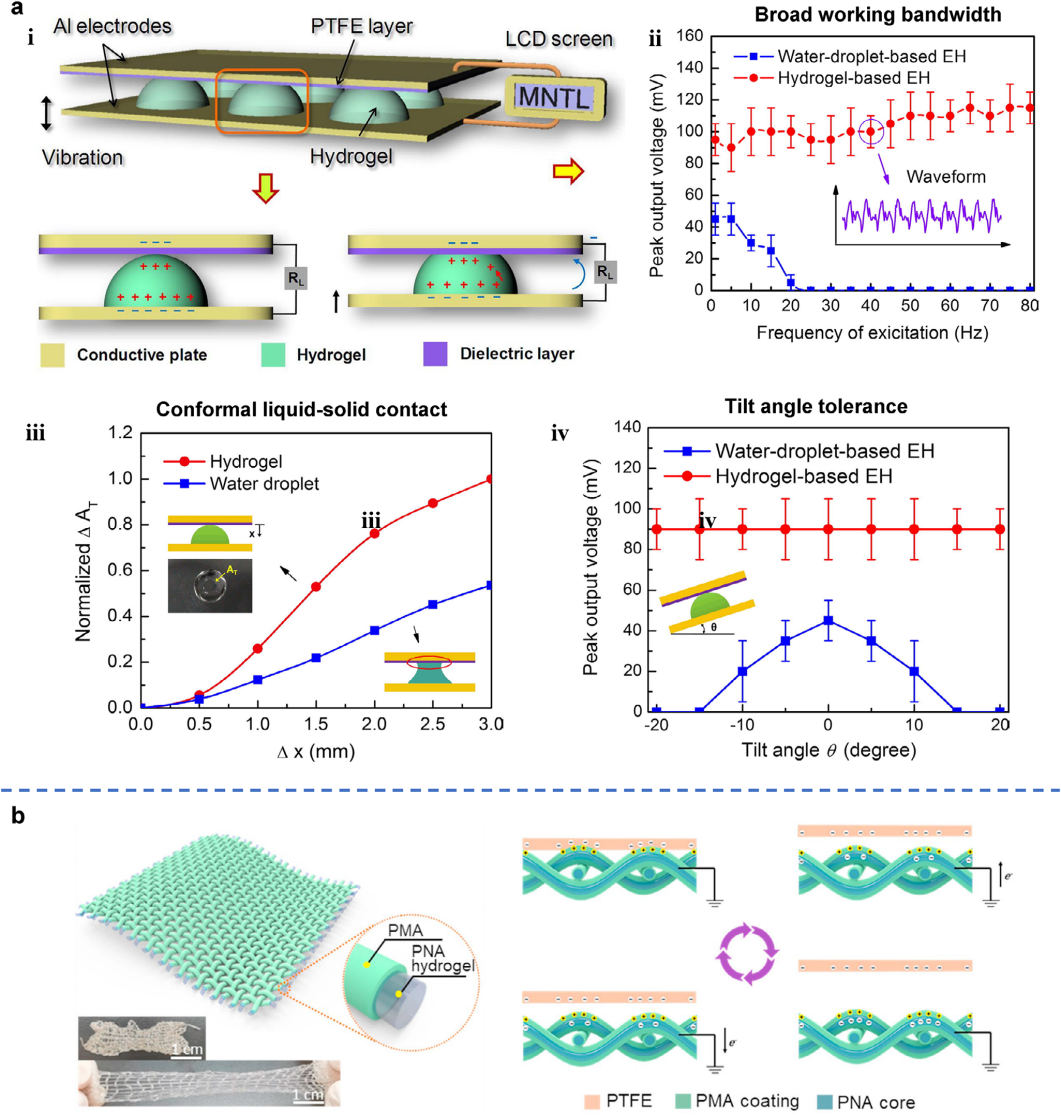
a, i) Working principle of hydrogel‐based TENG. ii) Bandwidth and output performance comparison between water‐droplet‐based energy harvester (EH) and hydrogel‐based EH. iii) Contact area change comparison between the two types of energy harvesters. iv) Tilt angle tolerance characterization for the two types of energy harvesters. Reproduced with permission.^[^ ^198^ ^]^ Copyright 2016, Elsevier. b) Schematic illustration of a woven TENG textile in contact‐separation single‐electrode mode. Reproduced with permission.^[^ ^76^ ^]^ Copyright 2020, Elsevier.

To overcome these issues, hydrogels have proven effective due to their extraordinary ability to retain large amounts of water in a solid‐like network, significantly reducing leakage and deformation concerns. More importantly, beyond preventing leakage and deformation, hydrogels provide an ideal liquid‐solid interface, as their flexibility enables conformal deformation under mechanical vibrations, ensuring a substantial liquid‐solid contact area (**Figure** **6**a(iii)). As a result, a hydrogel‐based TENG, in which hydrogels replace water droplets (Figure 6a(i)), achieved a broad working bandwidth of 0–80 Hz when sandwiched between a dielectric layer and a metal electrode (Figure 6a(ii)). This hydrogel‐based TENG also exhibits high working reliability and a large tolerance of tilt angle during installation (Figure 6a(iv)).^[^ ^198^ ^]^ Similarly, triboelectricity generation has been realized at the interface between a flexible “solid water” (hydrogel) and a soft solid (dielectric elastomer), which has been used as a self‐powered sensor capable of detecting subtle human activities, such as throat movements, vocal cord vibrations, muscle motions, and abdominal breathing.^[^ ^207^ ^]^ Beyond film‐based applications, hydrogels can also be fabricated as fibers for smart textiles to harvest triboelectric energy. Using a dry‐to‐wet spinning method, hydrogel fibers have been produced (Figure 6b), exhibiting tensile strength of 2.27 MPa, stretchability of 900%, high conductivity of 0.69 S m^−1^, and self‐healing properties. These hydrogel fibers can be woven into TENG textiles, achieving an output voltage of up to 36 V. Importantly, they exhibit superior power generation “elasticity,” with the voltage recovering to its initial value after releasing the strain.^[^ ^76^ ^]^

Beyond contributing to flexible electrodes and active materials, introducing hydrogels into a TENG also brings several fascinating features owing to their versatility and tunable properties. These enhancements include improved interfacial stability, biodegradability, and even additional energy generation mechanisms. For instance, interfacial modification with benzophenone (BP) enables strong bonding between the hydrophilic hydrogel (electrode) and the hydrophobic elastomer (triboelectric film), significantly enhancing structural stability during stretching.^[^ ^196^ ^]^ Furthermore, the use of biodegradable polyvinyl alcohol (PVA) hydrogel in TENGs provides high flexibility, recyclability, and environmental sustainability.^[^ ^160^ ^]^ Incorporating 2D Mxene nanosheets into PVA hydrogel further improves stretchability by strengthening the cross‐linking of the polymeric chains. Simultaneously, these Mxene nanosheets create microchannels on the hydrogel surface, facilitating ion transport and thus enhancing conductivity. Interestingly, these microchannels also enable a supplementary energy generation mechanism, a streaming vibration potential, that further boosts power output.^[^ ^78^ ^]^

### 3.2 Conversion of Thermal Energy (Temperature Gradients)

Thermal energy, especially low‐grade heat, is abundant and ubiquitous in our environment, arising from natural sources such as geothermal or solar energy, as well as from a variety of industrial activities and physiological processes within organisms.^[^ ^208^, ^209^ ^]^ However, most of the low‐grade heat remains untapped and is rarely used commercially, given its inherent low energy efficiency. In the context of increasingly urgent energy demands, it is crucial to develop technologies that can convert low‐grade heat into electricity in an efficient, economic, and scalable manner. Thermoelectric (TE) technology, operating on the “Seebeck effect” (the generation of a voltage across a material when subjected to a temperature gradient), offers a simple and environmentally friendly solution for direct heat‐to‐electricity conversion without moving parts, audible noise, or greenhouse gas emissions.^[^ ^210^, ^211^ ^]^ The performance of thermoelectric devices is often evaluated using an intrinsic material parameter, the thermoelectric figure of merit (ZT), *ZT*  =  (*S* ^2^σ/κ)*T*, where *T* is the temperature, *S* is the Seebeck coefficient, σ and κ represent the electrical conductivity and thermal conductivity, respectively.^[^ ^211^ ^]^ Accordingly, enhancing TE performance involves strategies aimed at maximizing the *S* ^2^σ (also defined as the power factor (PF)), and/or minimizing thermal conductivity. According to the charge carriers responsible for the thermal voltage generation, the thermoelectric materials can be classified as traditional TEs and ionic TEs. Traditional TEs, also known as e‐TEs, rely on the thermodiffusion of electronic charges (electrons for n‐type e‐TEs and holes for p‐type e‐TEs) to deliver thermopower under a temperature gradient. Instead of electronic charges, ions account for the power generation in ionic TEs (or i‐TEs). Introducing hydrogels into these systems can address issues associated with both traditional and ionic TEs, and also usher in unexpected features like ultra‐flexibility, self‐healing capabilities, high tunability in properties and performance, as well as environmental adaptability. A summary of relevant works is presented in **Table** **3** at the end of this sub‐section.

**Table 3:** Summary of hydrogels for natural and biological environmental energy harvesting—Temperature gradient.

Mechanisms	Key roles played by hydrogels	Hydrogels' properties required	Typical Hydrogels: Synthesis and Performance	Applications	Refs.
**Thermoelectric**	Synergize inorganic & organic TEs Host electrolytes Stabilize environment RH	Flexibility in modification High hydration, “solid liquid” High hydration, hygroscopic	Te‐NWs‐PEDOT:PSS/PVA hydrogel, freeze‐thaw gelation Stretchability: 400% Energy output: 787 µV K^−1^. 23.8 mV, 0.83 mA, 33.7 µW m^−2^ at ΔT = 30 K	Energy generator	[214]
			CuyTe – AgxTe nanorods/Carboxylated cellulose nanofibers, 3D printed Energy output: 360.5 mV, 1.278 W m^−2^ (70 pairs of TE legs), at ΔT = 50 K	Energy generator	[215]
			Ionic liquid‐doped PEDOT:PSS glycerol hydrogel, Energy output: 5 mV, 30 mW m^−2^ (12 pairs of TE legs), at ΔT = 30 K		[217]
			Poly(styrene sulfonic acid)‐doped PEDOT:PSS with MOF‐801 hydrogel layer hydrogel Energy output: 16.2 mV K^−1^. 7.6 mW m^−1^ K^−2^. 81 mV, 1.43 mA cm^−2^, 33.7 mW m^−2^ at ΔT = 5 K		[218]
**Ionic thermoelectric**	Host electrolytes Flexible, wearable, conformable Ionic selectivity	high hydration, “solid liquid” Soft, flexible, stretchable, biocompatible Able to regulate ionic diffusion	NaOH‐Cellulose‐Poly(ethylene oxide (PEO) hydrogel Energy output: 24 mV K^−1^		[222]
			Gelatin with [Fe(CN)_6_]^4−^/^3−^ redox couple, Energy output: p‐type, 17.0 mV K^−1^. 2 V and 5 mW using body heat (25 sets)		[223]
			Methylcellulose with KCl (I^−^/I^3−^ redox couple) Energy output: – 8.18 mV K^−1^ (n‐type), 9.62 mV K^−1^ (p‐type), at ΔT = 15 K		[227]
			PAAm hydrogel with Li^+^, Br^–^, K^+^, and [Fe(CN)_6_]^4−^/^3‐^ redox couple, Energy output: 1.2 mV K^−1^. 24 mV, 0.48 A m^−2^, 2.8 mW m^−2^ at ΔT = 20 K	Low‐grade heat harvesting	[163]
			Polyacrylamide (PAAm) /LiCl ionic hydrogel Energy output: 11.3 mV K^−1^. 280 mV, 1.62 A m^−2^, 167.90 mW m^−2^ at ΔT = 20 K		[233]
			Polyacrylamide (PAAm) hydrogel with (Fe^2+^/Fe^3+^ and [Fe(CN)_6_]^4−^/^3−^) redox couples Energy output: ‐1.05 mV K^−1^. 1.245 mV K^−1^.		[27]
			Polyacrylamide (PAAm), calcium‐alginate hydrogel Elongation: 2800% Energy output: 11.5 mV K^−1^. 450 mV, 0.84 A m^−2^, 94.38 mW m^−2^ at ΔT = 20 K		[234]
			Bacterial cellulose (BC) hydrogel with urea and NaCl and [Fe(CN)_6_]^4−^/^3−^ redox couple Energy output: 1.52 mV K^−1^. 14 mV, 7.87 A m^−2^, 22.5 mW m^−2^, at ΔT = 10 K		[235]
			Bacterial cellulose (BC) hydrogel with (Fe^2+^/Fe^3+^ and [Fe(CN)_6_]^4−^/^3−^) redox couples Energy output: ‐4.5 mV K^−1^ (n‐type), +0.72 mV K^−1^ (p‐type). 0.82 V, 23 µA, 4.5 µW (3 p‐n pairs at ΔT = 20 K):		[236]
			Polyquaternium‐10 (PQ‐10) hydrogel with NaOH Energy output: 24.17 mV K^−1^		[237]
			Bacterial cellulose (BC) hydrogel with ([EMIm][DCA] Tensile strength: 3.05 MPa, stretchability: 40.99%, adhesivity, “green” solvent Energy output: 18.04 mV K^−1^		[238]
			Poly(vinylidene fluoride‐co‐hexafluoropropylene) with EMIMCl or LiBF_4_ Stretchability: 1700%, Young's modulus: 0.26 MPa. Energy output: −15 mV K^−1^ (n‐type), +19 mV K^−1^ (p‐type). 0.5 V, 10 pairs at ΔT = 2 K		[239]
			PVDF‐HFP hydrogel with [EMIM]) ([TFSI]) Energy output: Seebeck coefficient: ‐4 mV K^−1^ (n‐type), +13 mV K^−1^ (p‐type with PEG)		[240]
			PVDF‐HFP hydrogel with [EMIM] [TFSI] with LiBF_4_ or EMIMCl Energy output: ‐15 mV K^−1^ (n‐type), +17 mV K^−1^ (p‐type with PEG)		[241]
			Pam‐alginate hydrogel containing EmimBF_4_ and PEG Energy output: 19.32 mV K^−1^, 0.31 µW cm^−2^		[165]
			(PAA‐PEO‐NaCl) ionic hydrogel Breaking stress > 1.3 MPa, stretchability > 1100%, toughness: 7.34 MJ m^−3^ Energy output: 3.26 mV K^−1^		[242]
			[PVDF‐HFP/NaTFSI/PC (PhNP)] Energy output: ‐6 mV K^−1^ (n‐type), +20 mV K^−1^ (p‐type)		[219]
			PVA hydrogel with guanidinium chloride and [Fe(CN)_6_]^4−^/^3−^] redox couple Tensile strength: 19 MPa, stretchability: 1300%, toughness of 163.4 MJ m^−3^ Energy output: 6.5 mV K^−1^. 160 mV, 43.0 A m^−2^, 1.725 W m^−2^, at ΔT = 30 K		[244]
			PAAm‐SA hydrogel with guanidine hydrochloride and [Fe(CN)_6_]^4−^/^3−^ redox couple Energy output: 4.4 mV K^−1^. 214.5 mV, 82.3 A m^−2^, 4.38 W m^−2^ at ΔT = 50 K		[245]
			PAM‐CMC network hydrogel with Li2_S_O_4_ and [Fe(CN)_6_]^4−^/^3‐^ redox couple Elongation: 634% Energy output: 11.58 mV K‐1. 15.1 mW m‐2 at ΔT = 10 K		[246]

#### 3.2.1 Thermoelectric Energy Harvesting

Many traditional thermoelectric materials, including inorganic semiconductors and organic conductive polymers, have been extensively studied for heat‐to‐electricity conversion.^[^ ^212^, ^213^ ^]^ However, these traditional TE devices still face several intrinsic challenges: 1) low heat‐to‐electricity conversion efficiency, partly due to the difficulty in reconciling the power factor (PF) with thermal conductivity (κ), 2) high production and/or material cost, 3) complex and demanding fabrication, and 4) mechanical properties that are incompatible for flexible, wearable, or bio‐electronic applications.

**Figure 7:**
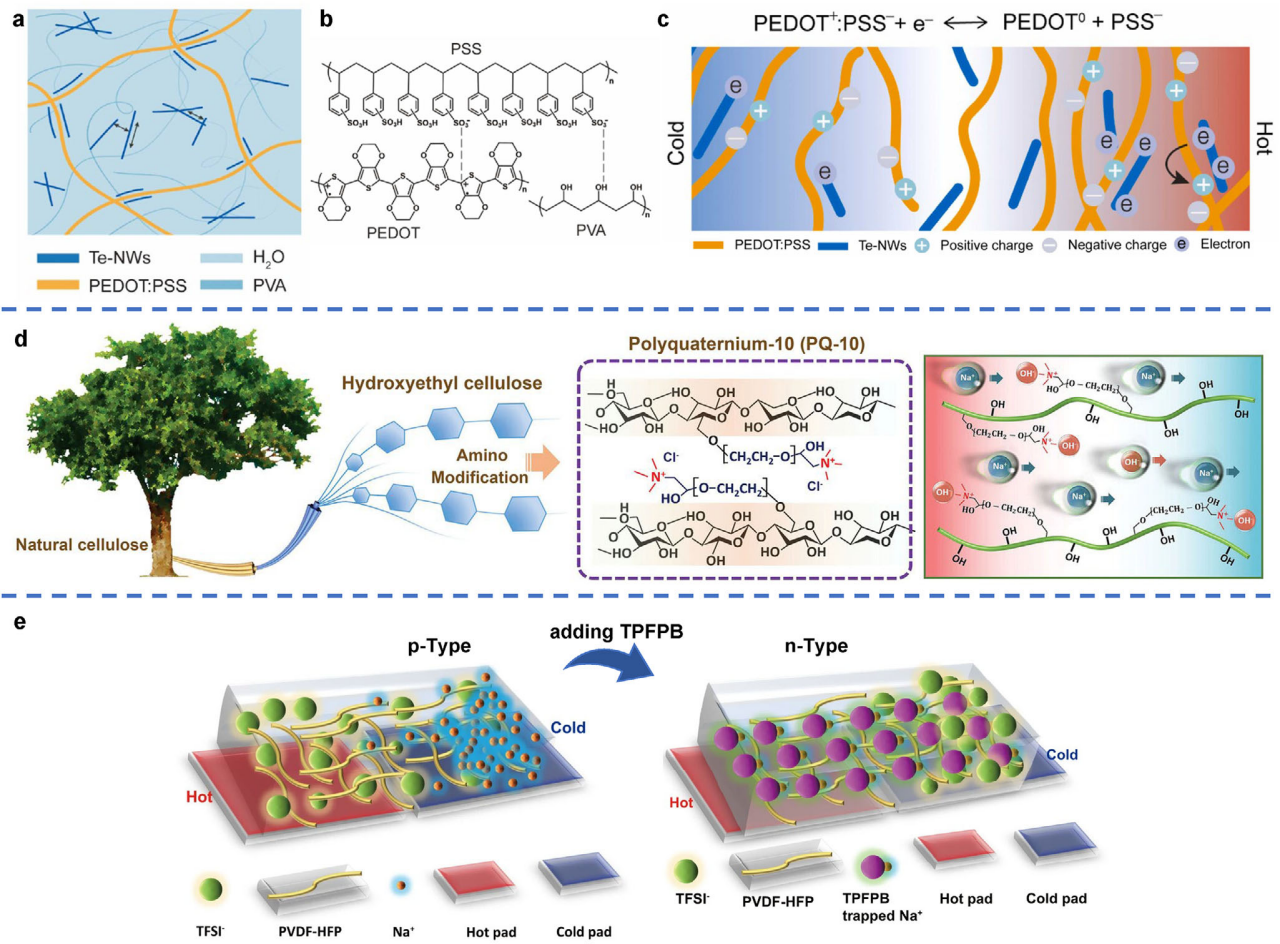
a) Structure of TPP hydrogels. b) Internal intermolecular interaction. c) Schematic diagram of the working principle of the TPP hydrogel. Reproduced with permission.^[^ ^214^ ^]^ Copyright 2023, Elsevier. d) Chemical structure of PQ‐10 derived from natural cellulose. Selective migration of Na^+^ cations in the i‐TE hydrogel under a temperature gradient. Reproduced with permission.^[^ ^237^ ^]^ Copyright 2023, Wiley‐VCH. e) Illustration of the p‐type (Na^+^ cations dominate thermodiffusion) and n‐type (TFSI−anions dominate thermodiffusion) i‐TE materials. Reproduced under the terms of the CC‐BY Creative Commons Attribution 4.0 International license (https://creativecommons.org/licenses/by/4.0).^[^ ^219^ ^]^ Copyright 2022, The Authors, published by Springer Nature.

The integration of hydrogels offers a promising approach to overcome these limitations by serving as a multifunctional matrix capable of coordinating and synergizing the properties of diverse TE components. For example, a polyvinyl alcohol (PVA) hydrogel framework has been employed to host inorganic thermoelectric tellurium nanowires (Te‐NWs) and organic thermoelectric conductive polymer PEDOT:PSS, termed as TPP hydrogel in **Figure** **7**a. In this TPP composite hydrogel, each material plays a complementary role: Te‐NWs enhance the inherently low thermoelectric output of PEDOT:PSS, the organic component reduces the high thermal conductivity of the inorganic phase, and the 3D soft, porous structure of the PVA hydrogel imparts excellent mechanical flexibility. Specifically, as shown in Figure 7b, PEDOT^+^ and PSS^−^ are initially bound by electrostatic attraction. After introducing the PVA hydrogel, the sulfonate acid groups in PSS are inclined to form hydrogen bonds with the hydroxyl groups in PVA. In this case, three types of interactions, including the polymer chain entanglement, electrostatic attraction, and hydrogen bonding, are coupled in the TPP hydrogel, resulting in superior mechanical properties compared to both pure inorganic thermoelectric hydrogel (TP) and organic thermoelectric hydrogel (PP) (Figure 7c). When this TPP hydrogel is applied with a temperature gradient for thermoelectric conversion, electrons accumulate more on the hot side than on the cold side, since both Te and PEDOT are p‐type thermoelectric materials. The electron accumulation on the hot side could trigger a thermal‐electrochemical process, converting positive PEDOT^+^ to neutral PEDOT^0^ and generating more PSS^−^ on the hot side. By synergizing the carrier diffusion and thermal‐electrochemical reaction, the Seebeck coefficient of TPP hydrogels can be significantly improved compared to both inorganic and organic thermoelectric materials. As a result, this hydrogel e‐TE module achieves a high Seebeck coefficient of 787 µV K^−1^, low thermal conductivity of 0.468 W m^−1^ K^−1^, and high stretchability of 400%.^[^ ^214^ ^]^

Inorganic TE nanomaterials can also be assembled into flexible TE devices by printing. Typically, high‐viscosity organics are added to facilitate the assembly of TE nanomaterials, but these additives often degrade the electrical conductivity and overall TE performance. Replacing such organics with a hydrogel made from cellulose nanofibers can significantly mitigate these drawbacks. The improvement is attributed to the hydrogel networks, formed by physical crosslinking and molecular entanglement, which can effectively limit the fluidity of 1D nanorod dispersion, even with cellulose nanofiber content as low as 0.498 wt%.^[^ ^215^ ^]^

Organic conductive polymers are attractive for flexible TE applications due to their inherent softness and processability, but their TE performance is usually limited by low electrical conductivity. When these polymers are converted into hydrogel form, they can be infused with organic solvents or highly conductive ionic liquids (ILs), enhancing conductivity while retaining flexibility, biocompatibility, deformability, and biodegradability. For example, PEDOT:PSS fibers can be gelled into a hydrogel matrix, allowing the introduction of organic solvents such as ethylene glycol and dimethyl sulfoxide. This solvent‐infused PEDOT:PSS hydrogel demonstrates a threefold increase in electrical conductivity along with an enhanced thermoelectric power factor.^[^ ^216^ ^]^ Similarly, incorporating ionic liquid (IL) into PEDOT:PSS hydrogels induces ion exchange between IL and PEDOT:PSS, followed by nano‐phase segregation of PEDOT chains. This microstructure alteration results in a conductivity of up to 305 S cm^−1^, eight times higher than that of the filler‐free hydrogels, significantly boosting the areal output power of the TE device. Remarkably, these hydrogels also exhibit fast self‐healing, and shape/size‐tunable characteristics, which are highly desirable for next‐generation wearable and bioelectronic applications.^[^ ^217^ ^]^

In most traditional thermoelectric materials, electrons or holes serve as the primary charge carriers for the Seebeck effect. However, ionic doping has emerged as a strategy to introduce ions as additional charge carriers, giving rise to electronic‐ionic‐mixed TE materials with enhanced PF, such as the proton‐doped PEDOT:PSS. Notably, the output performance of these electronic‐ionic‐mixed TE harvesters is highly sensitive to humidity, often showing strong variations with ambient moisture. Hydrogels, with their capacity to retain high water content, are well‐suited to maintain a stable local humidity environment. By incorporating a hydrogel‐based self‐humidifying (SH) layer into a proton‐doped PEDOT:PSS film, a robust TE harvester is developed. In this TE device, the SH layer provides a consistent 90% relative humidity (RH) around the active TE material, leading to stable power output under ambient conditions.^[^ ^218^ ^]^

#### 3.2.2 Ionic Thermoelectric Energy Harvesting

Despite extensive studies on traditional TE, the low thermopower (≈100–200 µV K^−1^),^[^ ^219^ ^]^ stemming from the strong interdependence of the Seebeck coefficient (*S*), electrical conductivity (σ), and thermal conductivity (κ), has encouraged explorations for new technologies. An alternative is the emerging class of ionic thermoelectric materials, which, through the ionic dynamics under a temperature gradient, can generate thermopower on the order of tens of mV K^−1^,^[^ ^220^, ^221^, ^222^, ^223^, ^224^, ^225^ ^]^ more than one order of magnitude higher than that of traditional TE materials. The thermopower generation of ionic TEs involves two mechanisms, i.e., the thermodiffusion effect (or the Soret effect) and the thermogalvanic effect (or the thermoelectrochemical effect). The thermodiffusion effect refers to thermal voltage generation through the selective migration of ions under a temperature gradient,^[^ ^226^ ^]^ while the thermogalvanic effect relies on the temperature‐dependent redox reactions at two electrodes.^[^ ^220^ ^]^ Typically, an ionic TE device features a liquid‐state electrolyte containing ions with different mobilities (for thermodiffusion) or redox couples (for thermo‐galvanic).^[^ ^220^, ^221^, ^227^, ^228^, ^229^, ^230^ ^]^

The use of liquid‐state electrolytes not only poses leakage risks that complicate the integration and packaging of TE devices, but also limits the application of ionic TE in wearable electronics or bioelectronics. However, by introducing hydrogels into ionic TE, or by gelling the liquid electrolyte, a quasi‐solid or solid ionic TE can be created to circumvent the leakage issue. More interestingly, beyond merely solidifying the electrolyte, these hydrogels also play multiple roles in heat‐to‐electricity conversion. For instance, they can optimize the magnitude and tune the nature of the thermopower output, enhance the mechanical toughness, flexibility, and stretchability to facilitate practical applications of ionic TEs. Additionally, hydrogels can impart new features like environmental adaptability, while also easing and “greening” the manufacturing processes.

To address the leakage risk of liquid electrolytes, agarose and beads of poly(sodium acrylate) were explored as the gelling agents to solidify the electrolyte containing the redox pair of Fe(CN)_6_^4−^/Fe(CN)_6_^3−^.^[^ ^231^ ^]^ The resulting gelled electrolyte maintains electrocatalytic effects similar to the ungelled liquid electrolyte. And, as the gelled electrolyte can effectively impede heat transfer through the cell, the gelled electrolyte‐based thermocell demonstrates a more stable thermopower output compared to the ungelled electrolyte. Likewise, poly(vinyl alcohol), PVA was used to gel electrolytes containing different redox pairs to develop n‐type or p‐type ionic TEs. These hydrogel ionic TEs demonstrate thermoelectric performance and good mechanical properties, enabling the construction of a flexible and wearable energy harvester capable of generating output voltage up to 1 V from body heat.^[^ ^232^ ^]^ Besides agarose and PVA, a variety of polymeric gels have been employed to solidify electrolytes, producing solid or quasi‐solid ionic TEs that are safer and more user‐friendly than their liquid counterparts. These polymeric gels include gelatin,^[^ ^223^ ^]^ poly(acrylamide) (PAAm),^[^ ^27^, ^163^, ^233^, ^234^ ^]^ cellulose hydrogels,^[^ ^235^, ^236^, ^237^, ^238^ ^]^ poly(vinylidene fluoride‐co‐hexafluoropropylene) (PVDF‐HFP),^[^ ^239^, ^240^, ^241^ ^]^ among others.

As discussed earlier, the low thermal conductivity of the electrolyte hydrogel contributes to a more stable thermopower output compared to liquid electrolytes.^[^ ^231^ ^]^ Recent advancements in ionic TEs have witnessed the growing importance and versatility of hydrogels in enhancing and modulating thermopower. In the thermodiffusion‐based thermocell, a key strategy for achieving a high thermopower is to create a large difference in thermal mobility of cations and anions. This selective ionic mobility can be attained within hydrogel networks by precisely tuning ion–ion^[^ ^165^, ^237^, ^242^ ^]^ or ion–dipole^[^ ^239^, ^240^ ^]^ interactions between the polymer matrices and the ionic species. For example, the ion‐ion interaction between the hydrogel matrix and both Na^+^ and OH^−^ plays a central role in the enhanced thermopower of an ionic TE system based on a cellulose hydrogel with sodium hydroxide (NaOH) as the ion source. In this system, the cellulose hydrogel matrix, which becomes positively charged when exposed to water, facilitates the thermodiffusion of Na^+^ under a temperature gradient, while the mobility of OH^−^ is strongly suppressed due to electrostatic attraction with the positively charged polymeric network (Figure 6d).^[^ ^237^ ^]^ This differential mobility leads to an elevated Seebeck coefficient.

Similar ionic selectivity can also be achieved using other polyelectrolyte gel networks, such as polyacrylic acid and poly(ethylene oxide), which have demonstrated comparable enhancements in thermal voltage generation. ^[^ ^165^, ^242^ ^]^ The ion‐dipole interaction, on the other hand, often occurs between ion sources and polymers containing strong electron‐withdrawing groups. For example, poly(vinylidene fluoride) (PVDF), with its highly electronegative fluorine atoms, readily forms ion‐dipole interactions when combined with ionic liquids (ILs). These interactions promote the formation of PVDF's β‐phase, ^[^ ^243^ ^]^ which is rich in polar dipoles and highly responsive to ionic motions. By varying the composition of polyelectrolytes or doping ions in the IL/PVDF gels, it is possible to modulate the strength and nature of ion‐dipole interactions. This tunability enables significant thermopower variation—from −4 mV K^−1^ to +14 mV K^−1^—as well as conversions between n‐type and p‐type TEs.^[^ ^219^, ^240^, ^241^ ^]^ Figure 6e illustrates the transformation of an original p‐type ionic thermoelectric material into an n‐type i‐TE by modulating ion transportation through the incorporation of tris(pentafluorophenyl)borane (TPFPB).^[^ ^219^ ^]^ The fluorine atoms in TPFPB molecules exhibit strong interactions with Na^+^ ions, which are the primary charge carriers in the original p‐type i‐TE. These strong TPFPB/Na^+^ interactions not only trap Na^+^ ions but also disrupt their interactions with the PVDF‐HFP polymer matrix, thereby hindering Na^+^ ion transport along the polymer chains. As a result, the TFSI^−^ anions become the dominant charge carriers, effectively switching the material to n‐type behavior. Furthermore, adjusting the concentration of TPFPB enables fine‐tuning of both polarity and magnitude of the thermopower.

**Figure 8:**
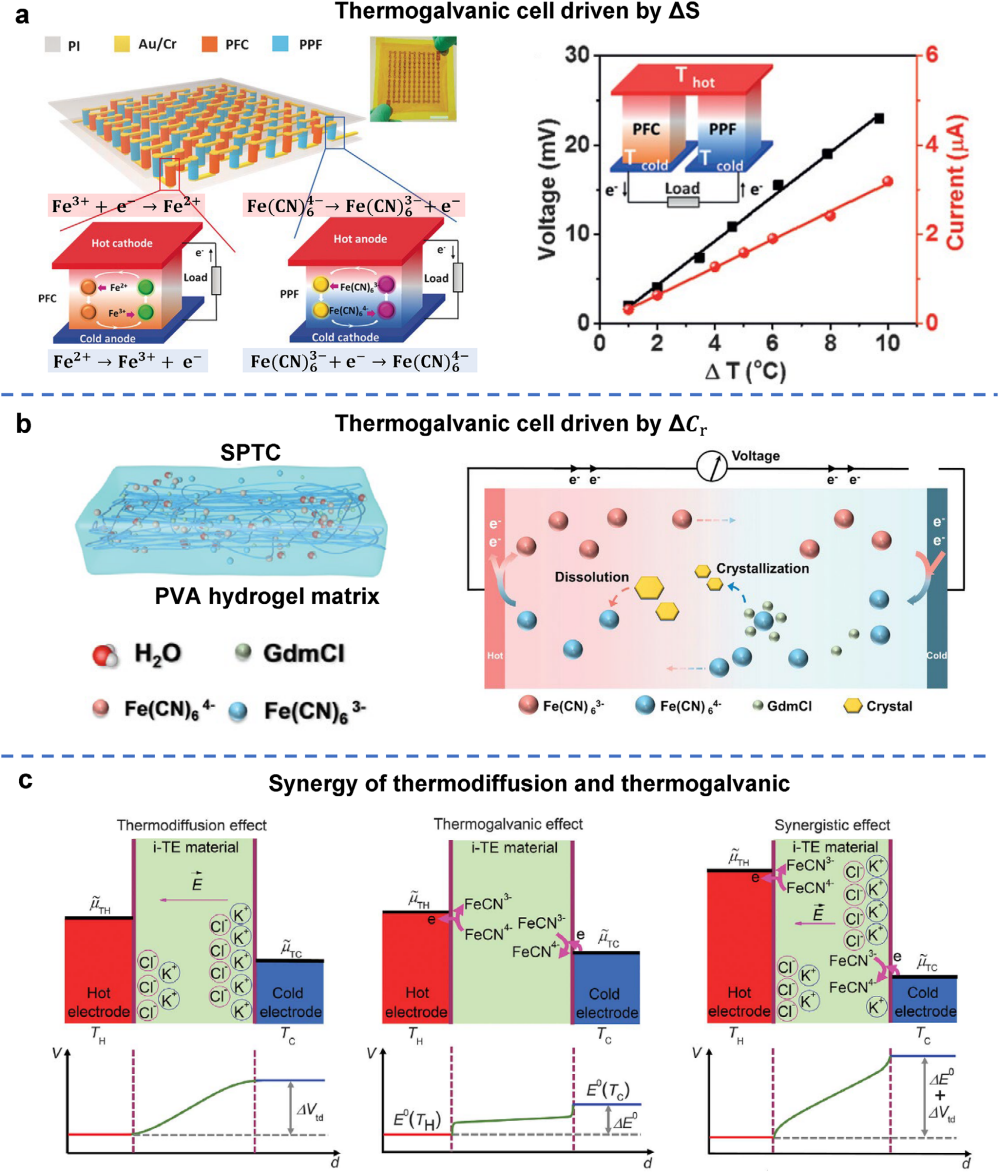
a) The integrated gel‐based thermocell and the voltage and current as a function of the temperature difference. Reproduced with permission.^[^ ^232^ ^]^ Copyright 2016, Wiley‐VCH. b) PVA hydrogel matrix thermogalvanic cell with a schematic illustration of the Gdm^+^ contribution to the thermogalvanic effect. Reproduced with permission.^[^ ^244^ ^]^ Copyright 2023, Wiley‐VCH. c) Synergistic combination of thermodiffusion and thermogalvanic. Reproduced with permission.^[^ ^223^ ^]^ Copyright 2020, AAAS.

The thermopower of a thermogalvanic cell is primarily governed by two factors: the solvent‐dependent entropy difference (ΔS) between the redox ions and the concentration ratio difference (Δ*C_r_*) between the hot and cold sides. In the commonly used redox couple, Fe(CN)_6_^4−^/Fe(CN)_6_^3−^, the anion Fe(CN)_6_^4−^ possesses a lower solvation entropy than Fe(CN)_6_^3−^. This ΔS drives the spontaneous oxidation of Fe(CN)_6_^4−^ into Fe(CN)_6_^3−^ at the hot side, releasing an electron that travels through the external circuit and is consumed at the cold electrode during the reduction of Fe(CN)_6_^3−^. Similarly, for the Fe^2+^/Fe^3+^ redox couple, the reduction of Fe^3+^ to Fe^2+^ has a positive ΔS, primarily due to differences in hydration. As a result, this reduction is favored on the hot side, drawing electrons from the electrode. Conversely, at the cold electrode, the oxidation of Fe^2+^ to Fe^3+^ occurs, releasing electrons to the external circuit. By embedding these redox couples into hydrogel matrices, the directionality of electron flow – whether release/draw (as in Fe(CN)_6_^4−^/Fe(CN)_6_^3−^) or draw/release (as in Fe^2+^/Fe^3+^) – can be manipulated to construct thermogalvanic cells that function analogously to the n‐type or p‐type legs in traditional thermoelectric generators. By connecting these n‐type and p‐type thermogalvanic elements in series, and exploiting the mechanical flexibility of hydrogels, one can fabricate wearable devices capable of harvesting body heat and converting it into usable electrical energy (**Figure** **8**a).^[^ ^232^ ^]^

Beyond leveraging entropy difference, Zhou's group developed a thermosensitive crystallization strategy to activate the typically negligible concentration ratio difference, Δ*C_r_*, for further enhancing the thermopower.^[^ ^221^ ^]^ In this strategy, guanidinium cations (Gdm^+^), which interact more strongly with Fe(CN)_6_^4−^ than with Fe(CN)_6_^3−^, were introduced to selectively crystallize Fe(CN)_6_^4−^, especially on the cold side. This selective crystallization results in a reduced local concentration of Fe(CN)_6_^4−^ at the cold electrode, favoring the reduction of Fe(CN)_6_^3−^. Meanwhile, the crystallized Fe(CN)_6_^4−^ dissolves at the hot side, enriching its concentration and facilitating its oxidation. As a result, a high Seebeck coefficient of 3.73 mV K^−1^ was achieved in a liquid‐state thermocell. This thermosensitive, selective crystallization approach was later adapted for hydrogel‐based ionic TEs. Figure 8b illustrates one such Δ*C_r_*‐driven hydrogel thermogalvanic cell, in which a polyvinyl alcohol (PVA) hydrogel serves as the matrix for both the Fe(CN)_6_^4−^/Fe(CN)_6_^3−^ redox couple and guanidinium chloride (GdmCl), an additive that selectively induces crystallization of Fe(CN)_6_^4−^. Upon incorporation of Gdm^+^, Fe(CN)_6_^4−^ preferentially crystallizes on the cold side, thereby enhancing the reduction of Fe(CN)_6_^3‐.^ Simultaneously, the crystallized Fe(CN)_6_^4−^ dissolves more readily on the hot side, facilitating the oxidation of Fe(CN)_6_^4−^. This temperature‐dependent crystallization‐dissolution process reinforces the redox cycle and significantly improves the thermo‐to‐electric energy conversion efficiency. Note that these hydrogel‐based thermogalvanic cells exhibit significantly enhanced thermopower values of 4.4 mV K^−1^ and 6.5 mV K^−1^, compared to 3.73 mV K^−1^ achieved by their liquid‐state counterpart. Additionally, they also demonstrate exceptional mechanical stretchability, sustaining elongation up to 1300%.^[^ ^244^, ^245^ ^]^

Thermodiffusion and thermogalvanic effects can be synergistically combined within a single hydrogel ionic TE system, provided that the nature (n‐type or p‐type) of the thermodiffusion cell aligns with the sign of the thermogalvanic Seebeck coefficient.^[^ ^223^, ^246^ ^]^ For example, a gelatin hydrogel infused with KCl behaves a p‐type thermodiffusion thermocell, while the same matrix loaded with the redox couple, Fe(CN)_6_^4−^/Fe(CN)_6_^3−^, exhibits a positive Seebeck coefficient (Figure 8c). By incorporating both KCl and Fe(CN)_6_^4−^/Fe(CN)_6_^3−^ into the gelatin hydrogel, a remarkable thermopower of 17 mV K^−1^ was achieved. A wearable device composed of 25 such synergistic thermocells generated over 2 V and a peak power of 5 mW from body heat alone.^[^ ^223^ ^]^

Hydrogel‐based ionic TEs stand out by combining high thermopower with the mechanical adaptability of soft materials, outperforming conventional ionic TE systems such as liquid‐state thermocells. This combination makes them ideal for harvesting low‐grade heat, particularly from the human body and microelectronics. Their intrinsic softness, flexibility, and adhesiveness enable conformable contact with irregular heat sources, making them suitable for applications, such as body heat harvesting,^[^ ^165^ ^]^ self‐powered thermal sensors,^[^ ^237^ ^]^ and human motion sensors,^[^ ^234^ ^]^ and electronic skins for physiological perception.^[^ ^247^ ^]^

In addition to their superior flexibility, hydrogel ionic TEs also exhibit other unique features, such as self‐healing,^[^ ^216^, ^239^, ^248^ ^]^ wide materials selection, and tunable properties for adapting to various environmental conditions. For example, tough and stretchable thermoelectric hydrogels can be created by applying a double‐network (DN) design strategy, originally developed for robust hydrogels, thus enabling practical deployment even in harsh conditions for low‐grade heat recovery.^[^ ^165^, ^234^, ^242^ ^]^ Hydrogels also accommodate diverse functional additives. The incorporation of hygroscopic salts like LiCl imparts environmental resilience, including enhanced water retention, freeze resistance, and self‐regeneration. A notable example is a LiCl‐infused polyacrylamide (PAAm) hydrogel‐based ionic thermoelectric generator (ITEG), which maintained its water content for 7 days under ambient conditions (302 K, 65% RH) and remained unfrozen at 253 K. Remarkably, this ITEG could absorb atmospheric moisture to self‐replenish the water loss at high temperatures, autonomously restoring both its original state and TE performance. It maintained its thermoelectric function at 258 K, and retained a Seebeck coefficient of 11.3 mV K^−1^ even after a drying‐regeneration cycle.^[^ ^233^ ^]^ Compared with traditional TEs, hydrogel ionic TEs also benefit from facile and sustainable fabrication processes. By utilizing natural polymers such as cellulose and environmentally friendly solvents like ionic liquids, green and scalable production of biodegradable, low‐cost hydrogel ITEGs becomes feasible.^[^ ^222^, ^238^ ^]^

### 3.3 Conversion of Chemical Potential Energy

Energy exchange or conversion is ubiquitous during the interactions between different materials. For example, moisture sorption by sorbents is a process accompanied by changes in the chemical potential energy of water between the gaseous state and the absorbed state.^[^ ^249^, ^250^ ^]^ When freshwater and saltwater are brought into contact across a semipermeable membrane, the difference in the chemical potential energy of water in salinity‐gradient solutions leads to the so‐called osmotic energy. To harness these chemical potential energies and convert them into useful electricity, technologies such as moisture‐electricity generation (MEG)^[^ ^250^, ^251^, ^252^ ^]^ and osmotic energy harvesting (OEH) have been developed. Introducing hydrogels into both MEG and OEH will bring significant enhancement in device performance, manufacturing approaches featured by simplicity, economy, and scalability, and prospects in soft and biological electronics. A summary of relevant works is presented in **Table** **4** at the end of this sub‐section.

**Table 4:** Summary of hydrogels for natural and biological environmental energy harvesting—Chemical potential.

Mechanisms	Key roles played by hydrogels	Hydrogels' properties required	Typical hydrogels: synthesis and performance	Applications	Refs.
**MEG**	Capture moisture Offer high ion density Fast ion transport Multiple‐functionalization	Hydrophilic, hygroscopic, porous Abundant functional groups on polymeric chains Ionic conductive Flexibility in modification	Poly(vinyl alcohol) (PVA)‐phytic acid (PA) and glycerin, gelation Humidity range: 10%‐ 85%RH Energy output: 0.88 V, 240 µA cm^−2^, 35 µW cm^−2^	Energy generation	[134]
			Acrylamide, 2‐acrylamide‐2‐methyl propane sulfonic acid, and LiCl, gelation Humidity range: 30%‐ 100%RH Energy output: 0.81 V, 480 µA cm‐2, 53.3 µW cm^−2^	Energy generation	[124]
			Nafion‐pNIPAm hydrogel, carbon paper as electrodes Humidity range: 68.5%‐ 82.9%RH Energy output: 1.86 V, 21.8 µA cm^−2^, 10.14 µW cm^−2^	Energy generation	[264]
**Streaming potential**	Enable liquid streaming Ionic selectivity Self‐regulating hydration	Porous structure Controllable porous structure chemical properties High hydration, hygroscopic to capture moisture	Functionalized conductive carbon black onto a 3D PU sponge with PVA Humidity range: 40%‐ 95%RH Energy output: 0.66 V, 63 µA, 8.1 µW	Energy generation	[265]
			Polyacrylic acid hydrogel with K+ ions Humidity range: 20%‐ 95%RH Energy output: 0.2 V, 12 µA, 23.75 µA cm^−2^, 0.48 µW	Energy generation	[266]
			Salt infused polyvinyl alcohol hydrogel on carbon‐black coated non‐woven fabric Humidity range: 10%‐ 100%RH Energy output: 0.65 V, 3 µA, 70 µW cm^−3^	Energy generation	[123]
			MXene (Ti3C2Tx) ‐polyacrylamide (PAM) ionic hydrogel Humidity range: 20%‐ 95%RH Energy output: 0.6 V, 1160 µA cm^−2^, 24.8 µW cm‐2	Energy generation	[268]
**Osmotic Energy**	Ionic selectivity and ionic flux Biomimetic soft power	Controllable porous structure chemical properties biocompatible, high hydration, “solid liquid”	2‐hydroxyethyl methacrylate phosphate (HEMAP) hydrogels Energy output: 5.38 W m^−2^ & 100 A m^−2^ at 50‐fold salinity gradient, 20.54 W m^−2^ at 500‐fold	Energy generation	[280]
			Bacterial cellulose‐(acrylic acid‐co‐acrylamide‐co‐methyl methacrylate) hydrogel Energy output: 7.63 W m^−2^ at 50‐fold salinity gradient, pH 11, 45.5 W m^−2^ at acid condition	Energy generation	[281]
			Natural balsa wood‐confined sodium polyacrylate (PAAS) polyelectrolyte hydrogel Energy output: 8.5 W m^−2^ at 50‐fold salinity gradient, 10.6 W m^−2^ at sea/river water	Energy generation	[282]
			Janus negatively charged PAEK‐HS and positively charged PES‐Py Energy output: 5.10 W m^−2^ at 500‐fold salinity gradient, 2.66 W m^−2^ at sea/river water	Energy generation	[283]
			Zwitterionic gradient double‐network hydrogel membrane (ZGDHM) Energy output: 5.44 W m^−2^ at 50‐fold salinity gradient, 49.6 W m^−2^ at 500‐fold	Energy generation	[286]
			Polysaccharide (chitosan and sodium alginate) polyelectrolyte hydrogel Energy output: 19.41 W m^−2^ at 500‐fold salinity gradient, 7.87 W m^−2^ at sea/river water	Energy generation	[287]
			Poly (ionic liquid) (PIL) membrane Energy output: 4.33 W m^−2^ at 50‐fold salinity gradient, 15.46 W m^−2^ at 500‐fold	Energy generation	[288]
			Charged polyelectrolyte hydrogel layer on a porous aramid nanofibers (ANF) membrane Energy output: 5.06 W m^−2^ at sea/river water	Energy generation	[166]
			AAc‐co‐AAm‐co‐(MMA hydrogel nanofibers in the 1D cylindrical nanopores of PC film Energy output: 11.72 W m^−2^ at 500‐fold salinity gradient	Energy generation	[290]
			Polyanion electrolytes in interlayer spacings of graphene oxide membranes Energy output: 4.94 W m^−2^ at 50‐fold salinity gradient, 14.7 W m^−2^ at 428‐fold, 34.1 W m^−2^ at 50‐fold HCl gradient	Energy generation	[292]

#### 3.3.1 Moisture Energy Harvesting

The technology of moisture‐electricity generation (MEG) refers to electricity generation through interactions between materials and gaseous water molecules.^[^ ^249^ ^]^ With the moisture‐materials interaction, the gaseous water molecules are bonded to the material surface by a physical or chemical sorption process, transitioning from a free gaseous state to an absorbed state. The gaseous‐to‐absorbed transition is accompanied by the changes of water molecules in their chemical potential energy as well as an ionization process to release free ions, which, mediated by asymmetrically structured or specially perforated materials, can be converted into electricity. Asymmetrically‐structured materials, such as graphene oxide films with gradient functional groups,^[^ ^253^ ^]^ or with differences in moisture penetration,^[^ ^254^ ^]^ produce a concentration gradient of the moisture‐liberated ions. Driven by this ionic gradient, ions start to migrate from the high‐concentration to the low‐concentration end, triggering the electronic flow in the external circuit to output electricity. This is the so‐called ionic gradient mechanism^[^ ^255^ ^]^ behind MEG.

Another mechanism is the streaming potential, which explains the electricity generation in specially perforated materials that have charged micro‐/nanochannels for water streaming.^[^ ^256^ ^]^ Once water molecules come into contact with a charged surface, an electronic double layer (EDL) is formed, which is highly ionic selective to favor the movement of counterions (with a charge sign opposite to the charged surface), especially along nanoscale channels. Continuous water streaming through these specially‐perforated materials leads to the spatial separation of ions and counterions, generating an electrical potential and current.^[^ ^252^, ^255^ ^]^ It has to be noted that for the streaming potential‐based MEG, the electric energy is converted not only from the changes of water molecules in chemical potential energy changes during the gaseous‐to‐adsorbed transition but also from the latent heat during water evaporation.^[^ ^252^ ^]^ Primarily based on these two mechanisms, many MEG devices have been developed using a variety of materials, including graphene oxides,^[^ ^253^ ^]^ porous carbon materials,^[^ ^257^ ^]^ polymers,^[^ ^258^ ^]^ polymer nanowires,^[^ ^259^ ^]^ metal oxide nanowires,^[^ ^260^ ^]^ natural wood,^[^ ^261^ ^]^ protein nanowires,^[^ ^262^ ^]^ among others. Despite rapid advances, MEG still faces challenges in enhancing and stabilizing its output power, minimizing environmental and equipment‐related constraints, and scaling up the MEG devices.

##### Ionic‐Gradient‐Driven Systems

**Figure 9:**
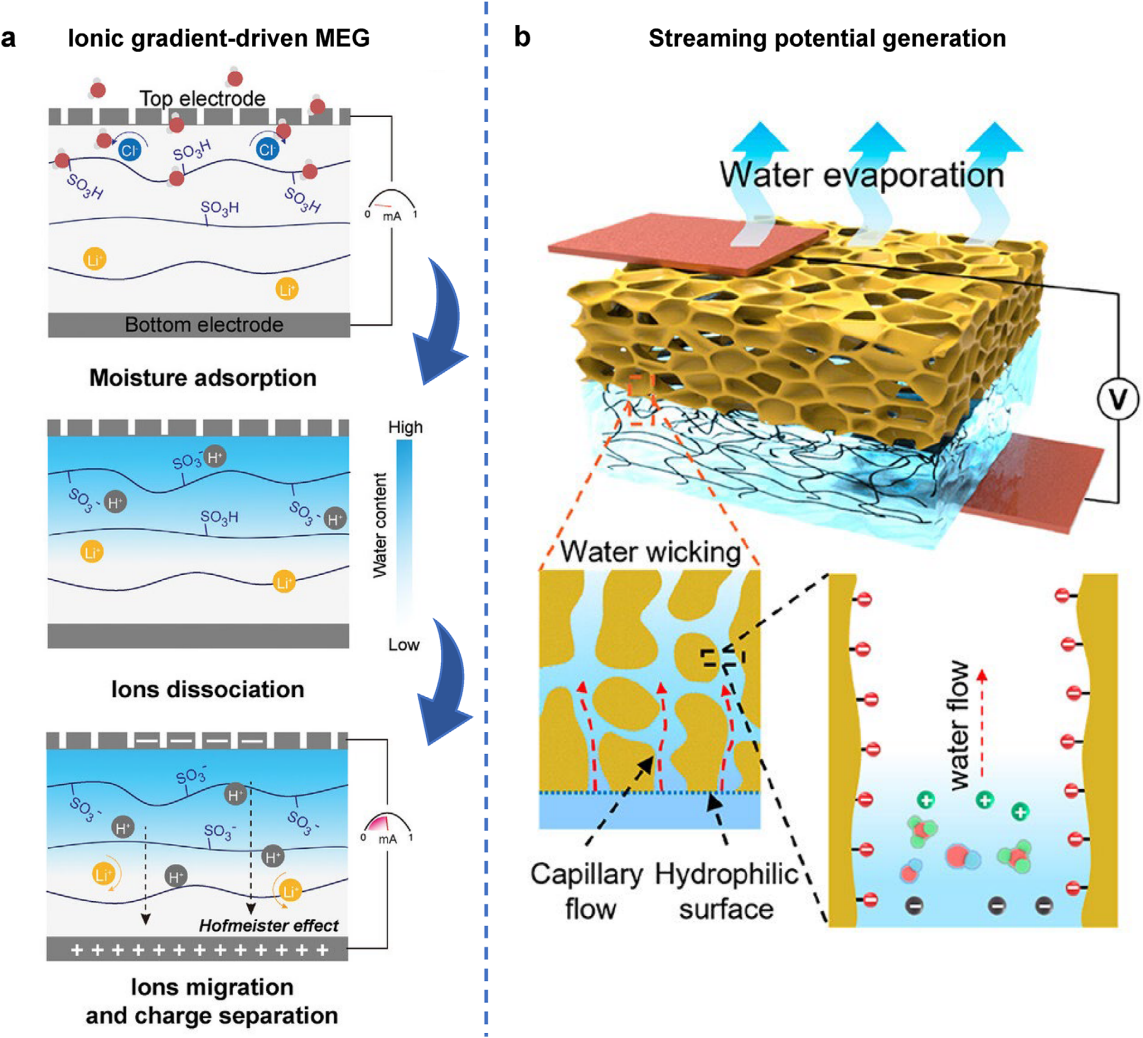
a) Schematic showing the power‐generation mechanism of the ionic gradient‐driven MEG. Reproduced with permission.^[^ ^124^ ^]^ Copyright 2023, Wiley‐VCH. b) PAAm hydrogel integrated with a hydrophilic MXene (Ti_3_C_2_T_x_) aerogel to create a bilayer, free‐standing and film‐type MEG with long‐term electricity generation. Reproduced with permission.^[^ ^268^ ^]^ Copyright 2023, American Chemical Society.

The key to enhancing MEG performance is developing materials with excellent capabilities to capture moisture, liberate substantial free ions, and selectively facilitate ion diffusion. All these characteristics can be found in hydrogels with excellent water‐capturing ability and rapid ion transport through their 3D hierarchical pores.^[^ ^57^ ^]^ Abundant hydrophilic polymeric chains in hydrogels ensure strong moisture interaction and provide abundant dissociated free ions for MEG. The 3D hierarchical and interconnected pores host large amounts of water (or other solvents) and enable fast ion transport within hydrogels. The interaction between ions and the hydrogel polymeric backbone can be readily tailored to control the ionic selectivity. By a rational combination of poly(vinyl alcohol) (PVA), phytic acid (PA), and glycerol‐water binary solvent, an ionic hydrogel moisture‐electricity generator (IHMEG) is developed. This combination of hydrophilic PVA, PA, and hygroscopic glycerol grants the IHMEG a high moisture sorption capability, which, coupled with the proton doping by PA as well as the ionic water clusters as vehicles for proton transport, allows the IHMEG extensive proton dissociation and fast migration through a robust hydration effect.^[^ ^134^ ^]^ As a result, a single IHMEG unit of 0.25 cm^2^ can deliver a stable open‐circuit voltage of 0.8 V continuously for more than 1000 h, a high short‐circuit current density of 0.24 mA cm^−2^, and a power density of up to 35 µW·cm^−2^ which is 500 times that of a graphene oxide‐based MEG^[^ ^263^ ^]^ and seven times that of a protein‐based MEG.^[^ ^262^ ^]^ Later in 2023, Li's group reported a MEG based on hydrogels molecularly engineered via the impregnation of lithium ions and sulfonic acid groups into the polymer molecular chains (**Figure** **9**a). The addition of lithium ions and sulfonic acid imparts the hydrogel with enhanced moisture capturing. In addition, the sulfonic acid groups provide the hydrogel with a robust 3D network structure, improving mechanical properties and also proton dissociation. The lithium ions disrupt hydrogen bonds between polymer chains via the Hofmeister effect, expanding the polymeric network and thus facilitating fast proton transport within the hydrogels. The enhanced hygroscopicity, proton concentration, and ionic conductivity are finally translated into a high voltage of 0.81 V and a current density of 0.48 mA cm^−2^.^[^ ^124^ ^]^ By combining the strong moisture capture capability of hydrophilic poly(N‐isopropyl acrylamide)) with the abundant moisture‐dissociable protons of ionic polymer, a hydrogel‐based hydrovoltaic device ensures a large amount of moisture‐liberated protons, further augmenting the output voltage to 1.86 V.^[^ ^264^ ^]^

##### Streaming Potential‐Driven Systems

Continuous water streaming is required for the streaming potential‐based MEG to yield stable electricity, often necessitating the employment of auxiliary equipment like a pump or tank for bulk water manipulation. This inevitably complicates the device and also causes an energy penalty. Owing to their exceptional capability to store water within a solid or quasi‐solid network, hydrogels are utilized as a water reservoir to enable a sustained water flow through the process of water evaporation. A water‐saturated superabsorbent hydrogel was employed to fabricate a MEG, where the bottom end of a three‐dimensional sponge (3DS) coated by functionalized carbon black (FCB) and polyvinyl alcohol (PVA), or PVA@FCB@3DS was inserted into the hydrogel to draw water. Driven by the capillary effect and water evaporation on the surface of PVA@FCB@3DS, water can directionally move from the bottom to the top to generate electricity. As the hydrogel holds a lot of water, the water flow and power output can last for more than 150 h at ambient conditions (20.4 °C, 55% RH).^[^ ^265^ ^]^ Due to the water evaporation, ions tend to accumulate at the air‐hydrogel interface, creating a high ionic gradient between the interface and bulk hydrogel. This ionic gradient can drive the transport of ions with larger mobility and lead to the electrical output with a prolonged lifetime. Such air–hydrogel interface‐induced electricity generator (AIEG) has been demonstrated by a KOH‐impregnated poly(acrylic acid), PAA hydrogel, where a voltage output of 0.2 V sustained for over one month, and a current density of 23.75 µA cm^−2^ lasted for more than one week without obvious decay.^[^ ^266^ ^]^

In addition to the exceptional capability to hold water, most hydrogels are hygroscopic, and their capability to capture atmospheric water can be readily tuned by incorporating various additives, creating hydrogels with self‐regulating and self‐replenishing hydration states. Using these quasi‐eternally‐hydrated hydrogels, self‐sustained MEG is developed, where spontaneous moisture sorption and evaporation are combined to yield sustainable and bulk water‐free electricity generation. ^[^ ^267^ ^]^ Typically, a self‐sustaining MEG device consists of three parts, namely the hygroscopic, the transmission, and the evaporation part. Hydrogels are commonly employed in the hygroscopic part, where they autonomously capture moisture from the air to continuously supply water to the transmission and evaporation part for sustained electricity generation. A hygroscopic ionic hydrogel has been asymmetrically deposited on a functionalized carbon layer to fabricate a MEG, and this MEG can yield a durable in‐plane voltage of over 0.6 V because a “permanent” water gradient and resulting water streaming from the wet hydrogel‐deposited zone to the dry hydrogel‐free zone is established using hydrogels.^[^ ^123^ ^]^ Besides the in‐plane asymmetrical configuration, hygroscopic hydrogels can also be integrated into a MEG in a vertically stacked manner. A polyacrylamide (PAAm) hydrogel film has been integrated with a hydrophilic MXene (Ti_3_C_2_T_x_) aerogel to create a bilayer, free‐standing and film‐type MEG capable of generating electrical output of up to 0.6 V. When the PAAm hydrogel is converted into a PAAm organo‐hydrogel via a solvent‐exchange process, this MEG device achieves a long‐term electricity generation lasting over 15 days (Figure 9b).^[^ ^268^ ^]^

Beyond enhancing performance, hydrogel‐based MEGs offer other advantages over conventional MEGs that should not be overlooked. These include 1) flexibility, 2) broad environmental applicability, and 3) easy and scalable fabrication. First, hydrogel‐based MEG inherits the flexibility of hydrogels, laying the ground for developing flexible and stretchable power sources for soft and wearable electronics. A molecular‐engineered PAM hydrogel exhibits a high stretchability of up to 506%, combined with remarkable toughness, compression resistance, and solid‐like elasticity, indicating its promising applications in self‐powered protective wearables.^[^ ^124^ ^]^ By adopting a double network strategy, the stretchability of a tamarind gum (TG)‐polyacrylamide (PAM) hydrogel can reach 2000%.^[^ ^269^ ^]^ Rationally combining hygroscopic ionic hydrogel with a soft, flexible textile, e.g., the carbon black‐coated cotton knitted fabric, a fully stretchable MEG has been developed, which can sustain a stretchability of 400% without compromising the electricity generation.^[^ ^270^ ^]^ By contrast, most conventional MEG active materials, such as graphene oxides, silicon nanowires, metal oxides, and protein nanowires, are rigid or have to be deposited on a rigid substrate,^[^ ^260^, ^262^, ^263^, ^271^ ^]^ showing a huge gap in their applications in soft and wearable electronics. Second, compared with conventional MEGs, most hydrogel‐based MEGs show greater environmental tolerance, capable of functioning reliably in diverse environments, owing to hydrogels’ highly hydrophilic, hydratable, and tailorable nature. For example, hydrogel‐based MEGs have demonstrated stable operation across a wide humidity and temperature range spanning from <10%RH to 100%RH and from −30 °C to 80 °C.^[^ ^50^, ^268^, ^272^, ^273^, ^274^ ^]^ In contrast, this wide environmental adaptability of the hydrogel‐based MEGs is rarely seen in traditional MEGs, which, beyond a narrow applicable window, often become degraded or even inoperable in output energy.^[^ ^262^ ^]^ Third, hydrogel‐based MEGs are easy to fabricate and scale up. A variety of widely available, economic, synthetic or natural polymers and monomers can be physically or chemically cross‐linked into hydrogels. A wide selection of additives and methods can be employed to tailor hydrogels’ properties for multiple purposes. A range of assembly approaches, such as printing or modeling, can be implemented to scale up the device.^[^ ^50^, ^124^, ^134^, ^270^, ^272^, ^273^, ^275^, ^276^ ^]^ By comparison, complicated chemical, hydrothermal, or electrochemical reactions are needed to prepare traditional MEG raw materials such as graphene oxides, MoS_2_, TiO_2_ nanowires, and protein nanowires. Then for fabricating an MEG, the vacuum filtration process as well as various pretreatments are often required,^[^ ^260^, ^262^, ^263^, ^271^ ^]^ complicating the fabrication of MEG devices and hindering their mass production and applications.

#### 3.3.2 Osmotic Energy Harvesting

Another renewable, sustainable, and abundant chemical energy is the osmotic energy, or salinity gradient energy, which exists in solutions with different solute concentrations, such as seawater and river water. This osmotic energy can be harvested and converted into electricity via reverse electrodialysis (RED).^[^ ^277^, ^278^, ^279^ ^]^ The key component in RED systems is an ion‐selective transport membrane, allowing anions or cations to selectively pass through the membrane for electricity generation. Traditional ion‐exchange membranes, along with more recent advancements in 1D and 2D nanochannels, fall short of meeting the essential requirements for RED, thereby impeding the practical implementation of RED for osmotic energy harvesting. These requirements include high‐power density, mechanical and chemical stability, and scalable fabrication processes. Hydrogels, distinguished by their 3D voluminous interconnected porous structure and high ionic conductance, offer a promising solution. Their superior ion transport properties, complemented by inherent space charge characteristics, broad material selection, versatile modification techniques, and ease of large‐scale fabrication, position hydrogels as an optimal choice for fulfilling all the critical criteria for effective RED development. In a RED system, hydrogels can function as stand‐alone membranes, either symmetric or asymmetric, or they can be integrated into 1D and 2D nanochannels to enhance RED performance. Moreover, by introducing hydrogels into osmotic energy, soft, solid RED energy generators can also be developed.

##### Membrane‐Driven Systems

**Figure 10:**
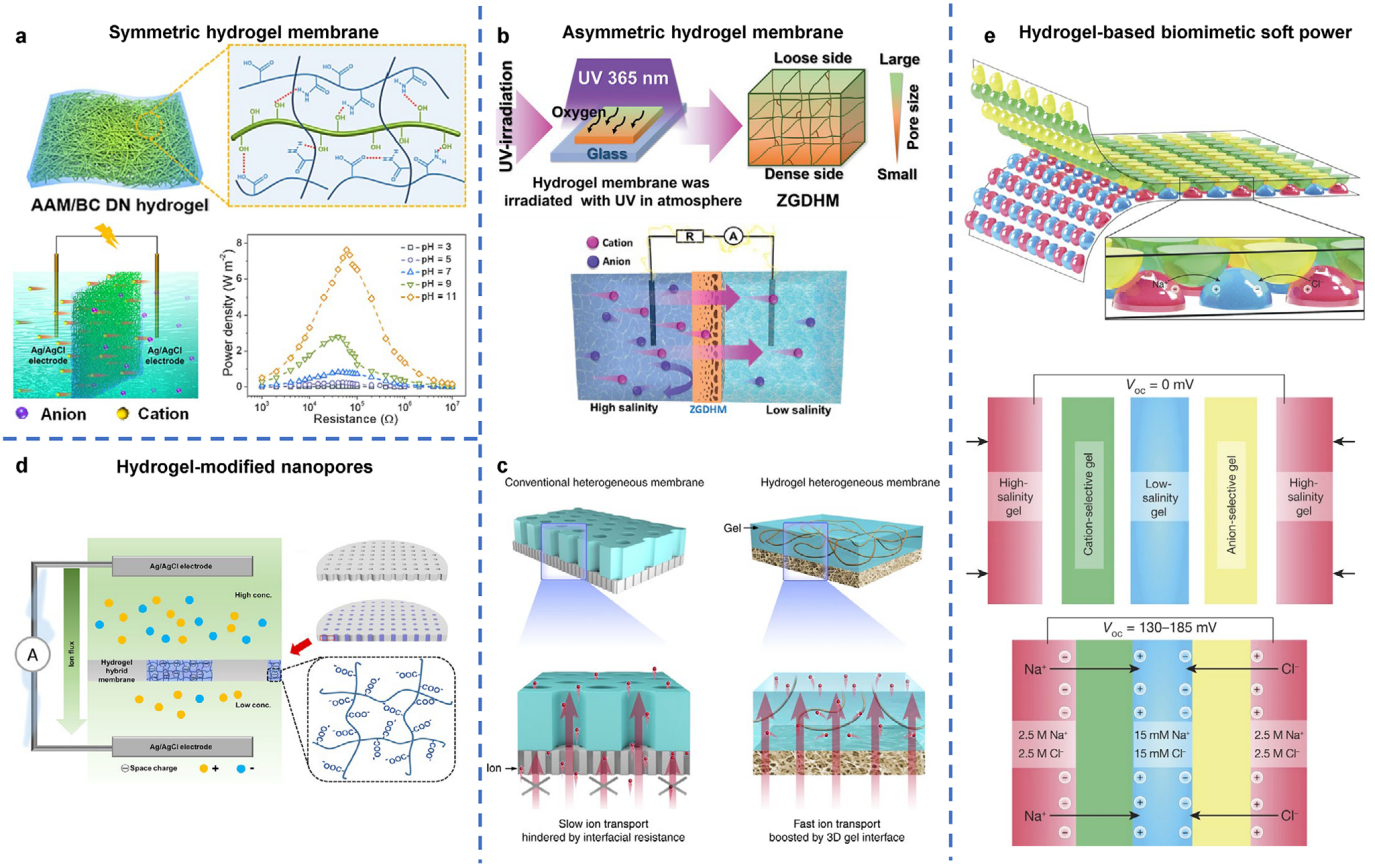
a) AAM/BC DN hydrogel with an interconnected nanoporous structure giving a high output power density. Reproduced with permission.^[^ ^281^ ^]^ Copyright 2023, Elsevier. b) Zwitterionic gradient double‐network hydrogel membrane (ZGDHM) with a gradient pore structure and ionic selectivity. Reproduced with permission.^[^ ^286^ ^]^ Copyright 2023, Wiley‐VCH. c) hydrogel‐based heterogeneous membrane featuring 3D gel interface with high ion transport efficiency. Reproduced under the terms of the CC‐BY Creative Commons Attribution 4.0 International license (https://creativecommons.org/licenses/by/4.0).^[^ ^166^ ^]^ Copyright 2020, The Authors, published by Springer Nature. d) Schematic depiction of the cation selectivity of the hydrogel hybrid membrane due to negatively charged nanochannels. Reproduced with permission.^[^ ^290^ ^]^ Copyright 2020, American Chemical Society. e) Tetrameric repeating units composed of a high‐salinity hydrogel, a cation‐selective hydrogel, a low‐salinity hydrogel, and an anion‐selective hydrogel in sequence, and the voltage generation mechanism. Reproduced under the terms of the CC‐BY Creative Commons Attribution 4.0 International license (https://creativecommons.org/licenses/by/4.0).^[^ ^293^ ^]^ Copyright 2017, The Authors, published by Springer Nature.

In symmetric stand‐alone membranes, the 3D interconnected and space‐charged nanopores within hydrogels, especially, polyelectrolyte hydrogels,^[^ ^280^, ^281^, ^282^ ^]^ facilitate ionic transport and selectivity, two pivotal factors crucial for RED. This characteristic has led to widespread exploration of hydrogels as potential replacements for conventional RED membranes, which often suffer from inadequate power density and require complex and cost‐intensive fabrication processes. For example, through simple photopolymerization, monomers of negatively charged 2‐hydroxyethyl methacrylate phosphate (HEMAP) can be assembled into HEMAP hydrogels with a 3D interconnected porous structure. Furthermore, by controlling the membrane thickness at the micrometer scale, the pore size of HEMAP hydrogels can be reduced to around 5–7 nm, approaching the Debye screening length and ensuring charge‐governed ion transport within the hydrogels. These 3D interconnected and negatively charged nanostructures within HEMAP hydrogels yield an output power density of 5.38 W m^−2^ at a 50‐fold salinity gradient, surpassing the benchmark of several commercial membranes.^[^ ^280^ ^]^ In addition to controlling the membrane thickness, charged monomers such as acrylic acid can be infused into the micropores of bacterial cellulose (BC) hydrogels, giving rise to a negatively charged 3D network with an interconnected nanoporous structure, and hence achieving a high output power density of up to 7.63 W m^−2^ at a 50‐fold salinity gradient (**Figure** **10**a).^[^ ^281^ ^]^

These examples highlight how optimizing symmetric hydrogel membranes by tuning thickness or incorporating charged monomers can significantly enhance power output in RED systems. However, to further improve ionic selectivity, suppress concentration polarization, and push energy conversion efficiency even higher, researchers have increasingly turned to asymmetric membrane designs. Asymmetric nanofluid membranes exhibit either chemical or geometrical asymmetry, such as variations in pore structure, charge distribution, or wettability across the membrane cross‐section. The breakdown of symmetry imparts to the membranes with a unique property known as the ionic diode effect or current rectification. This effect leads to the preferential passage of ions through the membrane in one direction. By exploiting this ionic diode effect, asymmetric membranes can effectively mitigate ionic concentration polarization. Consequently, this enhances not only the efficiency of ionic transport and selectivity but also the osmotic energy conversion across these membranes.^[^ ^283^, ^284^ ^]^ Traditionally, asymmetric membranes are created by physically or chemically integrating two distinct layers, each serving as the support and the selective layer, respectively.^[^ ^284^, ^285^ ^]^ This process often utilizes techniques like spin coating or vacuum filtration, which are often impractical for large‐scale production. In contrast, within hydrogels, a simpler alternative has been developed: introducing asymmetry during the cross‐linking of polymeric chains to form hydrogels. For instance, in the creation of a gradient pore structure within a zwitterionic gradient double‐network hydrogel membrane (ZGDHM), a two‐step photopolymerization method is employed, where the oxygen diffusion into the initially polymerized hydrogel is leveraged to regulate the crosslinking density of the subsequently polymerized hydrogel, thereby establishing a gradient pore structure. The resulting ZGDHM demonstrates ultrahigh ionic selectivity, the ionic diode effect, and space‐charge‐governed ion transport, culminating in an impressive output power density of up to 5.44 W m^−2^ at a 50‐fold salinity gradient (Figure 10b).^[^ ^286^ ^]^ Besides oxygen diffusion, inhomogeneous reaction diffusion^[^ ^287^ ^]^ and precursor diffusion^[^ ^288^ ^]^ within hydrogels can also be employed to fabricate asymmetric hydrogel membranes.

Also, the challenge of balancing ion transport with selectivity in membranes becomes increasingly pronounced with asymmetric configurations. Mismatches in pore alignment between distinct layers and inadequate coupling between channels of varying dimensions can impede ion transport at the interface, ultimately resulting in diminished osmotic energy conversion efficiency. Integrating hydrogels into asymmetric membranes can address these challenges by imposing a high osmotic pressure for liquid transport^[^ ^289^ ^]^ and eliminating mismatches in pore and channel alignment. Moreover, with the incorporation of a charged network (e.g., polyelectrolyte hydrogel), this integration could significantly enhance the ion transport efficiency and, consequently, improve salinity gradient power generation. For example, through a sequential blade‐casting method, a polyelectrolyte hydrogel layer was combined with a supporting porous layer made from aramid nanofibers (ANF), forming a hydrogel/ANF asymmetric membrane where the hydrogel layer provides a highly negatively charged 3D network for enhancing ion transport and selectivity across the heterogeneous membrane. As a result, this hydrogel/ANF membrane maintains the ionic diode effect and achieves a power output of 5.06 W m^−2^ when seawater and river water are mixed (Figure 10c).^[^ ^166^ ^]^

Building upon progress in asymmetric membrane design, another promising approach involves integrating hydrogels into nanochannel‐based membranes to further enhance osmotic energy harvesting. Single or multiple 1D nanopore structures, as well as 2D nanochannels formed by stacking 2D nanosheets, are commonly employed as RED membranes for osmotic energy harvesting. To enhance energy conversion performance, polyelectrolyte hydrogels are introduced to modify the properties of these nanopores or nanochannels. The incorporation of hydrogels improves water interaction to facilitate ion transport and also adjusts surface charge concentration and polarity to enhance ion selectivity. Through radical‐initiated polymerization, acrylic acid (AAc)‐co‐acrylamide (AAm)‐co‐methyl methacrylate (MMA) hydrogel nanofibers can be constructed within the 1D cylindrical nanopores of a commercialized polycarbonate (PC) film, forming a hybrid membrane with high‐density negatively charged nanochannels. Under a 500‐fold salinity gradient, this hybrid hydrogel‐based RED achieves a peak power density of 11.72 W m^−2^ (Figure 10d).^[^ ^290^ ^]^ Negatively or positively charged hydrogels can functionalize nanopores of various geometries (e.g., cylindrical or conical shape), creating cation‐ or anion‐selective membranes. These selective membranes can be stacked to configure a full RED cell for salinity gradient energy conversion.^[^ ^291^ ^]^ In lamellar nanosheet‐stacked structures, such as graphene oxide membranes (GOMs), the interlayer spacings serve as 2D nanochannels for liquid transport. Introducing negatively charged polyanion electrolytes into these nanochannels, enhances the ion permeability and selectivity, yielding a maximum output power density of 4.94 W m^−2^ under a 50‐fold salinity gradient, 3.5 times higher than that achieved by pristine GOM.^[^ ^292^ ^]^

As discussed, negatively and positively charged hydrogels can function as cation‐ and anion‐selective membranes, respectively. Beyond selective ion transport, hydrogel membranes can also retain solutions with different concentrations, offering a pathway to develop soft RED systems in a solid form for salinity gradient energy conversion. Inspired by electric eels, a soft power concept was demonstrated by using tetrameric repeating units composed of a high‐salinity hydrogel, a cation‐selective hydrogel, a low‐salinity hydrogel, and an anion‐selective hydrogel in sequence (Figure 10e). According to the principle of RED, each tetrameric unit generates a potential of 130–185 mV. When 2449 units are stacked together, the voltage can reach up to 110 V.^[^ ^293^ ^]^ A similar integration of different‐salinity hydrogels and ion‐selective components is also found in a thermo‐osmotic gel system. In this system, free ions can be released from crystalline salts by asymmetrically heating the gel, creating a salinity gradient across the gel system. By incorporating a cation‐selective membrane, this salinity gradient energy can be efficiently converted into electricity.^[^ ^294^ ^]^

##### Capacitive Mixing and Entropy‐Driven Systems

Apart from membrane‐based systems, it is important to note the growing interest in complementary electrochemical strategies such as capacitive mixing (CapMix) and mixing entropy batteries, which also exploit salinity gradients between seawater and freshwater to harvest “blue energy.” Unlike classical RED, which depend on ion‐selective membranes, these systems use electrochemical cells with electrodes that cyclically adsorb and release ions to extract energy from salinity changes.

In CapMix systems, porous electrodes, often carbon‐based, undergo alternating charging and discharging cycles between high‐ and low‐salinity solutions, leveraging the chemical potential difference across the electrical double layer.^[^ ^295^ ^]^ The energy arises from the entropy gain when salt ions mix, offering high theoretical efficiency. Recent advances point to hydrogels as next‐generation materials to enhance CapMix performance. For example, hydrogel electrodes embedded with conductive polymers or carbon nanomaterials provide high surface area, tunable ion selectivity, and mechanical flexibility, improving ion transport and electrode capacitance.^[^ ^296^, ^297^ ^]^ Additionally, ionic or polyelectrolyte hydrogels can function as soft ion reservoirs, boosting the charge storage capacity of hybrid hydrogel–carbon electrodes. Mixing entropy batteries, another salinity‐gradient‐driven strategy, use redox‐active electrodes that change oxidation states in response to electrolyte salinity changes.^[^ ^298^ ^]^ Hydrogels can serve as electrolyte hosts or separators, improving mass transport and maintaining system stability over cycles.^[^ ^299^ ^]^ Incorporating stimuli‐responsive hydrogels that swell or contract with salinity fluctuations further offers dynamic control over electrode–electrolyte interactions.

Compared to membrane‐based osmotic systems, capacitive and entropy mixing devices offer potential advantages in mechanical robustness, system simplicity, and capital cost reduction. However, challenges remain in achieving long‐term cycling stability, maximizing ion selectivity, and scaling production. Integrating hydrogels into these systems presents exciting opportunities to overcome these limitations by combining ionic conductivity, flexibility, and tailored interfacial properties.

### 3.4 Conversion of Light Energy

Among the various forms of environmental energy available, solar light is the most abundant and easily harnessed for generating electricity or green fuels in a clean, sustainable manner. Traditionally, light‐to‐electricity conversion is achieved using photovoltaic cells. However, hydrogels can also be engineered to enable this conversion, for example, by incorporating photothermal materials. Mediated by a photothermal mechanism, hydrogels first absorb sunlight and turn it into heat, finally generating electricity via thermoelectric, thermogalvanic, or streaming potential technologies. Beyond electricity generation, hydrogels can also be designed to facilitate solar‐to‐fuel conversion, such as hydrogen generation through a photocatalytic process. Here, photocatalysts anchored within the hydrogel network absorb photons to generate electron‐hole pairs, driving redox reactions that produce hydrogen gas. In these applications, hydrogels serve as an ideal matrix for hosting photoactive (photothermal and photocatalytic) materials, reactants, and other media. Their ability to enhance light capture, facilitate mass/charge exchange, enable localized heating, and boost structural stability, will ultimately optimize solar energy harvesting. A summary of relevant works is presented in **Table** **5** at the end of this sub‐section.

**Table 5:** Summary of hydrogels for natural and biological environmental energy harvesting—Solar light (light source is 1 kW m^−2^ Xenon lamp (1 sun) unless stated otherwise).

Mechanisms	Key roles played by hydrogels	Hydrogels' properties required	Typical hydrogels: synthesis and performance	Applications	Refs.
**Photothermal**	Distribute, host photothermal materials Enhance light absorption Retard heat loss & localized heating	Abundant functional groups fixing photothermal materials Porous Low thermal conductivity & 3D interconnected structure	Matrix: PAAm‐CMC hydrogel with [Fe(CN)_6_]^4−^/^3−^ redox couple Photothermal materials: Pyrogallic acid (PA) and polyethyleneimine (PEI) Energy output: ‐1.40 mV K^−1^. 6.92 mV, 0.75 A m^−2^, 1.47 mW m^−2^ at ΔT = 1.9 K	Photothermal‐mediated electricity generation	[320]
			Matrix: Carboxymethylcellulose‐interpenetrated polyacrylamide network Photothermal materials: Carbon nanotubes (CNTs) Energy output: ‐1.26 mV K^−1^. 5.7 mV, 0.6 A m^−2^, 1.473 mW m‐2 at ΔT = 6.6 K	Photothermal‐mediated electricity generation	[321]
			Matrix: Polyacrylamide (PAM) hydrogel Photothermal materials: CuO micro‐flowers on Cu foil Energy output: 1.2 mV K^−1^. 10.3 mV, 0.9 A m‐2, 1.57 mW m^−2^ at ΔT = 8.7 K	Steam‐electricity cogeneration	[322]
			Matrix: PVA hydrogel Photothermal materials: Tea leaves residue Energy output: 58.5 mV at DT = 6.1 K. Evaporation rate: 0.91 kg m^−2^ h^−1^	Steam‐electricity cogeneration	[323]
			Matrix: Polyacrylamide (PAM) hydrogel Photothermal materials: Carbon nanotubes (CNTs) Energy output: 2.8 V, 65.8 mA (8 TE modules) at DT = 55 K. Evaporation rate: 1.42 kg m^−2^ h^−1^	Steam‐electricity cogeneration	[324]
**Photocatalytic**	Distribute, anchor, recycle catalysts Enhance light absorption Facilitate mass & charge transfer & product release	Abundant functional groups fixing catalyst, “solid liquid” Porous, anchor catalysts preventing catalysts agglomeration 3D interconnected network, porous	Matrix: Poly (acrylamide‐dimethylaminoethyl methacrylate) hydrogel Photocatalyst: CdS nanoparticles H_2_ generation: 10.35 µmol g^−1^ h^−1^	Hydrogen generation	[305]
			Matrix: Chitosan hydrogel Photocatalyst: Ag/TiO_2_ nanofibers H_2_ generation: 5190 µmol m^−2^. Evaporation: 5 kg m^−2^ (5 hours illumination)	Hydrogen and steam generation	[306]
			Matrix: Polyurethane‐poly(propylene glycol) hydrogel Photocatalyst: Pt/TiO_2_ nanoparticles H_2_ generation:163 mmol h^−1^ m^−2^ (1.2 sun)	Hydrogen generation	[52]
			Matrix: PNIPAM and alginate hydrogel Photocatalyst: Pt/C_3_N_4_ nanosheets H_2_ generation: 7437 µmol g^−1^ h^−1^	Hydrogen generation	[307]
			Matrix: PNIPAM hydrogel Photocatalyst: Au/g‐C_3_N_4_ nanoparticles H_2_ generation: 1061.82 µmol g^−1^ h^−1^	Hydrogen generation	[308]
			Matrix: Chitosan hydrogel Photocatalyst: Cu/ZnS H_2_ generation: 95.7 mmol m^−2^ h^−1^ (365 nm UV LED, intensity 150 mW cm^−2^)	Hydrogen generation	[53]

#### 3.4.1 Photocatalytic Energy Harvesting

The photocatalytic mechanism enables solar light harvesting and its conversion into green fuels, such as hydrogen gas (H_2_), which produces only water as a combustion byproduct. As a promising technology for clean and sustainable hydrogen generation from water using sunlight, photocatalytic water splitting has been extensively studied for decades.^[^ ^300^ ^]^ During the photocatalytic water‐splitting process, a photocatalyst, like semiconductor nanoparticles (NPs), absorbs photons from solar light, exciting electrons and holes to initiate reactions that decompose water into H_2_ and O_2_. In a typical photocatalytic system, photocatalyst particles are suspended in an aqueous environment. However, this approach suffers from poor light absorption due to severe light shielding and scattering effects and requires constant mechanical stirring to prevent particle aggregations. Additionally, recovering and recycling photocatalysts in such a particle‐suspended system is nearly impossible.^[^ ^301^, ^302^, ^303^ ^]^ An alternative approach is to immobilize the photocatalyst in a bed reactor. Despite its improvements in light absorption, aggregation prevention, and catalyst recycling, the bed reactor system faces a set of challenges related to matrix selection, light delivery, mass exchange, as well as gas separation.^[^ ^304^ ^]^ These limitations continue to hinder the practical applications of photocatalytic H_2_ generation.

However, the incorporation of hydrogels in photocatalytic H_2_ generation can overcome the aforementioned limitations due to their unique ability to uniformly distribute and anchor photocatalysts, enhance light absorption, facilitate mass exchange and charge transfer, and protect and stabilize photocatalysts even under harsh conditions. These advantages stem from the distinctive properties of hydrogels, including their 3D porous structure, large surface area, hydrophilicity, high water retention and ionic mobility, abundant functional groups, and tunable composition and structure. Leveraging the strong interaction of hydrogels with metal ions and their high solution absorption capability, Li et al.^[^ ^305^ ^]^ successfully synthesized a CdS‐embedded hydrogel via an in situ growth method. In this CdS hydrogel, photocatalysts, CdS NPs, were uniformly dispersed and strongly bonded to the polymer chains, ensuring large catalyst loading and high stability. Combined with the transparency of the hydrogel and the high diffusion rate of reactants facilitated by its high swelling capacity, this enhances light capture, improves photocatalyst resistance to photocorrosion, and ultimately boosts photocatalytic hydrogen production. In addition to CdS NPs, other photocatalysts, such as TiO_2_/Ag nanofibers,^[^ ^306^ ^]^ Pt/TiO_2_ or Cu/TiO_2_ nanocomposite,^[^ ^52^ ^]^ Pt‐loaded graphitic carbon nitride (Pt/g‐C_3_N_4_),^[^ ^307^ ^]^ Au‐decorated g‐C_3_N_4_,^[^ ^308^ ^]^ and Cu‐doped ZnS (Cu‐ZnS)^[^ ^53^ ^]^ have also been successfully incorporated into hydrogels. In all cases, catalyst‐integrated hydrogels outperform their suspended counterparts in hydrogen production.

**Figure 11:**
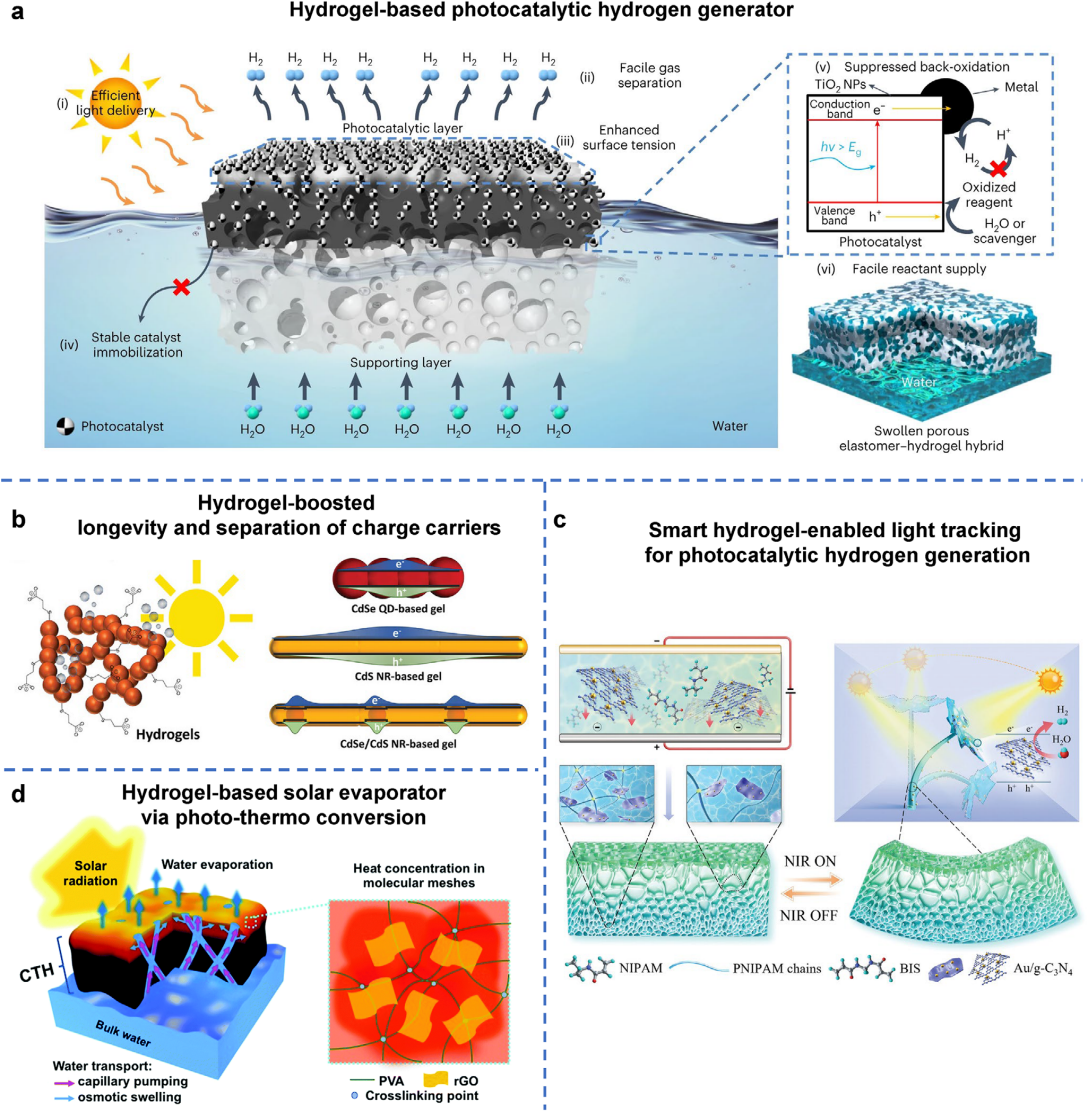
a) Floatable matrix engineered with hydrogel to immobilize photocatalysts for hydrogen production. Reproduced under the terms of the CC‐BY Creative Commons Attribution 4.0 International license (https://creativecommons.org/licenses/by/4.0).^[^ ^52^ ^]^ Copyright 2023, The Authors, published by Springer Nature. b) 3D, highly porous hydrogel network through a controlled destabilization of a ligand‐stabilized nanocrystal solution results in longevity and separation of charge carriers. Reproduced with permission.^[^ ^309^ ^]^ Copyright 2023, Wiley‐VCH. c) PNIPAM integrated with Au/g‐C_3_N_4_ to create a smart light tracking hydrogel for photocatalytic hydrogen generation. Reproduced with permission.^[^ ^308^ ^]^ Copyright 2022, Wiley‐VCH. d) Solar vapor generation based on hybrid hydrogels with capillary facilitated water transport. Reproduced with permission.^[^ ^327^ ^]^ Copyright 2018, Royal Society of Chemistry.

From these catalyst‐hydrogel systems, we also observe that beyond aiding catalyst distribution and anchoring, incorporating hydrogels into photocatalytic hydrogen generation offers a range of advantages. These include enhanced light delivery, improved mass and charge transfer, better gas separation, improved stability, scalability, and adaptability, and new possibilities in self‐sustained hydrogen production. For example, hydrogels have been engineered into floatable matrices to immobilize photocatalysts for hydrogen production. One such floating solar hydrogen generator features a bilayer design: the bottom layer, made from a polyurethane (UHP) and poly(propylene glycol) (PPG) hydrogel, serves as a supporting layer and ensures buoyancy, while the upper layer functions as the photocatalytic component, consisting of the same UPH/PPG hydrogel but embedded with Pt/TiO_2_ nanoparticles (**Figure** **11**a). This porous hydrogel‐based floating bilayer structure creates a stable water‐splitting reactor at the water‐air interface. Due to the hydrophilic nature of the UPH/PPG hydrogel, the bottom layer continuously draws and pumps reactant (water) from the bulk reservoir to the photocatalytic interface. Meanwhile, the upper photocatalytic layer is engineered to float entirely above the water surface, avoiding the inevitable but significant reduction in light intensity that occurs underwater and thereby maximizing the light delivery. The floating configuration also favors efficient hydrogen gas escape into the air, effectively facilitating gas separation and suppressing the back‐oxidation of H_2_. Collectively, these features contribute to a two‐fold increase in the H_2_ evolution rate compared to the submerged nanocomposites (163 mmol h^−1^ m^−2^ vs 77.2 mmol h^−1^ m^−2^). Moreover, this floating structure, along with the high durability of the UPH/PPG hydrogel, ensures the generator's structural stability and long‐term operation. Given the economic and scalable fabrication of hydrogel, this floating solar hydrogen generator shows great promise for practical and large‐scale applications. This catalyst‐embedded hydrogel successfully generated hydrogen in seawater and remained stable even after 14 days. A 1 m^2^ prototype of the hydrogel containing low‐cost Cu/TiO_2_ photocatalysts was found to produce 79.2 ml of hydrogen in a day under natural sunlight.^[^ ^52^ ^]^

In a similar approach, Ho's group developed a 3D hydrogel framework through an ice‐templated assembly of TiO_2_/Ag nanofibers, and chitosan polymer for photocatalytic H_2_ generation at the water‐air interface. The resulting structure exhibits a 3D porous network with vertically aligned channels. This open, porous architecture allows photoactive materials to be directly exposed to light, minimizing light shielding and scattering while improving penetration, thereby enhancing light delivery. Additionally, the aligned microchannels facilitate efficient water transport and hydrogen release, significantly boosting hydrogen generation performance.^[^ ^306^ ^]^ Notably, these aligned microchannels introduce an additional mechanism for solar light harvesting. For example, a Cu‐ZnS/Chitosan hydrogel with directionally aligned microchannels, fabricated via ice‐templating and directional freeze casting, enables water streaming through the microchannels, driven by solar evaporation, to generate a streaming potential. This allows the hydrogel to produce electricity simultaneously with the Cu–ZnS‐mediated photocatalytic hydrogen generation, functioning as a co‐generator of hydrogen and electricity.^[^ ^53^ ^]^

The photocatalyst‐embedded hydrogel ensures close contact between water, catalyst, co‐catalyst, and sacrificial agent, effectively shortening the charge transfer pathways. This intimate interface facilitates more efficient charge transport and separation, reduces charge recombination, and ultimately enhancing photocatalytic hydrogen production. In some cases, transforming photocatalysts from a nanoparticle form into hydrogels can improve the charge transfer and separation, to such an extent that the use of co‐catalysts, typically essential for hydrogen evolution, becomes optional rather than necessary. For example, the CdSe/CdS do‐in‐rod nanocrystals (NCs), a representative heterostructured photocatalyst with a quasi‐type II electronic structure, typically require metal‐based co‐catalysts to efficiently extract photo‐excited electrons for hydrogen generation. However, this requirement changes when the CdSe/CdS NCs are transformed into a 3D, highly porous hydrogel network through a controlled destabilization of a ligand‐stabilized nanocrystal solution. While preserving the optical and electronic properties of NC building blocks, the hydrogel introduces extensive crystal‐to‐crystal contact. This structural transformation results in significantly enhanced charge carrier lifetime, mobility, and separation. The improved longevity and separation of charge carriers boost surface reaction efficiency within the hydrogel network (Figure 11b). As a result, the CdSe/CdS hydrogel, without adding any metal‐based cocatalyst, achieves a hydrogen evolution rate approximately five times higher than that of the original CdSe/CdS NC particles.^[^ ^309^ ^]^

Hydrogels can also be readily engineered into smart materials that respond to external stimuli by altering their structures and properties.^[^ ^310^ ^]^ Incorporating such smart hydrogels in hydrogen generation systems enables the development of stimuli‐responsive hydrogen generators with enhanced environmental adaptability. For example, a thermo‐responsive polymer, poly(N‐isopropyl acrylamide) (PNIPAM), has been integrated with gold‐decorated graphitic carbon nitride (Au/g‐C_3_N_4_) to create a smart photocatalytic hydrogel.^[^ ^308^ ^]^ In this system, PNIPAM undergoes a reversible volume shrinking when the temperature exceeds 32 °C, a threshold easily reached under light irradiation due to the photothermal effect of Au nanoparticles. This light‐induced, reversible shrinking/expanding allows the hydrogel‐based reactor to “track” the light, dynamically reorienting its light‐receiving surface toward the light source and thereby enhancing its photocatalytic efficiency (Figure 11c). In addition to the stimuli‐responsiveness, hydrogels are known for their exceptional water‐retention capabilities. They can store large amounts of water within their polymeric networks and even replenish themselves by absorbing moisture from the air, enabling a continuous water supply for photocatalytic hydrogen generation. As a result, integrating hydrogels into these systems opens the door to self‐sustained water‐splitting processes, eliminating the traditional reliance on bulk aqueous reservoirs. For example, the PNIPAM hydrogel has been demonstrated to function as a solid water reservoir, supplying sufficient water for Pt/g‐C_3_N_4_‐mediated hydrogen production. This eliminates the need for an aqueous suspension, enabling the Pt/g‐C_3_N_4_‐embedded PNIPAM system to operate freely in ambient air. Moreover, PNIPAM's ability to capture atmospheric moisture offers a promising strategy for photocatalytic H_2_ generation directly from water vapor.^[^ ^307^ ^]^

Although various catalysts have been incorporated into hydrogels, the rigid and brittle nature of most traditional catalysts limits the realization of a fully soft hydrogel photocatalytic hydrogen generation system. Inspired by the photosynthetic organelles, or chloroplasts of plants, where all molecules for light absorption, charge transport, and catalysis are intricately integrated and co‐localized to optimize photosynthetic efficiency, researchers have sought to develop soft and shapeable photocatalytic materials. Emulating this natural design, 2D nanoribbons formed from a supramolecular perylene derivative can self‐assemble into hydrogels containing more than 99% water through the electrostatic screening effect when charged species such as the modified Ni‐phosphine catalyst, are added.^[^ ^311^, ^312^ ^]^ This hydrogel system effectively integrates light‐harvesting units (the perylene derivative) with molecular catalysts in a single framework. Upon the addition of sacrificial agents, such as ascorbic acid, the illuminated hydrogel can drive H_2_ production with a turnover number of 340, the number of H_2_ molecules generated per catalyst molecule. This work represents a synthetic feat to mimic the intricate nature model for designing catalyst‐integrated soft materials. Similarly, a coordination polymer gel, known as Zn‐TPY‐ANT has been developed through a self‐assembly process for visible‐light‐driven photocatalytic hydrogen production. In this gel, ANT functions as the light‐harvesting moiety, while Zn‐TPY is the catalyst. Notably, the photosensitizer and catalyst are covalently linked, facilitating H_2_ evolution reactions without the need for additional cocatalysts, which are typically required in many photocatalytic H_2_ generation systems.^[^ ^313^ ^]^

#### 3.4.2 Photothermal Energy Harvesting

Photothermal materials can be embedded into hydrogels to convert light into heat, and the phenomenon can be explained by one of these three mechanisms: 1) localized surface plasmon resonance (LSPR) effect, 2) electron‐hole generation and nonradiative relaxation, or 3) molecular vibrations.^[^ ^314^ ^]^ In the LSPR photothermal effect, plasmonic metal nanoparticles absorb light of a certain wavelength range, causing the electrons to oscillate and be excited to a higher energy state. Electron‐electron scattering then occurs, leading to the redistribution of hot electrons. The heat is subsequently transferred to the lattice through electron‐phonon coupling, and this heat is then released to the surrounding medium through phonon‐phonon coupling. Metallic nanostructures such as Cu, Au, and Ag nanoparticles exhibit plasmonic resonance when irradiated with light.^[^ ^153^ ^]^ In electron‐hole generation and nonradiative relaxation, electron‐hole pairs are generated when a semiconductor material is excited by photons with energy close to the bandgap. When the excited electrons subsequently undergo nonradiative relaxation, they release phonons instead of photons to the material lattice, leading to a temperature increase in the lattice and surrounding medium.^[^ ^315^ ^]^ In molecular vibrations, the loose π electrons in carbonaceous materials such as carbon nanotubes (CNTs) and graphene are excited from the ground state (highest‐occupied molecular orbital, HOMO) to a higher energy state (lowest‐unoccupied molecular orbital, LUMO). Heat energy is then released when the excited electrons relax back to the ground state through vibration–electron coupling.^[^ ^154^ ^]^

Housing photothermal materials within a hydrogel matrix, termed photothermal hydrogels, offers several advantages for photo‐to‐thermal conversion. Firstly, the hydrogel ensures uniform dispersion, prevents agglomeration, and hence enhances the structural stability and longevity of the photothermal materials. For the practical application of photothermal and antibacterial therapy of Cu nanoparticles, Chen et al.^[^ ^316^ ^]^ utilized a guar gum hydrogel to achieve uniform distribution and improved stability of Cu nanoparticles. Their study found that embedding Cu nanoparticles within the hydrogel network does not compromise their photothermal conversion properties, as evidenced by a rapid temperature elevation under laser irradiation. Additionally, the hydrogel was found to further stabilize the Cu nanoparticles. After the photothermal process, Cu nanoparticles embedded in the hydrogel remained intact within the hydrogel, with no observable agglomeration. In contrast, Cu nanoparticles dispersed in an aqueous suspension exhibited irreversible agglomeration upon laser irradiation due to increased particle collisions, significantly impairing their photothermal efficiency.

Another advantage of hydrogels is their low thermal conductivity. The polymer matrix tends to be a poor conductor of heat,^[^ ^317^ ^]^ hence, embedding photothermal materials within hydrogels can retard heat loss to the surrounding environment. This allows the generated photothermal energy to be retained within the hydrogel, achieving a larger temperature increase under illumination. Su et al. developed an NIR light‐responsive hydrogel comprising genetically engineered anionic protein, chitosan, and Ag_3_AuS_2_ nanoparticles for in situ cancer therapy.^[^ ^318^ ^]^ The chitosan‐Ag_3_AuS_2_ hydrogel has a higher photothermal conversion efficiency (39.0%) compared to the pristine Ag_3_AuS_2_ nanoparticles (35.6%), which was attributed to the confinement effect of the compact hydrogel network and its low thermal conductivity, reducing heat loss to the environment.

Besides encapsulating photothermal materials, the hierarchical structure of hydrogels enhances light capture, further improving photo‐to‐thermal conversion. Luo et al.^[^ ^319^ ^]^ utilized a microstructured black silicon surface as a mold to create a CNT/hydrogel thin film with surface texturing. The micron‐sized spikes obtained on the CNT/hydrogel surface altered the angle of reflection, increasing forward scattering and multiple reflections, resulting in increased absorption and a higher temperature achieved through the photothermal effect.

Photothermal hydrogels can be integrated into the hot side of the thermoelectric and thermogalvanic devices to accentuate the temperature increase under illumination, leading to enhanced power output. Shen et al. fabricated a solar‐driven photo‐thermo‐electric hydrogel that can rapidly convert solar‐generated heat into thermoelectric ions for enhanced electricity generation.^[^ ^320^ ^]^ The photothermal layer was produced by crosslinking pyrogallic acid (PA) and polyethyleneimine (PEI) through oxidation by Fe(CN)_6_^3−^, while the thermoelectric hydrogel (TEH) consisted of polyacrylamide and carboxymethylcellulose with Fe(CN)_6_^3−^/ Fe(CN)_6_^4−^ as the redox couple. The dark‐coloured PA‐PEI‐Fe photothermal layer converted the sunlight into heat, boosting the thermoelectric output. Moreover, the photothermal layer can also protect the Fe(CN)_6_^3−^/ Fe(CN)_6_^4−^ redox couple from ultraviolet damage. Similarly, Li's group developed a quasi‐solid hydrogel‐based thermo‐electrochemical cell by introducing carboxymethylcellulose (CMC) into a polyacrylamide network.^[^ ^321^ ^]^ A photothermal CNT coating on the cell enabled robust solar‐thermal‐electricity conversion, leading to enhanced power output.

In many of these cases, photothermal hydrogels can also function as solar evaporators to produce water, steam, or freshwater. Fan et al. ^[^ ^322^ ^]^ developed a multi‐layered steam‐electricity cogeneration system, with CuO micro‐flowers on Cu foil as the photothermal material, and a polyacrylamide (PAAM) hydrogel as the reactor for temperature gradient‐triggered redox reactions to generate electricity, as well as a seawater reservoir for steam generation. Upon exposure to solar irradiation, the photothermal layer converts light into heat, creating a temperature gradient across the PAM hydrogel electrolyte, which initiates redox reactions to produce a thermogalvanic voltage output. Simultaneously, the photothermal heat can also promote evaporation of the water contained in the upper PAM hydrogel. Jia et al. utilized tea leaf residue as a biomass‐based photothermal material to fabricate a photothermal PVA hydrogel, which is integrated with a thermoelectric module to maximize the utilization of waste heat energy produced during evaporation. This water‐electric cogeneration device could produce an evaporation rate of 0.91 kg m^−2^ h^−1^ and a stable voltage output of 58.8 mV.^[^ ^323^ ^]^ The photothermal effect can be extended even further into a photothermal water‐electricity‐hydrogen multi‐function concept. Zhou et al. developed a polyacrylamide (PAM) hydrogel containing CNTs and coupled it with a thermoelectric generator.^[^ ^324^ ^]^

When hydrogel‐based solar evaporation or steam generation (Figure 11d) is discussed, it has to be noted that hydrogels, in addition to the inherent advantages of photothermal hydrogels, offer several unique benefits for highly effective water purification and energy harvesting.^[^ ^49^, ^325^, ^326^ ^]^ One such benefit is a low water vaporization enthalpy of hydrogels, due to the presence of intermediate water within the hydrogel network. This feature reduces energy demand and enhances the efficiency of water evaporation. Another key attribute is the high water retention and transport capabilities of hydrogels, which promote a strong interaction between light absorbers and water molecules, facilitating efficient mass exchange and hence accelerating water evaporation. Additionally, the versatility in structure and properties modification of hydrogels allows for the incorporation of features favorable for solar evaporation, such as antifouling^[^ ^327^, ^328^, ^329^ ^]^ and aligned channels.^[^ ^330^, ^331^ ^]^ Interestingly, photothermal hydrogels with aligned nanochannels can also trigger the streaming potential mechanism during photothermal evaporation, enabling the simultaneous conversion of light energy into useful electricity.^[^ ^53^, ^135^, ^252^ ^]^

## 4 Hydrogel Innovation for Environmental Energy Harvesting

In the previous section, we surveyed hydrogel‐assisted technologies capable of capturing and converting various forms of ambient energy, including mechanical, thermal, chemical, and solar into electricity and clean fuels. We highlighted the unique physicochemical properties of hydrogels that make them especially suited for environmental energy harvesting. To complement this broad overview, the following section, drawing primarily on our group's recent research on functional hydrogels, offers insights into recent advancements in the design and innovation of hydrogels for such applications. Emerging research trends in this field include: 1) ensuring biocompatibility and biodegradability, 2) engineering hydrogel structures, and 3) enhancing the integration of hydrogel‐based systems.

### 4.1 Ensuring Biocompatibility and Biodegradability

In recent years, the convergence of electronics and biology has driven transformative advancements across fields such as environmental monitoring,^[^ ^332^, ^333^ ^]^ healthcare,^[^ ^334^ ^]^ wearable, and implantable technologies.^[^ ^335^ ^]^ As electronics become more integrated into both biological systems and natural environments, the need for biocompatible and biodegradable electronics has become more pressing. These emerging systems not only promise safer and more sustainable alternatives to traditional electronics but also open up innovative applications that were previously limited by issues such as material toxicity, mechanical rigidity, and long‐term environmental persistence.^[^ ^336^, ^337^, ^338^, ^339^, ^340^, ^341^ ^]^

In the context of environmental energy harvesting, incorporating biocompatibility and biodegradability into electronic systems offers several advantages. These materials allow for safe and seamless integration with both natural environments and biological systems such as human skin, tissues, or internal organs, enabling more accurate and responsive interactions. Moreover, such systems can operate without reliance on conventional power sources like batteries or bulky circuits, promoting minaturization, portability, and long‐term autonomous operation.^[^ ^342^ ^]^ Most importantly, once their functional lifespan comes to an end, these devices can be degraded, particularly in a controlled manner, reducing environmental impact and minimizing harm to living organisms. This makes them especially valuable for temporary applications, such as biodegradable implants or disposable environmental sensors, aligning technological advancement with ecological and biomedical responsibility.^[^ ^336^, ^343^ ^]^

**Figure 12:**
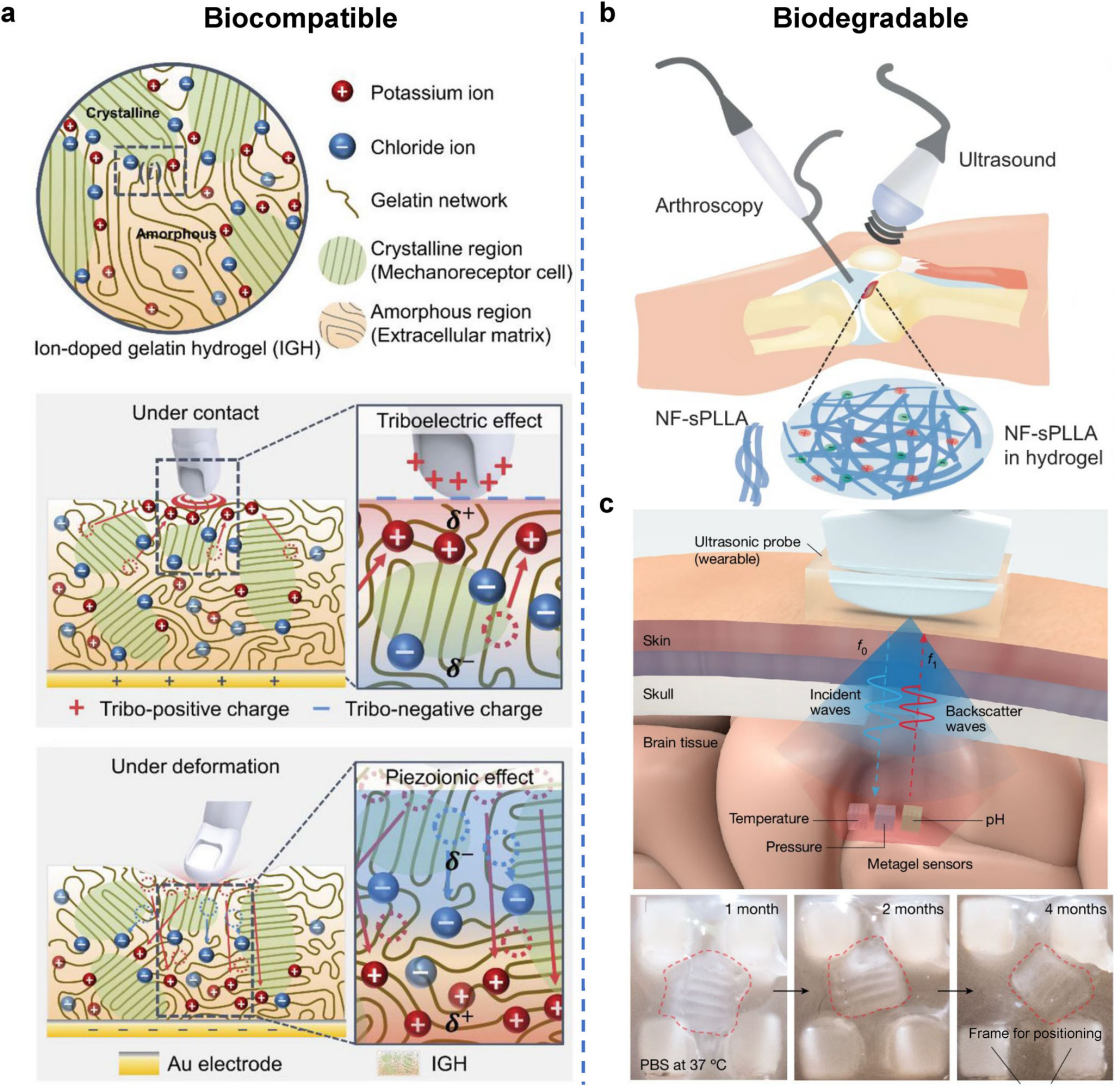
a) Schematic illustration of the IGH with cation‐anion pairs ([K^+^][Cl^−^]) distributed in the gelatin networks, exhibiting triboelectric and piezoionic effect. Reproduced with permission.^[^ ^346^ ^]^ Copyright 2021, Wiley‐VCH. b) Schematic illustration of the use of piezoelectric hydrogel for osteoarthritis patients. Reproduced under the terms of the CC‐BY Creative Commons Attribution 4.0 International license (https://creativecommons.org/licenses/by/4.0).^[^ ^347^ ^]^ Copyright 2023, The Authors, published by Springer Nature. c) Diagram showing metagels as wireless intracranial physiology sensors using ultrasound reflection. Photographs showing a metagel degrading following 1 month in PBS solution at 37 °C; dashed line delineates the shape of the metagel. Reproduced with permission.^[^ ^341^ ^]^ Copyright 2024, Springer Nature.

To achieve this goal, designing energy generators using biocompatible and biodegradable materials offers a straightforward yet effective strategy. Natural biomaterials such as collagen, gelatin, chitosan, alginate, cellulose, silk fibroin, as well as biodegradable synthetic polymers like poly(L‐lactic acid), poly(lactic‐co‐glycolic acid), and poly(3‐hydroxybutyrate‐co‐3‐hydroxyvalerate) have been utilized in constructing environmentally and biologically friendly energy harvesting devices.^[^ ^339^, ^344^, ^345^ ^]^ **Figure** **12**a illustrates a triboelectric and piezoionic energy generator composed of gelatin and a KCl solution.^[^ ^346^ ^]^ Gelatin, a natural peptide‐based material, is known for its non‐toxicity, biocompatibility, biodegradability, and low cost. KCl, a biologically relevant salt, plays essential roles in physiological processes across many organisms. The use of these materials ensures that the resulting generator is both environmentally and biologically friendly. This example also highlights the versatility of hydrogel systems, where multiple energy conversion mechanisms, such as the triboelectric and piezoionic, shown in Figure 12a, can operate simultaneously within a single platform. The use of biocompatible and biodegradable hydrogels, such as poly‐L‐lactic acid (PLLA)/collagen system in Figure 12b, enables the precise delivery of (piezo)electric stimulation (ES) at the defect site to promote bone healing and regeneration within the body.^[^ ^347^ ^]^ The degradation period of this PLLA/collagen‐based piezoelectric generator can be engineered to last 1–2 years, after which it safely breaks down into non‐toxic byproducts. Note that the degradation rate of hydrogels can be readily tuned by adjusting factors such as composition, structure, and crosslinking density.^[^ ^348^ ^]^ This tunability is crucial for tailoring the functional lifespan of bioelectronic devices. For instance, the hydrogel used for in vivo brain tissue monitoring in Figure 12c was specifically designed to degrade within approximately four months.^[^ ^341^ ^]^

### 4.2 Engineering Hydrogel Structures

Engineering the structure of hydrogels is critical in the context of energy generation because their physical architecture directly influences their interactions with environmental stimuli, energy conversion efficiency, material properties, responsiveness, and integration with target environments. Recently, many structural configurations, such as fibrous,^[^ ^349^, ^350^, ^351^ ^]^ asymmetric,^[^ ^352^, ^353^ ^]^ gradient,^[^ ^286^ ^]^ ordered,^[^ ^354^ ^]^ patterned,^[^ ^355^ ^]^ and biomimetic^[^ ^356^, ^357^ ^]^ designs have been strategically employed to enhance performance in energy‐harvesting systems. In this section, several of our recent works are presented as case studies to illustrate the functional possibilites enabled by structural engineering.

**Figure 13:**
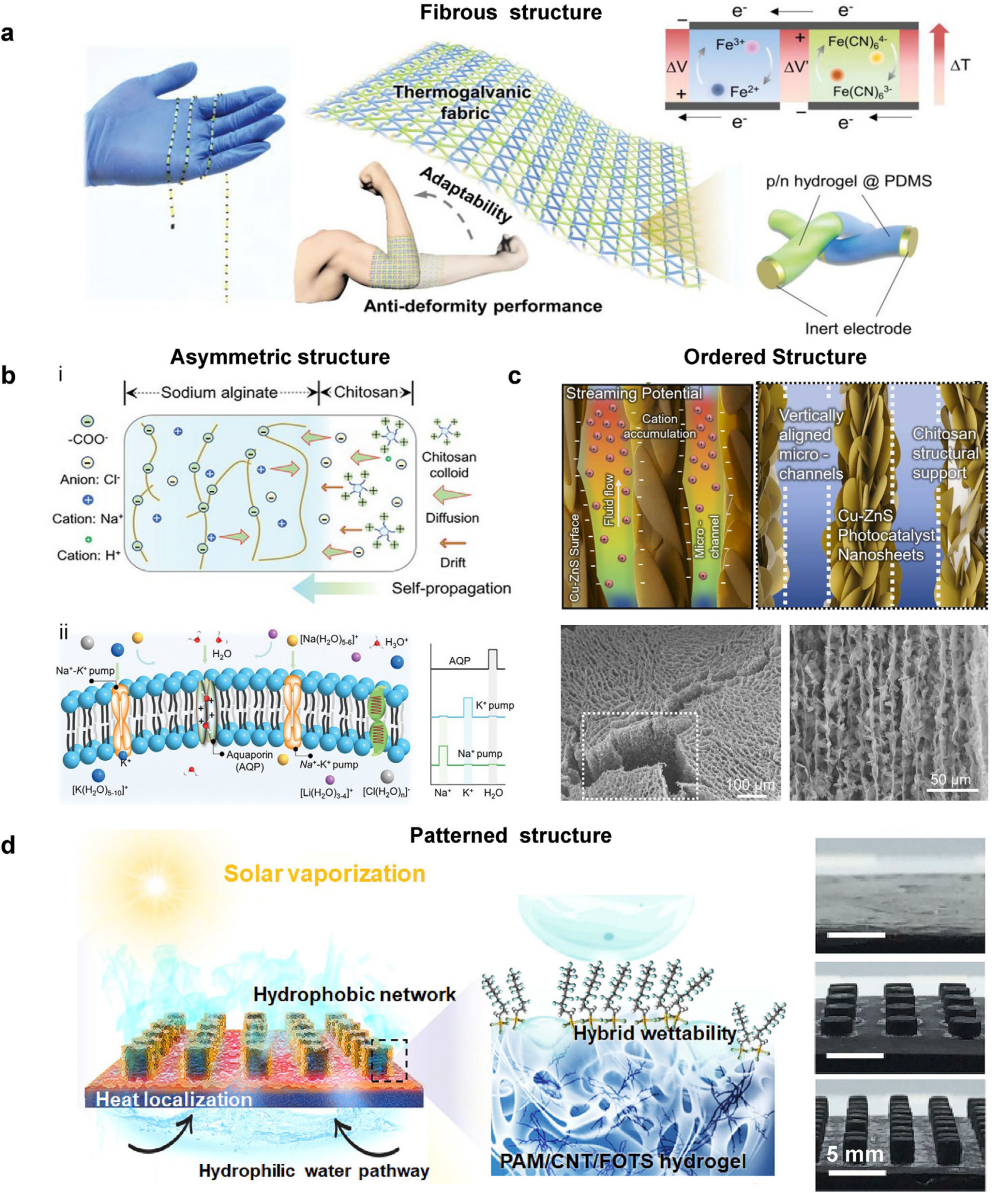
a) PAAm hydrogel with redox couples, Fe(CN)_6_^3−^/Fe(CN)_6_^4−^ and Fe^2+^/Fe^3+^ for thermogalvanic fiber‐woven textile to harvest body heat from irregular and dynamically moving skin surfaces. Reproduced with permission.^[^ ^27^ ^]^ Copyright 2021, Wiley‐VCH. b, i) Fabrication of an asymmetric hydrogel analogous to a p‐n junction using the self‐propagative flow approach which relies on the diffusion of a low‐viscosity chitosan colloid (polyanion) into a high‐viscosity sodium alginate solution (polycation), accompanied by a coacervation reaction. ii) Functional resemblance to cytomembrane in mediation of spatiotemporal transportation of media and signals. Reproduced with permission.^[^ ^358^ ^]^ Copyright 2023, Wiley‐VCH. c) Cu‐ZnS‐embedded hydrogel with vertically aligned channels by directional freeze casting to harvest streaming potential energy. Reproduced with permission.^[^ ^53^ ^]^ Copyright 2024, Elsevier. d) Hydrogel‐based pillars array solar evaporator composed of PAAm and CNT to optimize water pumping rate and boost steam generation efficiency. Reproduced with permission.^[^ ^324^ ^]^ Copyright 2020, Elsevier.

The human body is one of many abundant sources of low‐grade heat in the environment. Textile electronics, with their wearable and conformable designs, hold great promise for harvesting this thermal energy and converting it into useful electricity through close and continuous contact with the heat source. To develop such textiles, hydrogels are usually fabricated into fibres and then woven together for body heat harvesting. For this purpose, we reported a textile made of polyacrylamide (PAAm) hydrogel‐based thermogalvanic fibers (**Figure** **13**a). In these fibers, PAAm hydrogel works as the matrix to host redox couples, Fe(CN)_6_^3−^/Fe(CN)_6_^4−^ and Fe^2+^/Fe^3+^, forming p‐type and n‐type thermogalvanic segments, respectively. These p‐type and n‐type segments are connected through a scalable, modular assembly approach, enabling the efficient fabrication of thermogalvanic fibers. The resulting fibers inherit the superior stretchability and elasticity of their hydrogel components, ensuring intimate and conformal contact with the human body. Unlike traditional rigid or substrate‐supported thermocells, the thermogalvanic fiber‐woven textile is uniquely capable of harvesting body heat from irregular and dynamically moving skin surfaces. Notably, it generates an open‐circuit voltage of approximately 0.7 V in cold ambient conditions and up to 0.5 V on a dynamically flexing elbow.^[^ ^27^ ^]^

In 2023, we used a self‐propagating flow approach to fabricate asymmetric hydrogels (analogous to a p–n junction) by diffusing a low‐viscosity chitosan colloid into high‐viscosity sodium alginate, triggering a coacervation reaction.^[^ ^358^ ^]^ This process results in the formation of a polyelectrolyte complex hydrogel with two ends exhibiting distinct properties, as illustrated in Figure 13b. Unlike many post‐assembled asymmetric hydrogels, this hydrogel inherently mitigates potential interface instability, thereby enhancing structural integrity and long‐term stability. More intriguingly, this asymmetric hydrogel not only exhibits a passive transcellular‐like potential between its two ends, but also demonstrates dynamic, ion‐selective electro‐responses. These dynamic behaviors primarily arise from differences in ionic diffusion rates at the interfaces between liquid (water or salt solution) and the respective chitosan or sodium alginate ends. As such, this asymmetrically structured hydrogel uniquely combines both passive and dynamic electro‐responses within a single structure, functionally resembling the cytomembrane in its ability to mediate spatiotemporal transportation of media and signals. This work represents a significant advancement beyond current artificial systems, which typically display only simple and passive transcellular potentials. As a soft power source, a single unit of this asymmetric hydrogel can generate an output power density of 135–190 mW m^−2^, outperforming most bio‐inspired soft and green energy harvesters. In addition, we developed a moisture energy generator (MEG) based on an asymmetrically structured hydrogel composed of two oppositely charged, compact planar hydrogel films.^[^ ^50^ ^]^ This asymmetric charge distribution endowed the MEG with high moisture sensitivity, even under relative humidity (RH) levels below 20%, as well as continuous operation at 100% RH, while maintaining structural integrity and long‐term stability.

In addition to designing asymmetric structures, we also engineered hydrogels with ordered architectures. For example, in 2024, a hydrogel featuring vertically aligned channels was developed to harvest the streaming potential energy (Figure 13c).^[^ ^53^ ^]^ Using a combination of directional freeze casting and ice templating, a Cu‐ZnS‐embedded hydrogel was fabricated with vertically aligned internal channels. Akin to the transpiration process in plants, these internal channels facilitate directional fluid transport within the hydrogel, particularly under solar light irradiation, where a liquid is drawn upward from the bottom to the top via evaporation and capillary wicking. As the fluid flows through the hydrogel and interacts with the Cu‐ZnS surface, this material becomes charged, attracting counter‐ions of opposite polarity to its proximate surface. The movement of fluid along the channels transports these counter‐ions, generating an ionic concentration gradient that drives the formation of a streaming potential. Additionally, these vertically aligned channels enhance the photocatalytic performance of the Cu‐ZnS‐embedded hydrogel by facilitating rapid delivery of reactants to the Cu‐ZnS photocatalytic sites and efficient release of the generated gases. This Cu‐ZnS‐embedded hydrogel was designed as an electricity‐hydrogen cogenerator, by leveraging the high tunability of hydrogels to innovatively integrate the functional requirements of multiple technologies into one single hybrid structure.

Moreover, we also developed a CNT‐PAAm hydrogel featuring a patterned pillar array designed to regulate surface water distribution in an interfacial solar evaporator.^[^ ^324^ ^]^ This structural design balances water availability, preventing surface cooling caused by excessive water and avoiding dehydration and heat loss due to insufficient water supply. As shown in Figure 13d, the entire hydrogel under solar irradiation serves a dual function as both a light absorber and a water evaporator. The CNT‐PAAm hydrogel absorbs solar energy to heat the water it draws from a bulk reservoir, converting it into steam. The pillars, with their increased surface area, act as the primary water evaporation sites. Furthermore, molecular engineering was employed to tailor the hydrogel's wettability, resulting in a desirable pattern: the inner region of the hydrogel remains hydrophilic to efficiently draw water toward the evaporation sites, while the outer surface is rendered hydrophobic to control water on the evaporator surface. This hydrophilic/hydrophobic patterned, pillar‐structured hydrogel evaporator achieves a well‐regulated balance between water availability and interfacial heating, resulting in an enhanced water evaporation rate of 1.42 kg m^−2^ h^−1^.

### 4.3 Enhancing the Integration of Hydrogel‐Based Systems

**Figure 14:**
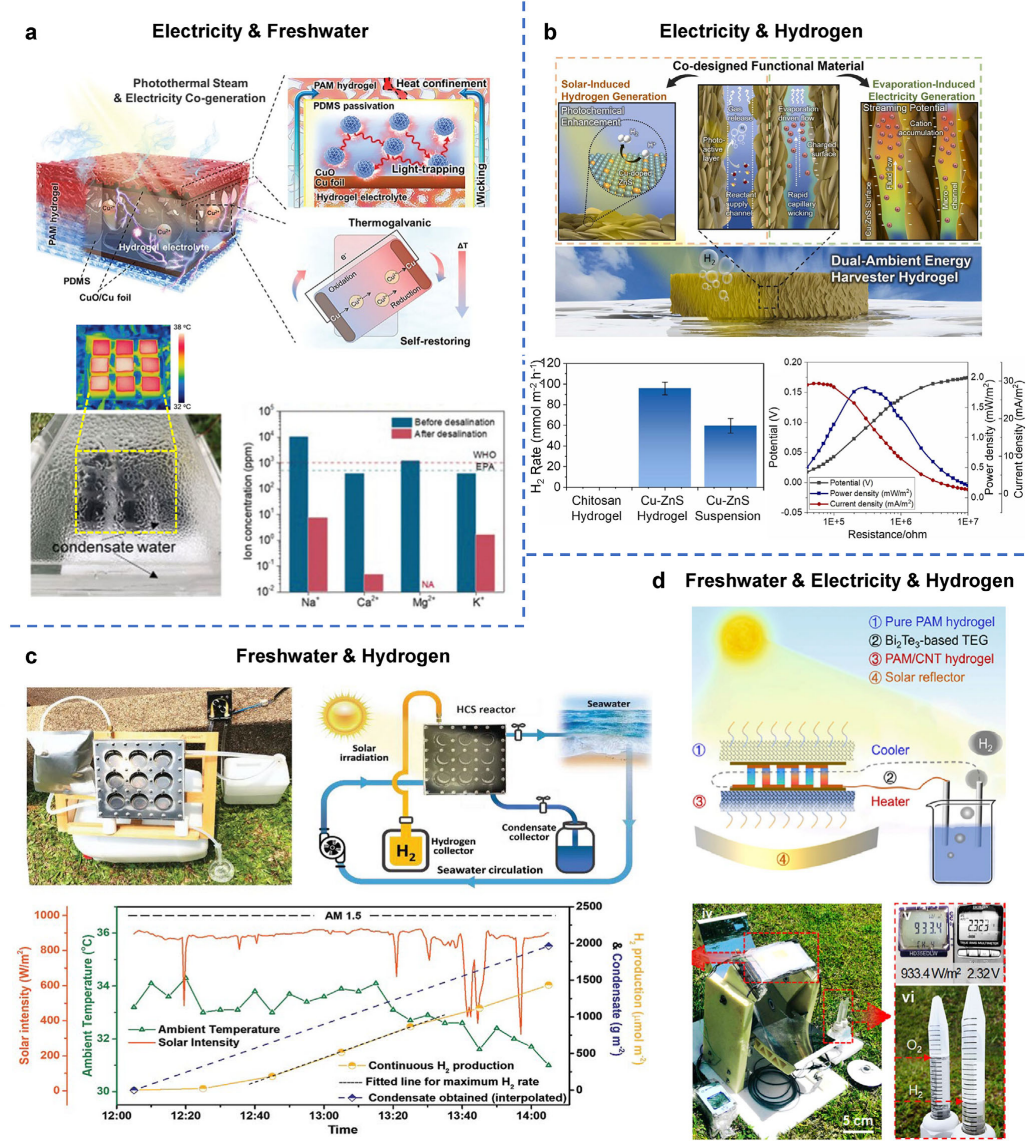
a) Hybrid photothermal steam and energy generator for electricity‐freshwater co‐generation. Reproduced with permission.^[^ ^322^ ^]^ Copyright 2020, Wiley‐VCH. b) Cu‐ZnS‐embedded hydrogel for electricity‐hydrogen co‐generation. Reproduced with permission.^[^ ^53^ ^]^ Copyright 2024, Elsevier. c) TiO_2_/Ag nanofiber‐embedded chitosan hydrogel for freshwater‐hydrogen co‐generation. Reproduced with permission.^[^ ^306^ ^]^ Copyright 2020, Wiley‐VCH. d) PAAm and CNT hydrogel‐based pillars array solar evaporator for freshwater‐electricity‐hydrogen co‐generation. Reproduced with permission.^[^ ^324^ ^]^ Copyright 2020, Elsevier.

From previous discussions, it is evident that most of the hydrogel‐based energy generators developed by our group are hybrid systems, designed to harness and convert multiple forms of energy rather than relying on a single energy conversion. For example, the moisture electricity generator (MEG), based on the asymmetrically structured hydrogel, not only generates electricity from ambient moisture but also functions as a freshwater generator by capturing and condensing atmospheric water, effectively creating an electricity‐freshwater cogeneration system.^[^ ^50^ ^]^ Similarly, photothermal, photocatalytic, or thermogalvanic hydrogels have been demonstrated as cogenerators for various combinations such as electricity‐freshwater,^[^ ^322^ ^]^ freshwater‐hydrogen,^[^ ^306^ ^]^ electricity‐hydrogen,^[^ ^53^ ^]^ and freshwater‐electricity‐hydrogen^[^ ^324^ ^]^ (**Figure** **14**). These hybrid system designs reflect one of our core research philosophies: systems thinking.^[^ ^49^, ^359^, ^360^, ^361^, ^362^ ^]^ Guided by this approach, we emphasize integration, interconnection, and interdependence between different technologies. By combining multiple energy harvesting mechanisms, we create sustainable frameworks where inefficiencies in one system can be transformed into advantages in another. This interconnectedness synergizes the overall performance of the functionality of the entire system.

Take, for instance, the MEG based on the asymmetric hydrogel.^[^ ^50^ ^]^ Moisture absorption triggers ion liberation and establishes an ionic gradient that varies with the degree of moisture absorbed and eventually dissipates once the hydrogel reaches saturation. However, by incorporating photothermal materials, like carbon nanotubes (CNTs) into the hydrogel, solar irradiation induces the evaporation of absorbed moisture, which then condenses as freshwater. This moisture desportion alters the ion concentration, effectively rebuilding the ionic gradient. In this way, electricity generation becomes synchronized with the fundamental processes of atmospheric water harvesting, the absorption and desorption of moisture. Through this simple integration of CNTs, MEG, photothermal heating, and atmospheric water harvesting technologies become interconnected, contributing to a multifunctional output.

Another example is the integration of ionic thermogalvanic technology into a solar evaporation system.^[^ ^322^ ^]^ In this setup, a hydrogel‐based ionic thermocell is placed between a solar absorber layer and a bulk water reservoir. In conventional solar evaporation systems, solar irradiation creates a temperature gradient between the absorber and the bulk water, potentially resulting in heat loss. However, by inserting a thermogalvanic cell in this interface, the temperature gradient can be harnessed to generate electricity while simultaneously reducing heat loss. This principle has also been demonstrated in other systems, where commercial thermoelectric modules are integrated into solar evaporators for similar dual‐functionality.^[^ ^7^, ^324^, ^362^ ^]^

In most photocatalytic hydrogel‐based multifunctional systems, hybridization also enables efficient utilization of the full solar spectrum. Short‐wavelength photons drive photocatalytic reactions, while long‐wavelength photons contribute to photothermal heating.^[^ ^306^, ^363^ ^]^ This comprehensive energy capture further exemplifies the systems thinking approach that underlines our research, where integration not only adds functionality but also amplifies overall system efficiency.

To conclude, this work highlights how hybrid hydrogel systems embody a holistic design framework that shifts from single‐function devices toward integrated, multifunctional solutions. By combining diverse energy harvesting pathways, these synergistically designed systems demonstrate the power of coupling materials innovation and structural architecture with system‐level integration. Such an approach can mitigate conflicting effects between components, transforming them into mutually supportive interactions that enhance overall device efficiency.

## 5 Conclusions and Outlook

In this review, we have systematically outlined synthesis strategies and the fundamental physicochemical properties of hydrogels, highlighting their unique advantages as functional materials for environmental energy harvesting. We have delved into discussions on a series of energy harvesting technologies capable of converting mechanical, thermal, chemical, and solar, into electricity or clean fuels, and particularly examined the distinctive roles that hydrogels play in enabling or enhancing these energy conversions. Through representative case studies and recent advancements, it is evident that hydrogels not only rival but often outperform many conventional materials in these applications. Their intrinsic characteristics—such as 3D porous structure, high water content, biocompatible, and exceptional structural and property tunability—confer multifunctionality and adaptability across both natural and biological environments. Furthermore, we have also presented our group's progress in the development of hydrogels, and hydrogel‐based energy harvesting systems, offering insights drawn from years of research in functional nanomaterials for sustainable energy and fuel generation. These efforts collectively underscore the promise of hydrogels as a transformative platform for next‐generation energy technologies that are not only environmentally friendly but also economically viable and socially impactful.

Nevertheless, key challenges remain. A comprehensive understanding of the fundamental mechanisms governing energy conversion in hydrogel systems is still lacking. Moreover, issues such as materials stability, substantial and long‐term performance in practical environments, and seamless integration with existing energy infrastructure and physiological systems continue to pose significant hurdles. To address these challenges, future research should aim to establish standardized evaluation protocols, utilize advanced characterization techniques, and incorporate artificial intelligence (AI) to model, predict, and optimize hydrogel‐environment interactions. Such approaches could facilitate a deeper understanding of energy conversion mechanisms at the molecular or even atomic scale. Looking ahead, the continued interdisciplinary convergence of materials science, chemistry, bioengineering, and data science will be pivotal in unlocking the full potential of hydrogels for sustainable energy applications. The following sections outline key perspectives derived from recent advances in materials innovation for sustainable energy and clean fuel generation.

### 5.1 Applying Machine Learning (ML) for Accelerated Design of Hydrogels

The design process of hydrogels can be accelerated with the help of machine learning (ML). ML is a powerful tool for analysing large datasets, identifying patterns and uncovering complex relationships between hydrogel structures and their functional properties, in turn optimising design parameters for targeted applications. Furthermore, integrating ML with hydrogel design creates a feedback loop where structural designs can be iteratively refined to meet specific performance goals, accelerating the development of application‐tailored hydrogel materials.

However, one major challenge is the limited availability of high‐quality, standardised datasets for hydrogel systems, particularly those involving dynamic or multi‐stimuli responses. Curating comprehensive and consistent datasets will be key to training reliable models. Integrating ML with physical modeling, such as finite element or molecular dynamics simulations, can improve interpretability and predictive accuracy. Additionally, the combination of ML with high‐throughput experimentation can guide the efficient exploration of large compositional and structural design spaces. Techniques like transfer learning and inverse design may further enhance the ability to discover novel hydrogel formulations optimised for specific energy harvesting functions. As these approaches mature, ML will play an increasingly important role in the intelligent design of next‐generation hydrogel materials.

### 5.2 Enabling Sustainable and Environmentally Friendly Manufacturing

Besides enhancing the design process of hydrogels, attention should also be paid to the fabrication process to ensure sustainability. Hydrogel resources should be abundant, renewable, and benign, while supporting large‐scale production and facile processing for clean fuel generation and energy conversion. Given their biodegradability and wide availability, biomass‐derived polymers such as cellulose, chitosan, and lignin are promising candidates for sustainable hydrogel fabrication. Moreover, the use of toxic or non‐degradable chemicals in the synthesis process should be avoided.

In addition to raw materials, the manufacturing process itself must be optimised for sustainability. This includes adopting green synthesis routes, minimising energy consumption, and reducing hazardous waste through solvent‐free or low‐temperature processing. Life‐cycle considerations encompassing raw material sourcing, production, use, and disposal should guide the design of recyclable, biodegradable, or regenerable hydrogel systems to support a circular materials economy.

Real‐world implementation will also require alignment with regulatory frameworks, environmental standards, and market readiness. Partnerships with industry can accelerate pilot‐scale validation, supply chain integration, and cost benchmarking, ensuring that sustainable hydrogel technologies move beyond the laboratory into impactful applications.

### 5.3 Increasing Power Output

Although hydrogels are capable of harvesting environmental energy and generating electricity, their power output remains relatively low in the range of microwatts to milliwatts per square centimeter.^[^ ^62^ ^]^ Therefore, efforts must be made to improve the performance and increase the power output. Improving performance will require a combination of targeted strategies. Structural design optimisation, such as incorporating aligned channels, asymmetric geometries, or multilayered architectures, has been shown to improve charge transport and energy conversion efficiency. In addition, materials optimisation plays a crucial role. This includes the incorporation of highly conductive fillers such as carbon nanotubes (≈10^6^ to 10^7^ S m^−1^),^[^ ^364^ ^]^ MXenes (≈10^6^ S m^−1^),^[^ ^365^ ^]^ or graphene (≈10^8^ S m^−1^)^[^ ^364^ ^]^ to improve electrical conductivity, as well as tailoring polymer networks to enhance ionic conductivity (from 20 mS cm^−1^ to 396 mS cm^−1^).^[^ ^366^ ^]^ Interfacial engineering, such as improving electrode‐hydrogel contact and minimising interfacial charge losses, can also significantly impact overall output.

Furthermore, hybridisation with complementary energy harvesting mechanisms (e.g., combining thermoelectric, moisture‐electric, and photothermal effects) can enable synergistic power generation and better utilisation of ambient energy. Finally, internal resistance is a critical factor, but hydrogels inherently exhibit high internal resistance (e.g., 25 Ω∙m),^[^ ^367^ ^]^ which limits energy output. Therefore, future efforts should focus on reducing the internal resistance of hydrogels through both materials and structural innovation to increase the power output.

### 5.4 Minimising Electrode Corrosion

The stability of the hydrogel‐based energy harvesters is also a very important factor for practical applications. Metal electrodes commonly used in hydrogel‐based energy systems are highly susceptible to corrosion due to the hydrogel's high water content, ionic conductivity, and potential pH fluctuations. These factors accelerate degradation processes such as oxidation, ion leaching, and surface passivation, which compromise both device performance and long‐term stability.

One approach to mitigating this issue is to replace metal electrodes with corrosion‐resistant alternatives such as carbon nanotube (CNT) composites. While CNT‐based electrodes offer improved durability, they often suffer from residual capacitance, which can reduce overall power output. Therefore, better solutions are needed to balance conductivity, stability, and electrochemical performance. Promising alternatives include graphene, conductive polymers, and metal oxide‐coated conductors, which provide varying degrees of corrosion resistance, flexibility, and electrical performance. Additionally, surface modification strategies, such as applying thin protective coatings or encapsulation layers, can help reduce direct electrolyte–metal contact without severely impeding electron transfer.

Looking ahead, the development of self‐healing, adaptive, or reconfigurable electrode materials may offer new avenues to extend device lifetime and reliability in dynamic or wearable energy systems.

### 5.5 Development of Advanced Characterisation Techniques

Hydrogels are typically characterised using standard techniques such as FTIR and Raman spectroscopy etc. However, these conventional methods are often insufficient to provide an in‐depth understanding of the hydrogel and its interactions with both the external environment and the internal hydrogel matrix. This inadequacy stems from the inherent complexity of the hydrogel's internal environment, where multiple dynamic interactions between the polymer network, water, and various stimuli or embedded chemicals occur simultaneously. These interactions often involve spatial heterogeneity, transient structural rearrangements, and physicochemical responses to environmental triggers that cannot be fully captured by static or conventional techniques.

To address these limitations, advanced characterisation tools such as neutron and X‐ray scattering, high‐resolution imaging (e.g., cryo‐TEM, AFM), and magnetic resonance imaging (MRI) should be employed to provide a deeper structural and mechanistic insight of the hydrogels. Moreover, in situ or operando techniques, including in situ neutron scattering, cryo‐TEM electrochemical imaging, and time‐resolved spectroscopy, can provide real‐time mechanistic insights into ion transport, structural deformation, hydration dynamics, and chemical reactivity under conditions that closely mimic actual operational environments. In particular, multi‐modal and correlative methods that integrate mechanical, electrical, and spectroscopic data could be pivotal in revealing structure–property–function relationships. Furthermore, to complement these characterisation techniques, multiscale simulation studies of the hydrogels at scales ranging from the atomic scale (few nanometers) to macroscopic scale (centimeters and beyond) should also be carried out to provide a deeper theoretical understanding of the underlying mechanism. Developing and applying these next‐generation techniques will enable the rational design of hydrogels with enhanced performance, durability, and application‐specific functionality.

### 5.6 Advancing Energy Storage Integration

As energy generation from environmental sources is often intermittent due to the external factors, it is necessary to pair hydrogel‐based generators with energy storage systems to store excess power and provide a sustained and stable power output. Since the energy generator is soft, the energy storage should also be soft in order to achieve a monolith soft‐material format. Hydrogels are promising candidates for such integration. Current research is exploring hydrogel‐based supercapacitors, stretchable batteries, and redox‐active gels as energy storage components that can be seamlessly incorporated with hydrogel harvesters. However, challenges remain in ensuring ionic and electronic conductivity compatibility, minimising interfacial resistance, and enhancing electrochemical efficiency. Advanced strategies such as interfacial engineering, ionic channel alignment, and dual‐functional material design are needed to achieve efficient energy transfer between harvesting and storage modules.

Moreover, integrating self‐healing, biocompatibility, and environmental stability will also be critical for real‐world applications such as wearable electronics or biomedical implants. The development of multifunctional hydrogel systems that can simultaneously harvest and store energy represents a promising future direction for compact and integrated soft energy devices.

### 5.7 Standardising Testing Protocols and Data Reporting

With all these possible advancements in hydrogel‐based energy harvesting, it becomes increasingly important to establish a common platform for researchers to compare their results. In the current literature, hydrogel energy generator performance is reported using a variety of metrics, making direct and fair comparisons between studies difficult. The lack of standardised performance benchmarks hinders progress and limits the ability to identify truly impactful innovations. Therefore, it is imperative to develop a consistent reporting framework that researchers can adopt to present their results and benchmark against others. Such standardisation would also provide clearly defined performance targets for the field.

Another major issue is the absence of standardised laboratory testing methodologies that can reliably predict real‐world performance. At present, most experiments are conducted in ideal, controlled environments using small sample sizes, often resulting in inflated performance outcomes. However, practical deployment conditions are typically much more complex. For example, osmotic energy harvesting studies frequently use small‐scale setups that do not reflect full‐scale operational environments.

To bridge this gap, researchers should aim to design laboratory testing protocols that closely mimic realistic environmental conditions, including fluctuating temperature, humidity, salinity, mechanical stress, and exposure to contaminants. For instance, wearable hydrogel‐based devices should be evaluated under conditions that simulate continuous body motion, perspiration, and prolonged skin contact. Similarly, hydrogel systems intended for outdoor energy harvesting should be tested under variable weather cycles, sunlight intensities, and exposure to pollutants. In addition to environmental simulation, pilot‐scale evaluations in field‐relevant settings such as integration into building materials, textiles, or water systems are essential for assessing long‐term durability, scalability, and maintenance requirements. These pilot studies can uncover performance degradation mechanisms, operational constraints, and system‐level integration challenges that are often overlooked in small‐scale lab setups. Ultimately, such comprehensive evaluation frameworks will be critical in translating laboratory breakthroughs into viable, real‐world hydrogel energy harvesting technologies.

## Conflict of Interest

The authors declare no conflict of interest.

## Author Contributions

W.L. and W.L.O. contributed equally to this work. W.L.O., W.L., X.P., Z.L., and G.T. performed the investigation and wrote the original draft; G.W.H. conceptualized and supervised the study; W.L.O., W.L., X.P., Z.L., G.T., and G.W.H. wrote, reviewed, and edited the final draft. All authors have read and agreed to the published version of the manuscript. W.L. and W.L.O. contributed to this work equally.
